# A pangenomic atlas reveals eco-evolutionary dynamics that shape type VI secretion systems in plant-pathogenic *Ralstonia*

**DOI:** 10.1128/mbio.00323-24

**Published:** 2024-08-27

**Authors:** Nathalie Aoun, Stratton J. Georgoulis, Jason K. Avalos, Kimberly J. Grulla, Kasey Miqueo, Cloe Tom, Tiffany M. Lowe-Power

**Affiliations:** 1 Department of Plant Pathology, University of California, Davis, California, USA; 2 Department of Microbiology and Molecular Genetics, University of California, Davis, California, USA; University of Hawaii at Manoa, Honolulu, Hawaii, USA; University of Wisconsin-Madison, Madison, Wisconsin, USA

**Keywords:** T6SS, mobile genetic elements, pangenome, *Ralstonia solanacearum* species complex, horizontal gene transfer

## Abstract

Soilborne *Ralstonia solanacearum* species complex (RSSC) pathogens disrupt microbial communities as they invade roots and fatally wilt plants. RSSC pathogens secrete antimicrobial toxins using a type VI secretion system (T6SS). To investigate how evolution and ecology have shaped the T6SS of these bacterial pathogens, we analyzed the T6SS gene content and architecture across the RSSC and their evolutionary relatives. Our analysis reveals that two ecologically similar Burkholderiaceae taxa, xylem-pathogenic RSSC and *Paracidovorax*, have convergently evolved to wield large arsenals of T6SS toxins. To understand the mechanisms underlying genomic enrichment of T6SS toxins, we compiled an atlas of 1,066 auxiliary T6SS toxin clusters (“*aux*” clusters) across 99 high-quality RSSC genomes. We classified 25 types of *aux* clusters with toxins that predominantly target lipids, nucleic acids, or unknown cellular substrates. The *aux* clusters were located in diverse genetic neighborhoods and had complex phylogenetic distributions, suggesting frequent horizontal gene flow. Phages and other mobile genetic elements account for most of the *aux* cluster acquisition on the chromosome but very little on the megaplasmid. Nevertheless, RSSC genomes were more enriched in *aux* clusters on the megaplasmid. Although the single, ancestral T6SS was broadly conserved in the RSSC, the T6SS has been convergently lost in atypical, non-soilborne lineages. Overall, our data suggest dynamic interplay between the lifestyle of RSSC lineages and the evolution of T6SSes with robust arsenals of toxins. This pangenomic atlas poises the RSSC as an emerging, tractable model to understand the role of the T6SS in shaping pathogen populations.

**IMPORTANCE:**

We explored the eco-evolutionary dynamics that shape the inter-microbial warfare mechanisms of a globally significant plant pathogen, the *Ralstonia solanacearum* species complex. We discovered that most *Ralstonia* wilt pathogens have evolved extensive and diverse repertoires of type VI secretion system-associated antimicrobial toxins. These expansive toxin arsenals potentially enhance the ability of *Ralstonia* pathogens to invade plant microbiomes, enabling them to rapidly colonize and kill their host plants. We devised a classification system to categorize the *Ralstonia* toxins. Interestingly, many of the toxin gene clusters are encoded on mobile genetic elements, including prophages, which may be mutualistic symbionts that enhance the inter-microbial competitiveness of *Ralstonia* wilt pathogens. Moreover, our findings suggest that the convergent loss of this multi-gene trait contributes to genome reduction in two vector-transmitted lineages of *Ralstonia* pathogens. Our findings demonstrate that the interplay between microbial ecology and pathogen lifestyle shapes the evolution of a genetically complex antimicrobial weapon.

## INTRODUCTION

To defend their habitat or colonize new niches, host-associated bacteria attack competing microbes by secreting toxic molecules and proteins (1). Many Gram-negative bacteria use a molecular weapon known as the type VI secretion system (T6SS) to deliver toxic protein effectors to target cells (2, 3). T6SS toxins bind to the tip of a multimeric, spear-like projectile. Upon contracting, the sheath forcibly ejects the projectile, puncturing nearby cells and delivering the associated toxins. T6SS toxins kill target cells by degrading cellular components, such as DNA, lipid membranes, and bacterial cell wall polymers (4). To prevent damage to kin cells, each T6SS toxin has a cognate immunity protein that blocks the toxin’s toxicity.

Most T6SSes include a double-membrane-spanning complex (TssJLM) which recruits a baseplate complex (TssEFGK) and a spike complex (a VgrG trimer, a PAAR sharp tip, and toxins) (2). Hcp then polymerizes to form the shaft of the projectile, and repeating units of TssBC form a sheath around the Hcp shaft that contracts to fire the T6SS projectile and toxins. After firing, TssH disassembles the TssBC sheath, allowing the monomers to reassemble again in a new complex (5, 6). While just one copy of most *tss* genes is required for a functional T6SS, T6SS^+^ genomes usually encode multiple *vgrG* paralogs, usually co-located with a toxin/immunity gene pair (7). Multiple *vgrG* paralogs in a genome suggest the bacterial strain wields a diversity of toxins, potentially allowing the T6SS to be deadly against a diversity of targets. *VgrG* gene clusters may be located with the *tss* genes in the main T6SS locus or scattered around the genome as auxiliary T6SS gene clusters (*aux* clusters) (8).

Horizontal gene transfer (HGT) has previously been implicated in the spread of *aux* clusters within bacterial populations, contributing to convoluted evolutionary patterns of gain and loss that result in diverse T6SS toxin arsenals (9–12). Even closely related strains often have non-identical T6SS toxin repertoires, allowing interstrain competition within a bacterial species. For example, T6SS-mediated interstrain competition shapes competitive outcomes when *Vibrio fischeri* mutualists colonize their squid hosts (13). Moreover, large-scale analysis of all bacterial genomes in the IMG genome database reveals that host-associated bacteria, especially bacteria that colonize internal tissues of plants, are most likely to have a T6SS (7).

Plant pathogens in the *Ralstonia solanacearum* species complex (RSSC) have a functional T6SS (14, 15). Expression of T6SS genes is activated by the Phc quorum sensing system (16), which suggests that T6SS activity benefits RSSC pathogens when colonizing plant hosts. However, pinpointing the ecological role for the RSSC T6SS has been difficult because T6SS-inactive RSSC mutants have pleiotropic phenotypes, including altered motility and biofilm formation (14, 17).

Like many host-associated bacteria, RSSC plant pathogens must successfully transition between disparate ecosystems to complete their disease cycle. RSSC are soil-dwelling pathogens that chemotax through the microbially dense rhizosphere to infect the roots of a new host (18). After root entry, RSSC pathogens grow prolifically and clog the xylem (19), leading to wilt. Individual strains can infect diverse plants in multiple botanical families (20), and the RSSC collectively causes severe wilt disease on over 397 plant species (21), including economically important crops (22). As generalist plant pathogens with a free-living, rhizosphere-colonizing, and endosphere-colonizing lifestyle, RSSC pathogens likely compete with diverse microbes.

We hypothesized that the lifestyle of RSSC pathogens has shaped the evolution of their T6SS weapons. Here, we used a phylogenomic approach to shed light on the eco-evolutionary dynamics between the lifestyle of RSSC strains and their T6SS arsenals. We carried out a pangenome analysis for T6SS-related genes in RSSC genomes compared to genomes of Burkholderiaceae family relatives. We classified 25 types of RSSC *aux* clusters and investigated their phylogenetic distribution, location across the bipartite RSSC genomes, and identified mobile genetic elements (MGEs) that carry the clusters. Our analyses indicate that the T6SS is a dynamic weapon intimately linked to the evolutionary success of a species complex of soilborne plant pathogens of global concern.

## RESULTS

### Bacteria in the Burkholderiaceae family vary in their T6SS gene content

We used an evolutionary framework to explore T6SS gene content in the RSSC relative to their evolutionary neighbors in the Burkholderiaceae family. Using the genome taxonomy database (GTDB) (23), we identified complete-genome representatives of 289 Burkholderiaceae species (Tables S1 and S2). We used BLASTp to estimate the abundance of T6SS genes in the representative Burkholderiaceae genomes as well as a curated set of 99 high-quality RSSC genomes. To estimate the number of secretion systems per genome, we averaged the number of BLASTp hits for the core T6SS components: TssABCEFGHJKLM and Hcp. The majority of the Burkholderiaceae species representatives encoded at least one T6SS (62.3%) (Fig. S1; Table S1). A complementary approach using JackHMMER (24) produced similar results, indicating that 62.6% of genomes encoded at least one T6SS (Fig. S1; Table S1). While none of the 99 high-quality RSSC genomes had more than one set of T6SS genes, there were multiple T6SSes encoded in approximately 27% of the representative Burkholderiaceae genomes (Table S1). Three to six complete sets of T6SS core genes were identified in genomes in the *Burkholderia*, *Trinickia*, *Caballeronia*, *Variovorax*, *Pseudoduganella*, *Massilia*, and *Paraburkholderia* genera (Fig. S1; Table S1), which is consistent with previous reports for several of these taxa (25, 26).

### Genomes of RSSC plant pathogens are enriched in *vgrG* genes

**Fig 1:**
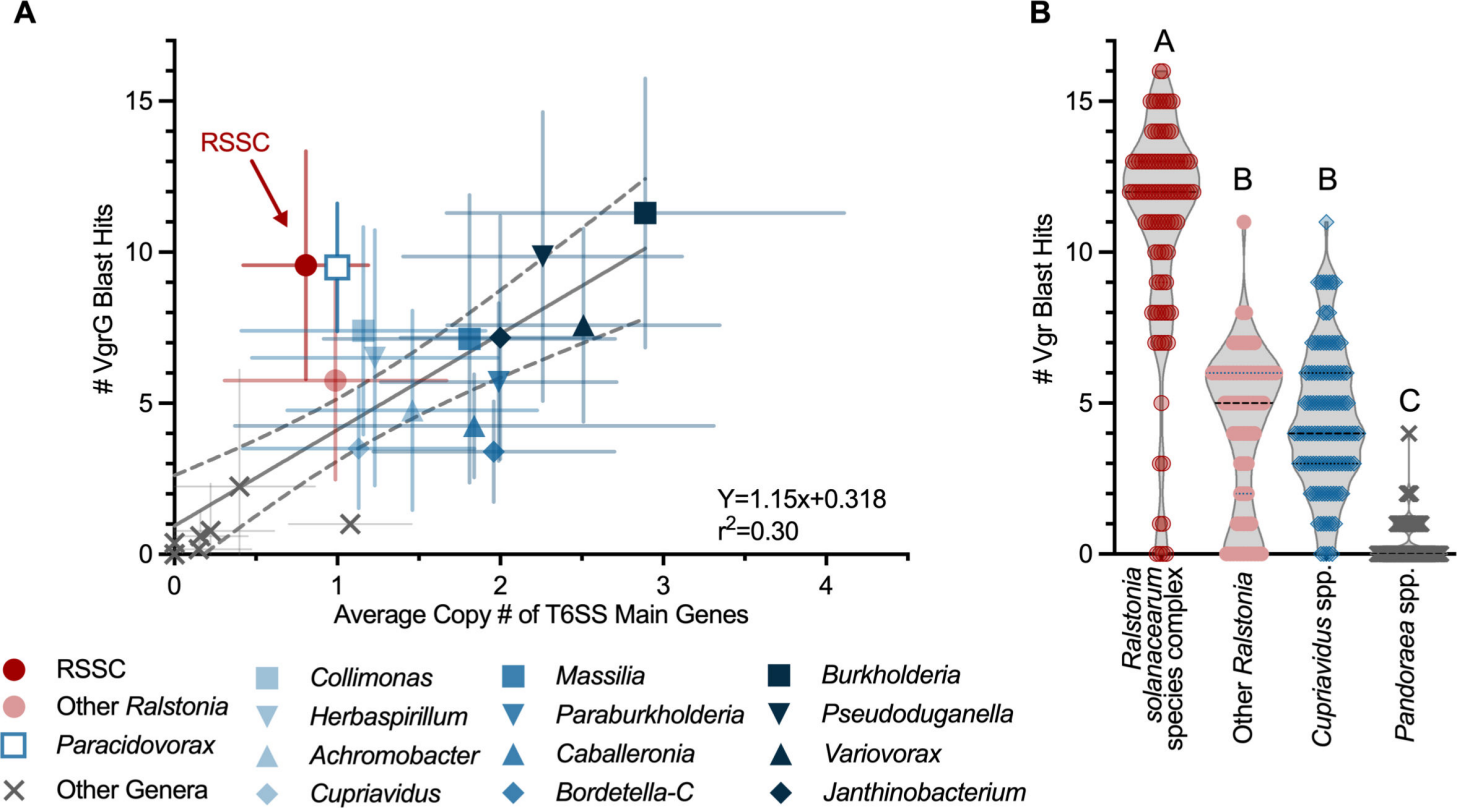
RSSC genomes are enriched in T6SS *vgrG* genes. (**A**) The number of VgrG homologs and T6SS core components (TssABCEFGHJKLM and Hcp) were compared across species within the Burkholderiaceae family. We identified T6SS core genes in a custom database of Burkholderiaceae genomes using BLASTp. The gray “X” symbols indicate the nine genera with few-to-no T6SS core genes [*Acidovorax sensu stricto* (30), *Alcaligenes*, *Bordetella*, *Comamonas*, *Hydrogenophaga*, *Pandoraea*, *Polaromonas*, *Polynucleobacter*, and *Rhodoferax*]. Error bars indicate standard deviation, and dashed lines indicate the 95% confidence bands around the linear regression. (**B**) The number of *vgrG* homologs in RSSC genomes (*n* = 99) relative to closely related taxa: other *Ralstonia* spp. (*n* = 70), *Cupriavidus* spp. (*n* = 120), and *Pandoraea* spp. (*n* = 75). VgrG homologs were identified using BLASTp. Letters indicate *P* < 0.0001 by the Kruskal-Wallis multiple comparisons test.

T6SS toxin/immunity pairs are often encoded in gene clusters with their corresponding *vgrG*, any adaptors that mediate toxin translocation, and genes encoding the PAAR sharpening tip protein (27–29). Because VgrG proteins have conserved sequences, we estimated the abundance of toxin/immunity pairs by searching genomes for VgrG homologs. We carried out multiple low-stringency BLASTp searches for VgrG homologs in the 289 Burkholderiaceae species representatives and in the 99 high-quality RSSC genomes. As expected, most of the genomes that lacked a T6SS also lacked *vgrG* genes (Fig. S2). Generally, there was a positive correlation between the copy number of T6SS core genes and *vgrG* homologs (Fig. 1A; Fig. S2).

Two taxa with only one T6SS were enriched in *vgrG* genes: the plant-pathogenic RSSC and the *Paracidovorax citrulli* species complex, which contains the causal agents of bacterial fruit blotch, *P. citrulli* and *Paracidovorax avenae* (Fig. 1A). *Paracidovorax* is a recently renamed genus that includes the xylem-infecting plant pathogen species previously known as *Acidovorax citrulli* and *Acidovorax avenae* (30), which are known to wield T6SSes (31). To investigate whether RSSC genomes are enriched in *vgrG* homologs, we compared the number of *vgrG* genes in the RSSC to other species in the *Ralstonia* genus (*n* = 70 genomes) and the closely related genera *Cupriavidus* (*n* = 120 genomes) and *Pandoraea* (*n* = 75 genomes). Plant-pathogenic RSSC genomes typically had over two times as many *vgrG* homologs as their close relatives (median of *n* = 12 per genome; Fig. 1B; Fig. S2A). Most *Pandoraea* spp. did not encode any *vgrG* homologs. *Cupriavidus* and the non-RSSC *Ralstonia* both encoded a small number of *vgrG* homologs (medians of *n* = 4 and *n* = 5 per genome, respectively) (Fig. 1B; Fig. S2A).

### A single T6SS subtype is largely conserved among plant-pathogenic RSSC

**Fig 2:**
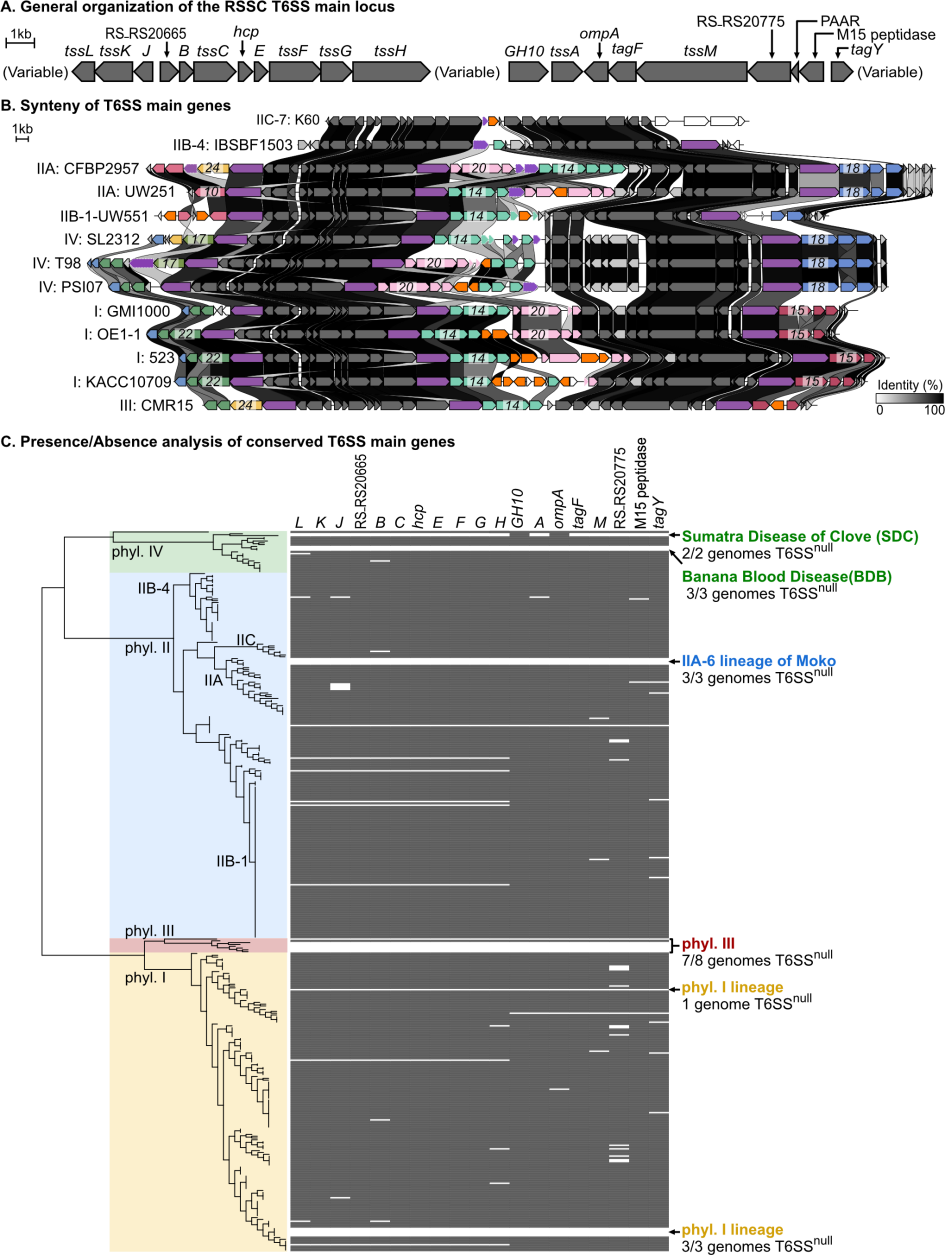
Although the T6SS is broadly conserved among RSSC strains, multiple lineages lack the T6SS. Most RSSC encode a T6SS^i4B2^ with a conserved gene order with three variable regions and two conserved regions. (**A**) shows the conserved genes and (**B**) displays the synteny of the locus across phylogenetically diverse RSSC strains. Core T6SS genes encoding structural components and associated genes are shown as gray arrows, VgrG spike protein-encoding genes as purple arrows, and IS elements and transposase genes as orange arrows. Other color-coded genes in the diagram belong to 1 of the 25 different *aux* types we identified (Fig. 3; Fig. S6 to S26), with the *aux* Numbers superimposed on the putative T6SS toxin. Numbers superimposed over genes in the variable regions identify which *aux* type is encoded at each spot. Linkages are drawn between homologous genes, with darker linkages indicating higher identity. Synteny and global amino acid identity were visualized with Clinker and aesthetics were adjusted in Affinity Designer. (**C**) Left: an approximate maximum likelihood phylogenetic tree of 398 RSSC genomes was created with the KBase Species Tree app. Right: the presence/absence of core T6SS genes based on BLASTp searches. Gray rectangles indicate at least one homolog was identified using BLASTp with percent identity cutoffs of >20% and aligned-length cutoffs of >80%. The data were visualized on iTOL. Lineages that lack the T6SS are indicated.

We used synteny analysis to investigate the organization of T6SS core genes among the plant-pathogenic RSSC. The genomes we investigated have the same T6SS subtype, T6SS^i4B2^ (32) (Fig. 2A), encoded on the ~2.1 Mb secondary replicon known as the megaplasmid (33). The RSSC T6SS main locus has two conserved regions located between three variable regions that contain *vgrG*-linked toxin/immunity clusters and transposable elements (Fig. 2A and B). We later defined these *vgrG-*linked auxiliary (*aux*) clusters as *aux10*, *aux14*, *aux15*, *aux17*, *aux18*, *aux20*, *aux22*, *aux23*, and *aux24*. Additionally, the phylotype IV strains have a five-gene insertion between *tssA* and *ompA* in the second conserved region (Fig. 2B). This five-gene insertion is also present in phylotype II strains at a different genomic location than the T6SS main locus, and has no known function or homology to T6SS genes, so its association with the T6SS is unclear (Fig. S3).

We identified 20 core genes in the RSSC T6SS loci (Fig. 2A). We BLASTp-searched 19 of the 20 core genes (excluding PAAR, which typically has multiple paralogs in T6SS^+^ genomes) against the 289 Burkholderiaceae species representatives. Although the T6SS structural genes were well conserved, we observed that six of the core RSSC genes were variably present in 21–71% of the Burkholderiaceae T6SS-containing genomes: RS_RS20665 (present in 57.7% of T6SS^+^ RSSC genomes), *GH10* (glycosyl hydrolase 10, in 21.4% of T6SS^+^ RSSC genomes), *ompA* (70.9%), *tagF* (62.6%), RS_RS20775 (30.2%)*,* and the M15 peptidase (59.9%, Fig. S1C). The *Ralstonia* T6SS includes a unique TagY-like protein that lacks the typical cysteine-rich C-terminal domain (34).

### The T6SS has been lost in several RSSC lineages

Upon searching 398 publicly available RSSC genomes, we identified several lineages that lacked the T6SS (T6SS^null^). The T6SS^null^ lineages include the two insect- and mechanical-vectored phylotype IV lineages that cause Sumatra Disease of Clove (SDC) and Blood Disease of Banana (BDB) (35), one of several lineages that causes Moko Disease of Banana (phylotype IIA-6) (36), one phylotype I lineage, and all but one of the eight phylotype III strains with sequenced genomes (Fig. 2C). Of these T6SS^null^ lineages, the SDC lineage genomes encode the putative glycosyl hydrolase (GH10) and *ompA* genes, suggesting that the other T6SS genes were lost. We also identified multiple genomes that lacked the conserved *tssL*-to-*tssH* and the *GH10*-to-*tagY-like* regions, *n* = 30 and *n* = 24, respectively (Fig. 2C; Table S2). There were sporadic genomes that lacked a BLASTp hit for certain core genes, but these could be false negatives from genome assembly/annotation errors.

To infer whether the T6SS^i4B2^ had been lost in the T6SS^null^ lineages, we carried out BLASTp searches and synteny analysis with all the T6SS gene clusters identified in RSSC and the other *Ralstonia* species. Of the T6SS^+^ non-RSSC *Ralstonia*, 98% of the genomes encoded a T6SS^i4B2^ with the same genetic organization except for the absence of the GH10-domain gene (Fig. S4). Some genomes of non-RSSC *Ralstonia* spp. contained a second T6SS locus that did not match our reference (3), so we used synteny analysis to classify these into informal groups (Fig. S4; “Other A” and “Other B”). The simplest explanation for the phylogenetic pattern is that the T6SS^i4B2^ is ancestral to the genus *Ralstonia* and has been lost in multiple lineages of the RSSC and non-RSSC *Ralstonia*.

### The RSSC pangenome encodes dozens of auxiliary T6SS toxin/immunity clusters

Because RSSC genomes contain a median of 12 *vgrG* paralogs and only three *vgrG* paralogs are encoded in the main T6SS^i4B2^ locus, we hypothesized that there were additional T6SS loci on the chromosome or megaplasmid. We manually curated a list of 1,066 T6SS auxiliary toxin/immunity (*aux*) clusters in the 99 high-quality RSSC genomes using a process that combined low-stringency BLASTp searches with iterative synteny analysis of candidate *aux* clusters (see Materials and Methods). Based on shared genetic architecture, we classified 1,060 of the T6SS *aux* clusters into 25 different types, named *aux1–aux25* (Fig. S5; Table S3). The remaining six were not categorized because they consisted of only an orphan *vgrG* gene. We used synteny analysis to map the *aux* clusters to their locations on the chromosome, megaplasmid, or small accessory plasmids. As we predicted, a majority of *aux* clusters (*n* = 788, 73.9%) were located elsewhere in the genome from the main T6SS cluster. We illustrated the phylogenetic distribution, genomic loci, representative structural variants, and gene organization and annotations for each *aux* cluster type and compiled this data to create a pangenomic atlas of the T6SS in the RSSC (Fig. S6 to S26). Toxin arsenals of other *Ralstonia* genomes can be classified using synteny analysis with Clinker (37) using the reference genbank (.gbk) files for *aux1–aux25* (FigShare repository: https://doi.org/10.6084/m9.figshare.23065583.v1).

The *aux* clusters contain between 3 and 14 genes and vary in size from 2.3 kb (*aux17*) to 16.6 kb (*aux16*). We used the NCBI Conserved Domain Database [CDD (38)] and PaperBlast (39) to infer the function of each *aux* gene (Fig. S6 to S26). Although there is a conserved small gene that encodes a standalone PAAR domain protein in the T6SS main locus, small PAAR domain-containing genes were also encoded in some or all structural variants of five *aux* types (*aux1*, *aux2*, *aux5*, *aux7*, and *aux9*). Although some T6SS toxins bind directly to VgrG spike proteins, others form complexes with adaptor proteins that bind to the VgrG proteins. We identified adaptors with DUF1795 (40) and DUF2169 (41) domains in three *aux* types, each, and DUF4123 domains (42) in four *aux* types. Several of the *aux* clusters include genes containing polymorphic toxins with recombination hotspot (RHS) domains (*aux3*, *aux4*, and *aux12*), DUF4150 PAAR-like superfamily domains (*aux8*, *aux13*, and *aux16*) (43), marker-for-type-six (MIX_III) domains (*aux14* and *aux20*) (44), found-in-type-six (FIX) domains (*aux10*, *aux17*, *aux22*, *aux23*, and *aux24*) (45), and FIX-like domains (*aux19* and *aux21*). Finally, genes also included other domains that have been previously identified in T6SS toxins (DUF3274, DUF2235, and DUF6531) (46–48) and immunity proteins (DUF1910, DUF1911, DUF3304, and Sel1 repeats) (8, 45, 46) (Fig. S6 to S26). We identified putative arrays of immunity genes or orphan immunity genes in structural variants of thirteen *aux* types: *aux2*, *aux3*, *aux4*, *aux5*, *aux6*, *aux8*, *aux11*, *aux12*, *aux14*, *aux15*, *aux18*, *aux22*, and *aux23*.

The largest two clusters, *aux8* and *aux16*, are atypical in that they contained genes upstream of the *vgrG* gene: one DUF4124 gene and one-to-two ankyrin-repeat genes (Fig. S13 and S21). All genes in *aux1–aux24* were arranged unidirectionally, but *aux25* had an atypical three-gene layout of *vgrG*, a 2.3-kb hypothetical gene, and an inverted gene encoding a PvdO family nonheme iron enzyme (Fig. S26).

### T6SS *aux* cluster content varies among RSSC clades

The copy number of *aux* types ranged between zero and three copies among the RSSC genomes (Fig. S28). Some *aux* types were never found with more than a single copy per genome. The *aux2*, *aux5*, and *aux7* clusters were sometimes found in two or three copies, and *aux1*, *aux4*, *aux6, aux9*, and *aux17* were occasionally found in two copies across the RSSC genomes. The *aux1*, *aux*2, *aux3*, *aux5*, *aux6, aux7*, *aux14*, and *aux15* clusters were found in more than 50% of the RSSC genomes. In contrast, *aux10*, *aux11*, *aux12*, *aux18*, *aux19, aux21*, *aux24*, and *aux25* were found in less than 20% of the RSSC genomes (Fig. S28).

**Fig 3:**
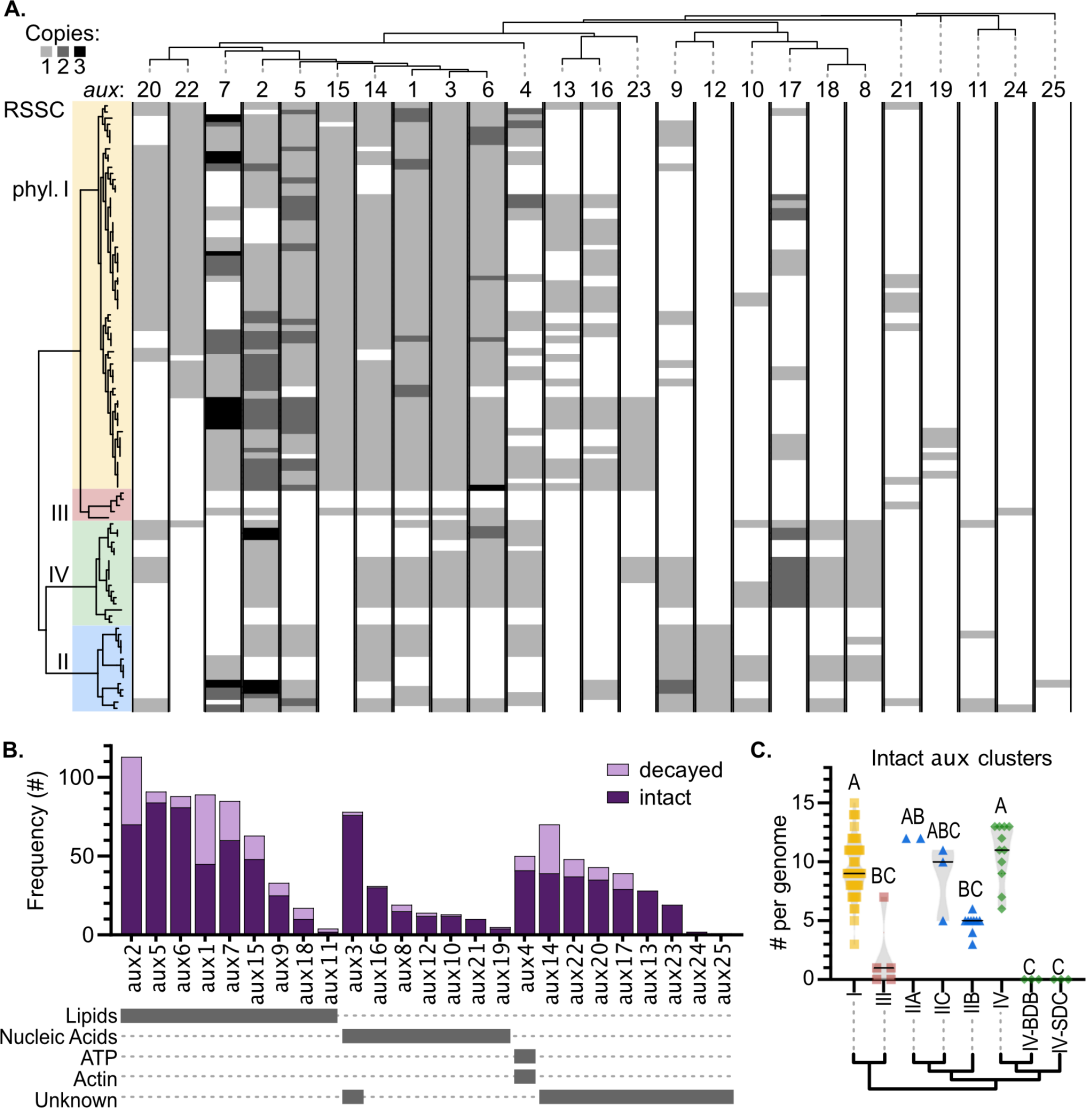
RSSC strains vary in their repertoires of *vgrG*-linked auxiliary toxin/immunity clusters (*aux*). We classified 1066 *vgrG*-linked clusters into 25 subtypes (*aux1–aux25*). (**A**) Abundance of each *aux* cluster (*aux1–aux25*) across complete or nearly complete RSSC genomes; both decayed and intact *aux* clusters are shown. To see the distribution of only the intact or decayed *aux* clusters, see Fig. S28. The left dendrogram is the same RSSC phylogeny displayed in Fig. 3D. The top dendrogram groups *aux* by their phylogenetic distribution in the RSSC genomes. (**B**) Clusters were classified as decayed if the *vgrG*, toxin, or immunity genes were pseudogenized or disrupted by transposons and were classified as intact if they lacked obvious mutations. (**C**) Abundance of intact *aux* clusters across major phylogenetic divisions of the RSSC, with black lines indicating the median. Letters indicate significance by the Kruskal-Wallis test with multiple comparisons (*P* < 0.05).

To compare the phylogenetic distribution of different *aux* types, we visualized the copy number of each *aux* type across a species tree (Fig. 3A; Fig. S28). Using the abundance of *aux* types in our genome set, we created a dendrogram that hierarchically clusters *aux* types based on their prevalence across the RSSC genomes (Fig. 3A). The *aux1*, *aux2*, *aux3*, *aux6*, and *aux14* clusters were present in at least one genome of all four phylotypes, excluding the T6SS^null^ phylotype III strains. In contrast, some *aux* types were restricted to specific phylotypes: *aux12* and *aux25* were present only in phylotype II, while *aux13* and *aux19* were only found in phylotype I. *Aux11*, *aux19*, *aux24*, and *aux25* clustered closely together due to their rare presence in a few RSSC genomes (Fig. 3A; Table S3). As a result of these highly variable patterns of *aux* cluster distribution, closely related strains typically have overlapping but non-identical *aux* cluster repertoires (Fig. 3A).

### The putative T6SS toxins target diverse substrates, including lipids and DNA

**Fig 4:**
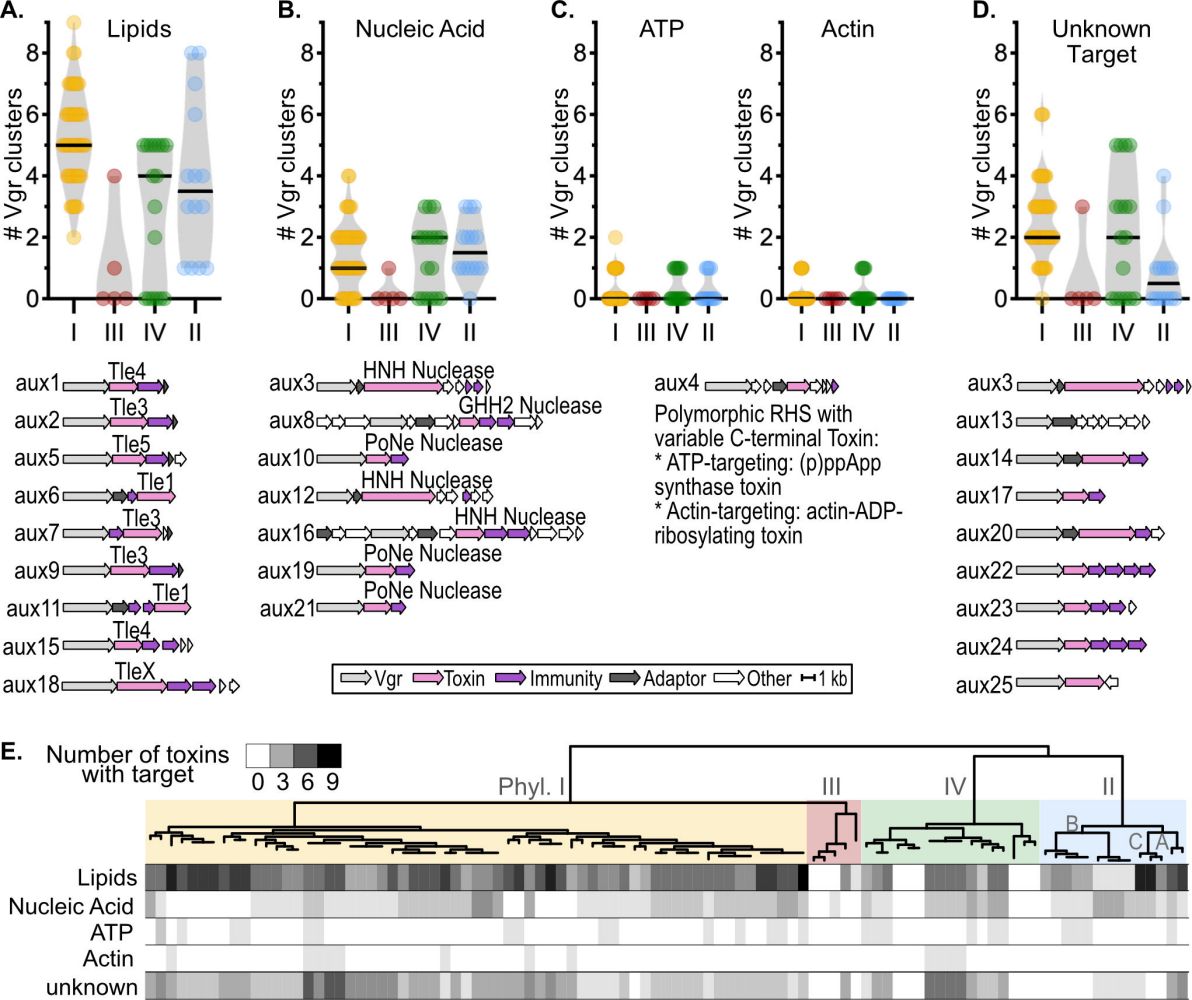
RSSC VgrG-linked toxins are predicted to target lipids, nucleic acids, ATP, actin, and unknown targets. To identify toxins and analyze their sequence for enzymatic domains, all *aux* cluster amino acid sequences were queried against NCBI Conserved Domain Database (CDD) and PaperBlast. (**A–D**) Comparison of the abundance of *aux* clusters with (**B**) lipase, (**C**) nuclease, (**D**) ATP-degrading or Actin-targeting domains, or (**E**) unidentified functional domains across the four phylotypes (I–IV). Below each graph is a cartoon of the genetic architecture of a representative of each *aux* type. (**E**) Phylogenetic analysis of the toxin profiles. Top: an approximate maximum likelihood phylogenetic tree of 99 high-quality RSSC genomes was created with the KBase “Species Tree” app. Below: a heat map of each genome’s toxin repertoire. Table S3 lists the specific repertoire of each genome.

Most T6SS toxins damage important cellular components in target cells. We used the NCBI CDD and PaperBlast to infer the mode of action of the toxins (Fig. 4; Fig. S27; Table S3). The toxins from nine *aux* clusters were lipases of previously defined families (49): Tle1 (*aux6* and *aux11*), Tle3 (*aux2*, *aux7*, and *aux9*), Tle4 (*aux1* and *aux15*), Tle5 (*aux6*), and a lipase with a novel domain architecture (*aux18*). The toxins of seven *aux* clusters were nucleases with HNH nuclease domains (*aux3*, *aux12*, and *aux16*), GHH2 nuclease domains (*aux8*), or PoNe nuclease domains (*aux10*, *aux19*, and *aux21*). The RHS toxins in *aux4* have variable C-terminal toxin domains predicted to target either ATP as (p)ppApp synthase or actin as actin-ADP ribosylase. We could infer which gene was the toxin based on the presence of known polymorphic toxin domains for eight of the clusters (*aux13*, *aux14*, *aux17*, *aux20*, *aux22*, *aux23*, *aux24,* and *aux25)*, but we could not identify domains or motifs that hint at the mode of action (Fig. 4D). For the remaining cluster, *aux13*, the toxin might be either the MAEBL-domain or one of the three hypothetical proteins.

We investigated whether RSSC lineages varied in the substrates targeted by their T6SS toxins (Fig. 4). All T6SS^+^ RSSC genomes encoded at least one lipase. Phylotype I and IIC lineages encoded the most lipases (median of 5 and 8, respectively) (Fig. 4A through E). Phylotype IIB-1, which contains the clonal pandemic lineage [regulated as a U.S. Select Agent under the name “*R. solanacearum* R3Bv2” (50)], had the fewest lipases of the T6SS^+^ genomes with only a single lipase per genome. Nucleases were common, but 17% of T6SS^+^ strains lacked any obvious nucleases. The IIB-1 lineage, the IV-8 lineage, and scattered phylotype I strains had more nucleases than other lineages (Fig. 4E). Genomes with more nucleases tended to have fewer lipases. The putative ATP and actin-targeting toxins were rare and sporadically distributed. Most of the T6SS^+^ genomes (91%) had one or more toxins with unknown targets.

### *aux* clusters are enriched on the RSSC megaplasmid

**Fig 5:**
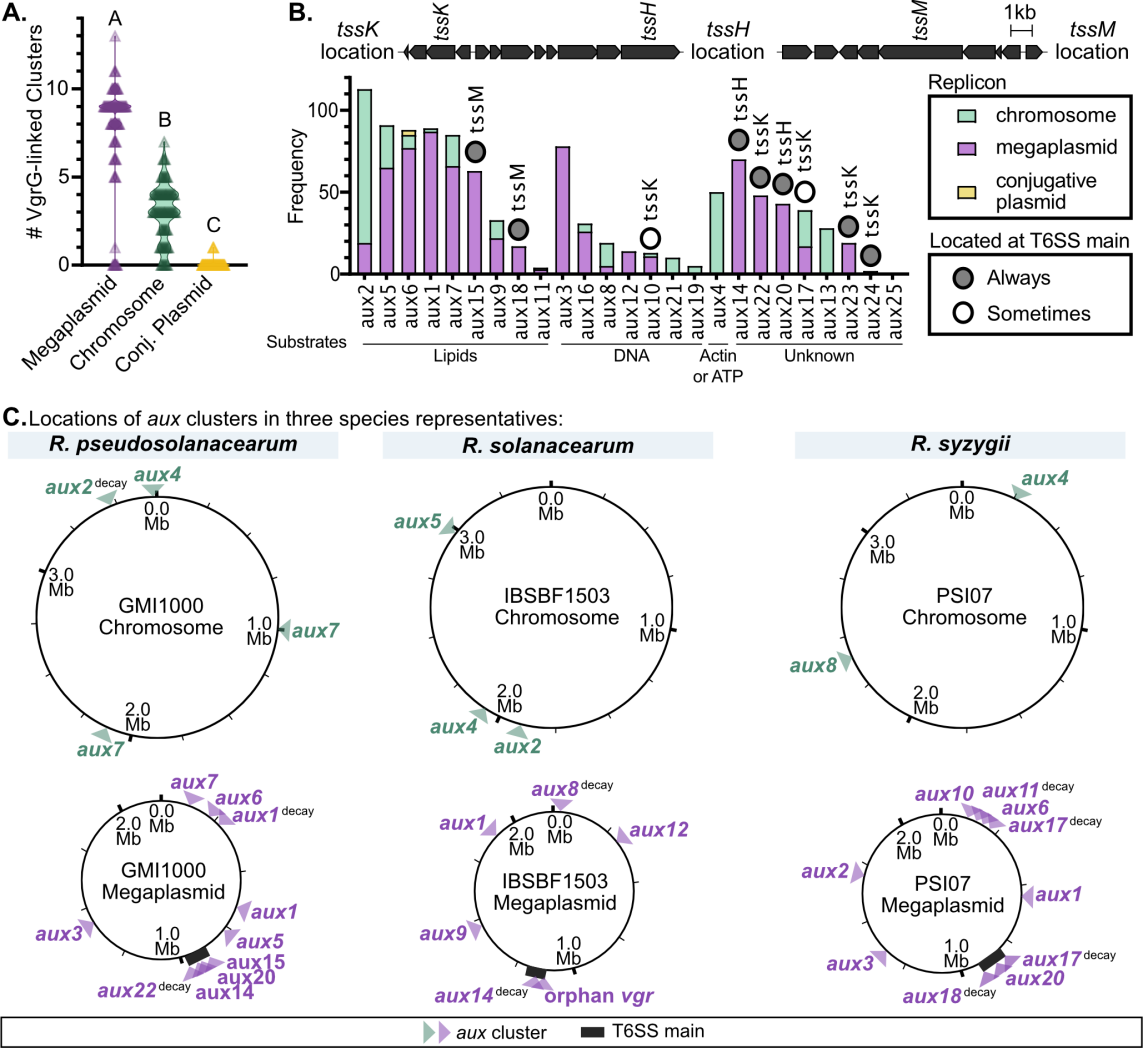
*VgrG*-linked toxins/immunity clusters are enriched on the megaplasmid. (**A**) The number of *aux* clusters on each replicon (the chromosome, megaplasmid, and accessory conjugative plasmids) per RSSC genome. Letters indicate *P* < 0.0001 by Brown-Forsythe and Welch ANOVA with Dunnett’s T3 multiple comparisons test. (**B**) The occurrence of each *aux* cluster type on the chromosome, megaplasmid, or accessory plasmids. The *aux* clusters are sorted by the substrate. Nine *aux* clusters were always (dark gray circles) or sometimes (white circles) located in the T6SS main locus. The three locations where *aux* clusters are found in the T6SS main locus are indicated above the graph. (**C**) The locations of *aux* clusters on the chromosome and megaplasmid of RSSC species representatives (*R. pseudosolanacearum* GMI1000, *R. solanacearum* IBSBF1503, and *R. syzygii* PSI07). Like most RSSC genomes, these lack accessory conjugative plasmids.

In bacterial genomes, secondary replicons like the RSSC megaplasmid often contain more rapidly evolving accessory genes than the chromosome (51–53). Because *aux* clusters are part of the RSSC accessory genome, we hypothesized that they would be more abundant on the megaplasmid. We found that *aux* clusters were enriched on the megaplasmid, accounting for 72% of the 1,066 *aux* clusters that we classified (Fig. 5; Fig. S29; Table S3). Considering that the megaplasmid is smaller than the chromosome (approximately 2.1 and 3.5 Mb, respectively), *aux* cluster density is dramatically higher on the megaplasmid. Nevertheless, certain *aux* types were more common on the chromosome: *aux2*, *aux8*, *aux21*, *aux19*, *aux4*, *aux17*, and *aux13* (Fig. 5B; Table S3).

To determine whether there are genomic islands where *aux* clusters are frequently located, we used synteny analysis (37) to map the specific location of each of the 1,066 *aux* clusters. Except for the T6SS main locus, there were no obvious hotspots where *aux* clusters were located (Fig. 5C and the “C panels” of Fig. S6 to S26). The three variable regions of the T6SS main locus each contained *aux* clusters that had the same superfamily of toxins. The variable region upstream of *tssM* only contained *aux15* or *aux18*, which had lipase toxins (Fig. S20). The variable region between *tssH* and *tssA* exclusively contained *aux14* and/or *aux20*, which have a MIX_III domain toxin (Fig. S19). All *aux* clusters found downstream of *tssJKL* contained toxins with FIX domains, including *aux22–aux24*, which were exclusively found at T6SS main locus, and *aux10* and *aux17*, which were also found associated with the T6SS main locus as well as additional loci (Fig. S15, S22 and S25). Certain *aux* types were located in consistent genomic loci, while others were highly variable. For instance, *aux3* was found in 79% of surveyed RSSC genomes and was always found in the same locus on the megaplasmid (Fig. S8). In contrast, *aux2* was found in 84% of our genome set and is located in nine distinct loci (Fig. S7). Some of our RSSC genomes included accessory plasmids that encoded two different *aux* types, *aux6* and *aux11*. However, both of these *aux* types were also found on the chromosome or megaplasmid in other RSSC genomes (Fig. S11 and S16).

### Loss-of-function mutations are common among *aux* clusters

To infer the role of gene loss in the evolutionary history of RSSC *aux* clusters, we used synteny analysis to identify apparently functional “intact” *aux* clusters and “decayed” clusters with one or more putative loss-of-function mutations (Fig. S30). Loss-of-function mutations included deletions, frameshifts, premature stop codons, or gene disruption by insertion sequence (IS) and other transposable elements (Table S3). Of the 1,066 *aux* clusters classified, about 23.5% contained one or more *vgrG*, toxin, or immunity genes that had one of these loss-of-function mutations (Table S3). Of the 251 decayed *aux* clusters, 70.5% had mutations in *vgrG*, 34.7% had mutated toxins, and 17.5% had mutated immunity genes. Only 8.8% of the decayed *aux* clusters had mutations in the immunity gene but not the toxin or *vgrG* genes. Some *aux* types were more frequently decayed than others (Fig. 3B; Fig. S28; Table S3). The least commonly decayed *aux* clusters were those with nuclease toxins (*aux3*, *aux8*, *aux10*, *aux12*, *aux16*, *aux19*, and *aux21*) as well as *aux13* and *aux23*, which encode toxins with unknown targets. The most commonly decayed *aux* clusters were *aux1* (43/88 decayed), *aux2* (42/115 decayed), and *aux14* (36/75 decayed).

Most T6SS^+^ RSSC genomes have at least one decayed *aux* cluster. We investigated whether RSSC lineages varied in their proportion of decayed *aux* clusters which could suggest that these lineages had less ecological pressure to maintain large toxin repertoires (Fig. S31). As expected, the T6SS^null^ lineages (III, IV-BDB, and IV-SDC) mostly lack intact *aux* clusters, although the IV-BDB genomes had decayed *aux* clusters, and two T6SS^null^ phylotype III strains had intact *aux6* and *aux21* clusters (Fig. S28; Table S3). Although phylotype IIB is T6SS^+^, IIB genomes encode fewer intact *aux* clusters than other T6SS^+^ clades, with a median of five intact *aux* clusters per genome (Fig. 3A through C). A small clade within phylotype I also had five decayed *aux* clusters per genome (Fig. 3; Fig. S28). Within the sample of genomes analyzed, there is no particularly strong phylogenetic pattern to the prevalence of decayed *aux* clusters.

### Mobile genetic elements facilitate horizontal acquisition of chromosomal *aux* clusters

The phylogenetic distribution of *aux* clusters among RSSC genomes suggests a complicated pattern of gene flow with frequent gain events in addition to the loss events documented above. We hypothesized that HGT between RSSC clades may contribute to the convoluted phylogenetic pattern of *aux* cluster presence and absence. In bacteria, mobile genetic elements like phages and conjugative plasmids are common vehicles for the horizontal transmission of genes (54). We used a combination of bioinformatic analyses to investigate if MGEs were associated with *aux* clusters in RSSC genomes, including synteny analysis with Clinker (37), prophage prediction with PHASTER (55), and domain analysis with NCBI CDD (38).

**Fig 6:**
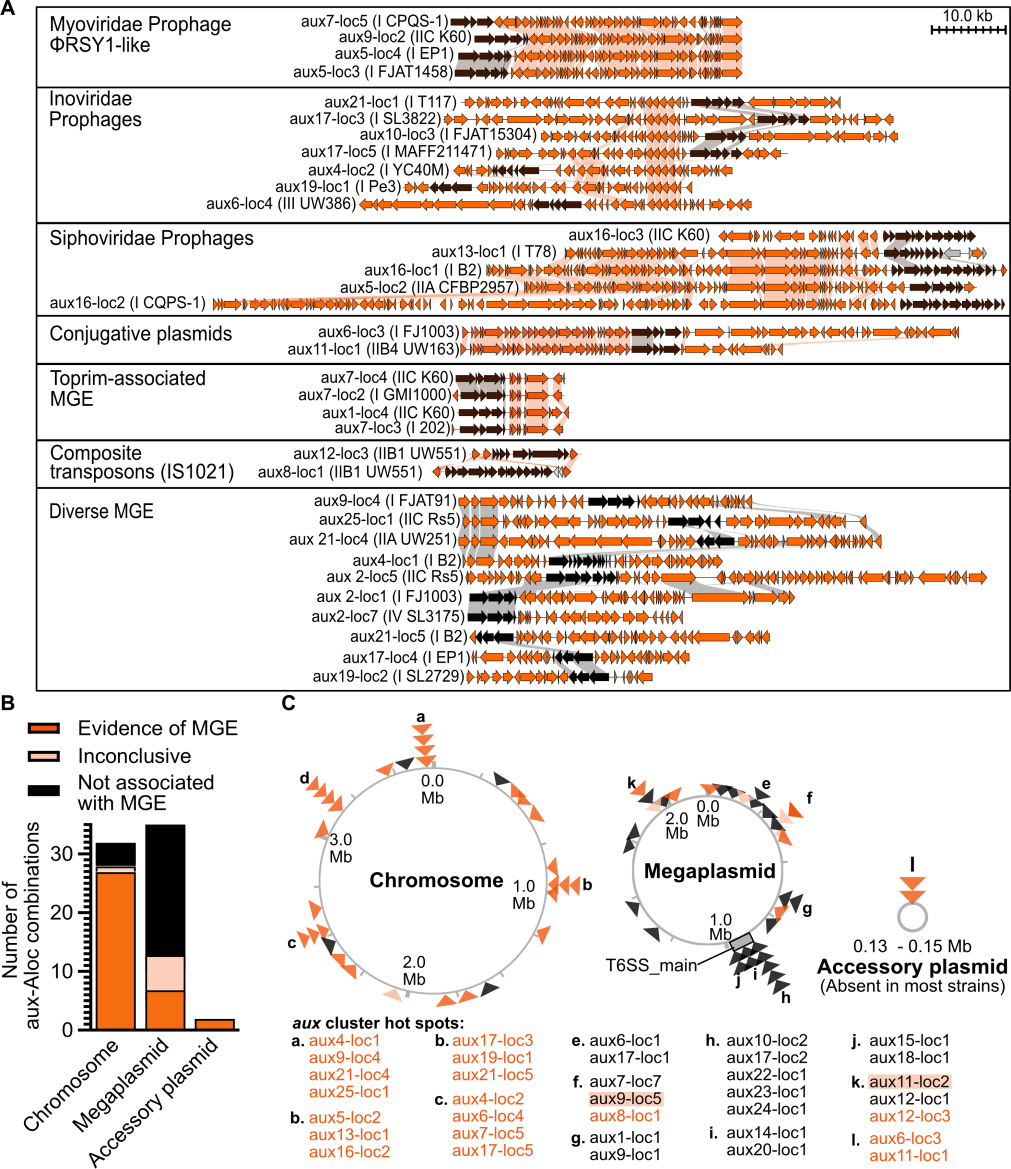
Mobile genetic elements likely mediate horizontal gene transfer of *aux* clusters. (**A**) Synteny analysis of representative *aux* clusters associated with MGEs. MGE genes are orange, *aux* cluster genes are brown, and all other genes are gray. (**B**) Proportion of *aux* clusters on the chromosomes, megaplasmids, and accessory plasmids that are MGE-associated, inconclusive, or not MGE-associated. (**C**) Specific locations of each *aux*-locus across the RSSC genomes. Each triangle represents one *aux*-locus, and colors correspond to the legend in (**B**). The letters identify hotspots where multiple *aux*-loci were found across the RSSC pangenome. Locations of *aux* clusters were identified using the GMI1000 genome as reference.

Of the 72 unique genetic neighborhoods around *aux* clusters, we classified 50% as MGE-associated and 40.3% as not MGE-associated. We assigned the remaining 9.7% of clusters “inconclusive” status because there was minor but insufficient evidence of association with an MGE. For example, one inconclusive cluster was adjacent to a single pseudogenized phage portal gene. Many *aux* clusters from the IIB-1 pandemic brown rot lineage were associated with IS1021 elements (Fig. S32). Two of these IIB-1 *aux* clusters, *aux8* and *aux12*, are flanked by IS1021 elements, suggesting that these are composite transposons. The IIB-1 *aux* clusters with only one IS1021 element were assigned to the “inconclusive” group. In total, we identified prophages (Myoviridae, Inoviridae, and Siphoviridae families), conjugative plasmids, composite transposons, and 16 other unclassified MGEs that were co-inherited with *aux* clusters (Fig. 6A; Fig. S32; Table S3). Of the prophages, the Myoviridae and Siphoviridae phages had high conservation in gene structure, whereas the filamentous Inoviridae phages were highly diverse.

Many of the MGEs were associated with lipase *aux* types. The ϕRSY1-like Myoviridae prophages (56) carried certain lipase clusters: *aux5*, *aux7*, and *aux9* at three, one, and two genomic locations, respectively (*aux5^loc3,4,7^*, *aux7^loc5^*, and *aux9^loc1,2^*) (Fig. 6A; Fig. S33). *Siphoviridae* prophages carried a lipase cluster and two clusters with unknown targets: *aux5*, *aux13*, and *aux16* at three locations (Table S3). Inoviridae prophages carried clusters with diverse toxins: *aux4*, *aux6*, *aux10*, *aux17*, *aux19*, and *aux21* (Fig. 6). An unclassified eight-gene MGE with a Toprim topoisomerase carried two lipase clusters *aux1* and *aux7* at three locations (Fig. 6A; Fig. S34; Table S3). Finally, conjugative plasmids carried two lipase clusters: *aux6* and *aux11* (Fig. 6A; Fig. S33).

We investigated the genomic locations of MGE-associated, inconclusive, and non-MGE *aux* clusters. Surprisingly, we found that there was a strong linkage between chromosomal location and MGE-association (Fig. 6B). Whereas 84% of chromosomal *aux* clusters were MGE-associated, only 18% of megaplasmid *aux* clusters were MGE-associated. All accessory plasmids with *aux* clusters were conjugative plasmid MGEs. We identified several *aux*-carrying MGE hotspots on the chromosome, most of which were dominated by a single prophage family (Fig. 6C). For example, hotspot B was dominated by the Siphoviridae phages, while hotspots C and D were both dominated by Inoviridae prophages. In contrast, hotspot A was occupied by four distinct unclassified MGEs (Fig. 6C).

## DISCUSSION

Here, we used a phylogenomic approach to shed light on the eco-evolutionary dynamics between the lifestyle of RSSC strains and their T6SS arsenals. We infer that the T6SS is an ancestral trait in the RSSC. We found that RSSC genomes are evolutionarily enriched in T6SS toxins. Scrutinizing the diversity and distribution of T6SS toxins in the RSSC, we found a complex distribution of T6SS *aux* clusters suggesting that *aux* clusters are frequently gained and lost. Additionally, we found that the T6SS is more prevalent across the RSSC (95.2% of our 398-genome RSSC data set) compared to close relatives in the Burkholderiaceae family (62.4% of our 289-genome Burkholderiaceae data set). Notably, many of the RSSC lineages that have lost the T6SS are capable of mechanical- or insect-vectored transmission and are not exclusively soilborne. Our analyses suggest that the T6SS is a dynamic weapon intimately linked to the evolutionary success of soilborne RSSC, a group of plant pathogens of global concern.

Bacteria in the RSSC are aggressive pathogens that cause lethal wilt disease in plants, resulting in drastic losses of economically important crops. RSSC pathogens are renowned for manipulating a broad range of host plants with massive repertoires of 60–80 type III secreted toxins (57). Here, we reveal that RSSC strains have also evolved large arsenals of T6SS toxins. The higher prevalence of T6SSes and expansion of T6SS toxin arsenals in RSSC genomes compared to other Burkholderiaceae lineages suggest that possessing a large T6SS arsenal is adaptive for the lifestyle of these pathogens. Consistent with this hypothesis, the Phc quorum sensing system that controls many RSSC virulence factors, including the production of EPS and the T3SS, also induces expression of many T6SS core genes and *vgrG* paralogs when the model strain GMI1000 grows *in planta* (16). During transmission and infection of a new host, they encounter a variety of ecological interactors. We speculate that RSSC bacteria share the indiscriminate aggression of a pack of hyenas. Similar to how hyenas attack other animals with their teeth and claws, RSSC bacteria may wield their T6SS against competitors (other RSSC and plant-colonizing bacteria), predators (bacterivorous amoebae) and, possibly, their prey (plant hosts). Nevertheless, we speculate that bacterial competitors are the main target for the RSSC T6SS, but future studies are needed.

Our data support the model that there is eco-evolutionary feedback between bacterial lifestyle and enrichment of T6SS-related genes (58). We discovered that the xylem-pathogenic RSSC and *Paracidovorax* spp., two taxa of ecologically similar plant pathogens, have convergently evolved to deploy a large array of toxins from a single T6SS. These pathogens both cause acute infections of the plant xylem (30). In contrast, plant mutualistic *Rhizobia* and pathogenic *Agrobacterium* encode fewer toxins (9), suggesting that the lifestyle of these long-term colonists selects against diverse arrays of T6SS toxins that could cause collateral damage to the host. Certain Burkholderiaceae encode numerous distinct T6SSes in their genome, which is consistent with a model that organisms with complex lifestyles benefit from having multiple T6SSes that are independently regulated and specialized for different targets and contexts (25). Organisms with a single T6SS like the RSSC likely choose to fight in a single ecological arena.

The relationship between bacterial lifestyle and T6SS gene content is also evident within the RSSC. Most RSSC pathogens are transmitted through soil or surface water (59). However, two of the phylotype IV clades that lack a T6SS have adopted novel lifestyles: the strains causing SDC and BDB (60, 61). SDC strains are spread by piercing-sucking insects (62), while BDB strains are mechanically transmitted by insects or agricultural tools (35, 63). Like many bacteria that transition to a host-restricted lifestyle, these lineages have undergone a reduction in genome size. While SDC and BDB strains have chromosomes of comparable size to soil-transmitted relatives, their megaplasmids are reduced in size by approximately 200 kb (10%) and 400 kb (20%), respectively (64, 65). While the two SDC-lineage genomes lacked *aux* clusters, they encoded 2 of the 20 core T6SS genes (*GH10* and *ompA*). Similarly, all three genomes in the BDB lineage lacked the core T6SS genes but contained decayed *aux* clusters. Overall, this pattern suggests that the T6SS is ancestral to the RSSC and has been recently lost in the genome-reduced *R. syzygii* lineages.

Pioneering studies on Moko disease by Luis Sequeira and Ivan Buddenhagan demonstrated that the causal phylotype II RSSC strains are facultatively transmitted either by infested soil or mechanically by insect contact with sap, similar to BDB (66, 67). We and other groups have sequenced isolates from the Sequeira and Buddenhagan collection from the 1960s Moko epidemic and recent isolates (20, 36, 68, 69), and we now know that multiple RSSC lineages were responsible for the epidemic, including T6SS^+^ lineages (IIB-4 and IIB-3) and T6SS^null^ lineages (IIA-6). Further research is needed to understand how variation in transmission routes shapes the evolution and behavior of these and other banana-infecting RSSC lineages (70).

Although the T6SS is widely conserved in soil-transmitted RSSC, the absence of the T6SS in several soil-transmitted lineages demonstrates that the T6SS is nonessential for this lifestyle. Soil-transmitted RSSC that lack a T6SS included all but one of the phylotype III genomes and a minor clade of phylotype I (Fig. 2C). Three of the T6SS^null^ phylotype III strains had intact *aux* clusters, which could suggest that the T6SS was recently lost from this lineage. However, two of the three intact *aux* clusters were associated with MGEs, so an alternative hypothesis is that these *aux* clusters were recently gained through HGT after a more ancient loss of the T6SS. Our results provide new insight into the epidemiology of RSSC in regions with multiple lineages. It has long been known that RSSC strains can inhibit each other’s growth and competitively exclude each other *in planta* (71, 72). A thorough epidemiological survey of Malagasy vegetable plots demonstrated that T6SS^+^ phylotype I strains are displacing the T6SS^null^ phylotype III strains native to the island (73). Subsequent functional analysis demonstrated that the phylotype I Malagasy strains secrete bacteriocin toxins into culture supernatant that inhibit the growth of the phylotype III strains (74). Our results suggest that the T6SS may confer an additional advantage to phylotype I strains when they directly compete against T6SS^null^ phylotype III strains.

The convoluted phylogenetic distribution of *aux* clusters suggests that RSSC populations have dynamic T6SS gene flow with frequent gain and loss events. Although there is evidence that certain *aux* types could have been primarily vertically inherited, most *aux* clusters are clearly horizontally transferred based on their phylogenetic distribution and the diversity of their genetic neighborhoods. Notably, the ϕRSY1-like Myoviridae prophages definitively transfer *aux* clusters. ϕRSY1 was originally isolated from soil from an *R. pseudosolanacearum*-infested field, and whole genome sequencing of the purified virion particles confirms that the genome of ϕRSY1-like phages encode *aux* clusters and, thus, do transmit *aux* clusters (56). The presence of *aux* clusters in phage genomes indicates that these phages may function as mutualists of the RSSC. Across the diversity of RSSC-infecting phages, most do not transport *aux* clusters (75–78). Nevertheless, with growing interest in employing phages for control of bacterial plant pathogens (75, 79–83), it will remain important to evaluate candidate biocontrol agents for their ability to improve the ecological fitness of the targeted pathogens.

Like many bacteria in the Burkholderiaceae, RSSC have a bipartite genome. When bacteria have a secondary replicon like the megaplasmid, the genes on the secondary replicon usually evolve more quickly than chromosomal genes (51–53). Intriguingly, we discovered that *aux* clusters are dramatically enriched on the megaplasmid. We speculate that the enrichment of *aux* clusters on the evolutionarily dynamic megaplasmid could allow RSSC to rapidly diversify their toxin arsenals. In-depth studies on the molecular evolution of chromosomal and megaplasmid *aux* cluster genes are needed to determine if they evolve at different rates from each other or from other RSSC genes.

In the bipartite RSSC genomes, we found clear distinctions in the mechanisms for horizontal gene transfer for *aux* clusters on the chromosome compared to the megaplasmid. Chromosomal *aux* clusters were almost always MGE-associated, which is consistent with prior reports that RSSC prophages are site-specific and are moderately enriched on the evolutionarily stable chromosome (77). In contrast, megaplasmid *aux* clusters were rarely associated with MGEs. Nevertheless, the patterns of phylogenetic distribution suggest that both chromosomal and megaplasmid *aux* clusters have been horizontally transmitted. This opens the question—what genetic mechanisms contributed to horizontal gene flow of megaplasmid *aux* clusters? RSSC are naturally competent (84), so they could readily acquire genes by uptake of environmental DNA and integration by homologous recombination. It would be interesting to test whether there is a bias towards homologous recombination occurring on the megaplasmid. Indeed, the phylogenetic patterns of *aux* cluster content adjacent to the main T6SS island indicate that homologous recombination readily alters which *aux* clusters are encoded there. These putative recombination events might be responsible for the sporadic loss of the conserved *tssL*-to-*tssH* or the conserved the *GH10* to *tagY-*like regions in multiple RSSC genomes.

In closing, we propose that evolution has positioned the RSSC to be a suitable model to understand the role of the T6SS in shaping pathogen populations and host-associated microbial ecosystems. Our systematic analysis opens a plethora of evolutionarily grounded questions for future investigation. For example, do RSSC pathogens target novel cellular targets with their nine toxin families that lack known toxin domains? Are there genetic and epigenetic mechanisms that promote preferential integration of *aux* clusters on the megaplasmid? Moreover, what other physiological functions are enriched on the megaplasmid and secondary replicons in other bacteria with multipartite genomes? RSSC genomes also encode large repertoires of other genes that allow them to sense and change their environments, including root exudate-sensing chemotaxis receptors (18, 85), plant-manipulating T3SS toxins (57), and anti-phage defense systems (86). Do these genes exhibit biased distribution across the replicons? We anticipate that this study will fuel many new discoveries of T6SS biology and pathogen evolution.

## MATERIALS AND METHODS

### Identification of genes encoding the T6SS machinery in RSSC genomes

All publicly available genomes in the genus *Ralstonia*, including *R. syzygii* genomes deposited as “Blood Disease Bacterium” strains, were downloaded from NCBI and uploaded to KBase (87) for analysis. Genomes were analyzed with the Genome Taxonomy Database (GTDB) Toolkit GTDB-Tk*—v1.7.0* to identify the genomospecies (88). We used CheckM*—v1.0.18* to evaluate the completeness and contamination of the assemblies (89). Only genomes with completeness greater than 99.82% and contamination less than 0.96% were retained for further analysis. With the selected assemblies, we generated a phylogenetic tree of the RSSC using KBase Insert Genome into SpeciesTree*—v.2.2.0*. The KBase SpeciesTree App uses 49 genes broadly conserved across bacteria to build a phylogenetic tree with FastTree2 (90). The protein sequence for each GMI1000 T6SS component was queried against the *Ralstonia* genomes using BLASTp*—v2.13.0*. We considered all BLASTp results with ≥20% identity and ≥80% coverage to be hits. We visualized phylogenetic patterns of T6SS gene presence or absence in the RSSC phylogenetic tree using iTOL (91).

Clinker was used to visually compare the genetic architectures of the T6SS main loci from all RSSC and 70 genomes of non-wilt pathogenic *Ralstonia* spp. We downloaded the T6SS main locus region of each genome from NCBI and classified the subtype of T6SS by comparison to reference T6SS loci from plant-colonizing bacteria (3, 32).

We selected high-quality genomes for detailed analysis of the repertoires of *vgrG*-linked toxin/immunity gene clusters (*aux* clusters). Limiting the analysis to complete genomes would have excluded almost all phylotype II, III, and IV genomes, so we included 99 genomes assembled into as many as 28 contigs. We carried out a series of low-stringency BLASTp searches against the RSSC genomes with multiple VgrG protein sequences from phyl. I GMI1000, phyl. II IBSBF1503, and phyl. IV PSI07 (Parameters: ≥1% identity, ≥1% coverage, and Bit Score ≥10). All VgrG BLASTp results were merged and further analyzed in Excel and iTOL.

### Identification of T6SS and *vgrG* genes in representative Burkholderiaceae genomes

The GTDB (88) was used to identify complete genomes in the Burkholderiaceae family. Per GTDB genomospecies (based on 95% ANI threshold), we selected one representative genome to import into a KBase Narrative (https://narrative.kbase.us/narrative/142785). We used BLASTp as described above to identify T6SS genes. The full results are presented in Table S1.

In a complementary approach, we used HMMER with JackHMMER (24) to identify T6SS gene homologs in the Burkholderiaceae genomes. We searched for core RSSC T6SS genes, querying amino acid sequences from *R. pseudosolanacearum* GMI1000. The *E*-value threshold for inclusion was 0.001, and the maximum number of JackHMMER iterations was *n* = 5.

### Classifying *vgrG*-linked toxin/immunity gene clusters through synteny analysis

Identifying the complete *aux* cluster repertoire of each RSSC genome was an iterative process that involved BLASTp searches and synteny analysis. We used a low-stringency BLASTp search to identify putative *vgrG* genes and downloaded a Genbank Flat file from NCBI that encompassed a 10–200 kb region surrounding each. Iterative synteny analysis of the *vgrG* regions was performed using Clinker (37) to identify gene clusters with shared genetic architecture. Clusters with shared genetic architecture were assigned an *aux* number (e.g., *aux2*) that was used for downstream analyses. In *aux* clusters with fragmented *vgrG* genes, we tabulated the locus tag for the upstream fragment in Table S3.

We realized our approach would not find *aux* clusters that did not have *vgrG* genes. In cases where a strain lacked an *aux* cluster type found in closely related strains, we used synteny analysis to determine whether the strain truly lacked the *aux* cluster by searching the genome region for the other genes associated with that *aux* cluster. For *aux* clusters lacking *vgrG* genes, we tabulated the locus tag of the most upstream in Table S3.

Genome annotations were used to identify pseudogenized genes in each *aux* cluster, focusing especially on *vgrG,* toxin, and immunity genes. By inspecting *aux* cluster alignments in Clinker, we were additionally able to identify missing genes or genes pseudogenized by transposons or insertion sequences. Clusters with at least one missing or pseudogenized *vgrG* or toxin gene were classified as “decayed” and are hypothesized to not be functional. We additionally noted the presence of “orphan” immunity genes, which lack an intact corresponding *vgrG* and toxin.

### Identifying toxins, immunity proteins, and adaptors encoded in each *aux* cluster

We queried putative RSSC *aux* protein sequences against the literature using PaperBlast (39). Additionally, we identified sequences containing domains associated with T6SS toxins, immunity proteins, and adaptor proteins by searching the NCBI CDD using CD-Search (38). Toxins were identified on the basis of the presence of known T6SS toxin domains or homology with *bona fide* T6SS toxins [e.g., PaperBlast matches to Tle1, Tle3, Tle4, and Tle5 phospholipases (49)]. Immunity proteins were identified based on the presence of known T6SS immunity domains and homology to *bona fide* immunity proteins (PaperBlast). Adapters were identified based on the presence of domains including DUF1795, DUF2169, or DUF4123.

### Identifying the genomic location of each *vgrG*-linked *aux* cluster

We used Clinker to compare all loci for *aux* clusters to the genome of *R. pseudosolanacearum* GMI1000. We inspected the Clinker alignments of the unique genetic neighborhoods flanking each *aux* cluster and the the corresponding neighborhood in the GMI1000 genome to designate the neighborhoods as a specific location (loc). We subsequently referred to groups’ *aux* clusters with the same genetic architecture and neighborhood as an *aux^loc^* combination (e.g., *aux2^loc1^*). This approach allowed us to predict the chromosomal vs. megaplasmid locations of the *aux^loc^* groups in all 99 analyzed RSSC genomes, including genomes that were not fully resolved. For downstream analyses, we recorded the GMI1000 locus tag for the *vgrG* gene if GMI1000 encoded the same cluster. If GMI1000 did not encode the same cluster, we recorded the locus tag of the nearest ortholog upstream of the *vgrG* gene. These loci are listed as the “nearby location markers” (Table S3) and were used to graphically depict *aux^loc^* locations in each T6SS atlas entry (Fig. S5 to S26) and to record the replicon in Table S3.

### Hierarchical clustering of RSSC *aux* types by their phylogenetic distribution

To compare the phylogenetic distribution of *aux* types in RSSC genomes, we first used the “decostand” function from the R package “vegan v.2.6-2” to calculate the relative abundance of each *aux* type per RSSC genome. We used these relative abundances to generate a distance matrix using the “vegdigest” function. To generate a dendrogram from the *aux* type distance matrix, we used the function “hclust” from the package “Phangorn v.2.11.2” in Rstudio [version 2023.03.0 + 386 (92)].

### Identifying mobile genetic elements associated with *aux* clusters

To identify MGEs associated with various T6SS *aux* clusters, we searched the genetic neighborhoods within 100 kb of each *aux* cluster using the PHASTER prophage identification tool (55). In parallel, we inspected gene annotations of the *aux* cluster genetic neighborhoods for signs of MGEs. For genes encoding hypothetical proteins, we searched for domains associated with MGEs using NCBI CDD (38). Based on these observations, we predicted if each *aux* cluster was likely associated with an MGE (Fig. S6; Table S3). In some instances where there was weak evidence of association with an MGE, we designated the MGE association of *aux* clusters as “inconclusive.” Additionally, we visualized the shared genetic context of these MGE-associated *aux* clusters using Clinker.

## Data Availability

Toxin arsenals of other *Ralstonia* genomes can be classified using Clinker and the reference genbank (.gbk) files for *aux1-aux25* available on FigShare (https://doi.org/10.6084/m9.figshare.23065583). Additionally, representative interactive *aux* cluster alignment .html files that were generated with Clinker can be downloaded from the same repository. PDF versions of the species trees with T6SS gene annotations and genome accession numbers can be viewed in this linked FigShare repository (https://doi.org/10.6084/m9.figshare.23499141.v1). The R script used to compute the UPGMA tree to organize the *aux* cluster types is available on FigShare (https://doi.org/10.6084/m9.figshare.23065583). KBase narratives to explore genomes used in this study can be accessed with a free KBase account as long as KBase is funded; RSSC https://narrative.kbase.us/narrative/123807, Burkholderiaceae species representatives https://narrative.kbase.us/narrative/142785, Other *Ralstonia* spp. https://narrative.kbase.us/narrative/127696, *Cupriavidus* spp. https://narrative.kbase.us/narrative/142720, and *Pandoreae* spp. https://narrative.kbase.us/narrative/142975. Additionally, the NCBI accessions for each genome are included within Tables S1 and S2.

## SUPPLEMENTAL MATERIAL

Supplemental Figures mbio.00323-24-s0001.docx Figures S1-S34. Includes detailed atlas pages for each of the 25 RSSC aux clusters.

Table S1 mbio.00323-24-s0002.xlsx BLASTp results of Burkholderiaceae species T6SS genes.

Table S2 mbio.00323-24-s0003.xlsx BLASTp results of RSSC species T6SS genes.

Table S3 mbio.00323-24-s0004.xlsx Details of aux clusters and associated-MGE.

ASM does not own the copyrights to Supplemental Material that may be linked to, or accessed through, an article. The authors have granted ASM a non-exclusive, world-wide license to publish the Supplemental Material files. Please contact the corresponding author directly for reuse.
