# Supplementary material for: A pangenomic atlas reveals eco-evolutionary dynamics that shape type VI secretion systems in plant-pathogenic Ralstonia
Source: mBio. 2024 Aug 27;15(10):e00323-24. doi: 10.1128/mbio.00323-24 (PMC11481896; doi:10.1128/mbio.00323-24)
Supplement: Supplemental Figures — Figures S1-S34. Includes detailed atlas pages for each of the 25 RSSC aux clusters. [file mbio.00323-24-s0001.docx]

**Table S1. BLASTp results of Burkholderiaceae species T6SS genes**

**Table S2. BLASTp results of RSSC species T6SS genes**

**Table S3. Details of *aux* clusters and associated-MGE**


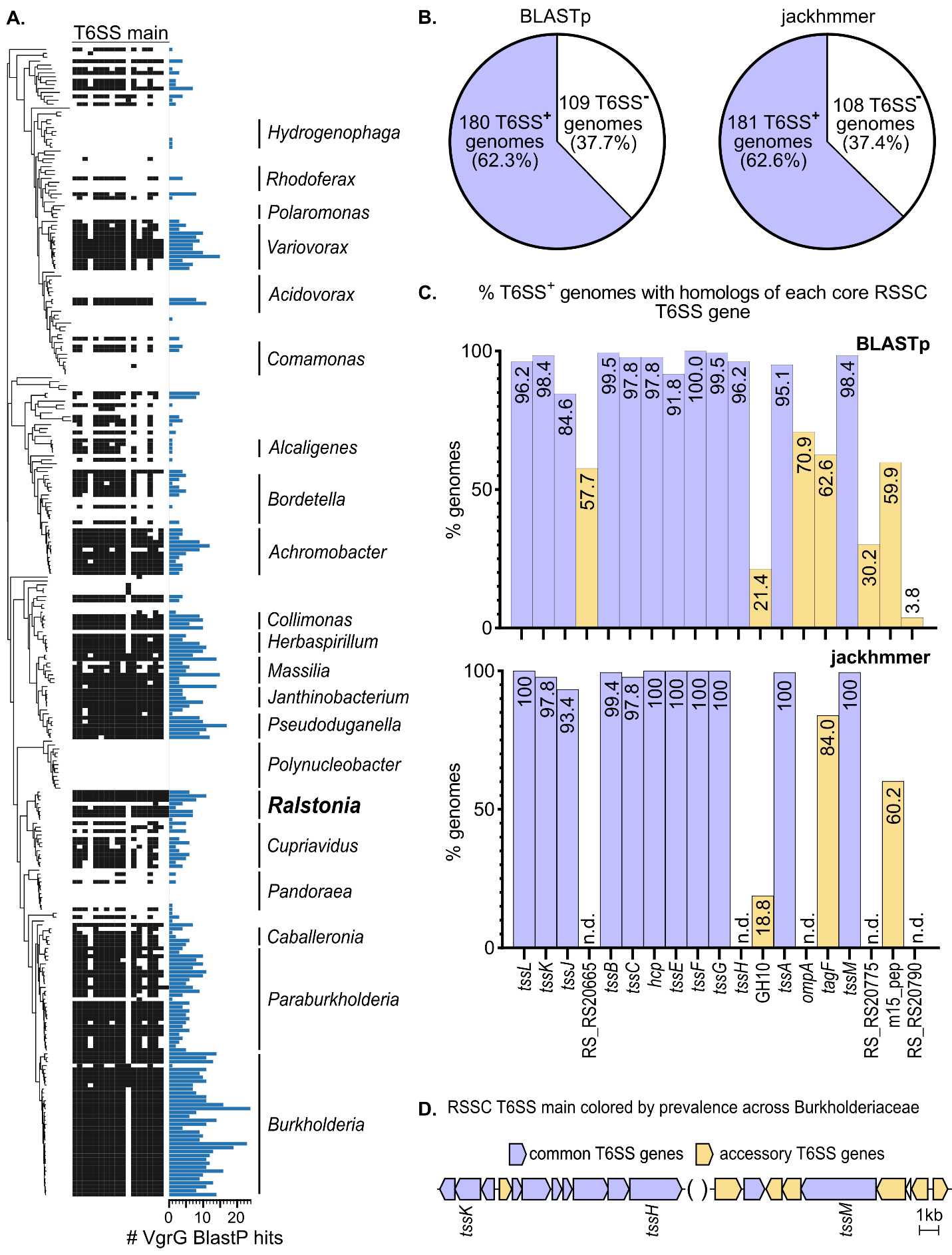


**Figure S1.** **Diverse Burkholderiaceae species representatives encode some, but not all, of the conserved RSSC T6SS main genes.** We investigated the prevalence of T6SS genes across diverse Burkholderiaceae genomes. Using the Genome Taxonomy Database (GTDB), we identified complete genomes that represent 289 genomospecies within the Burkholderiaceae family. We built a custom database of these genomes in KBase and carried out BLASTp searches of 19 genes that all T6SS^+^ RSSC strains encode in the main locus and estimated VgrG copy number with BLASTp. **(A)** Phylogenetic distribution of BLASTp hits for presence/absence of T6SS genes and copy number of VgrG homologs. The order of the T6SS genes is the same as the bar chart in **(C)**. Genera with many species representatives are labeled. A PDF of the full tree is available on FigShare: https://doi.org/10.6084/m9.figshare.23499141.v1. **(B)** The proportion of the 289 genomes that encode at least one T6SS. **(C)** The prevalence of RSSC’s core T6SS genes across Burkholderiaceae genomes. Blue indicates genes identified in most T6SS^+^ genomes and yellow indicates genes that are more rarely encoded. The conserved and variable T6SS genes are enriched in different clusters of the RSSC T6SS main genes. Finding homologs with jackhmmer resulted in an overcount of some T6SS-related genes that have many distant, non-T6SS-related homologs, and are marked as “n.d.” for “not determined” in the bar plot. **(D)** Schematic of the RSSC core genes colored by their prevalence across the Burkholderiaceae.


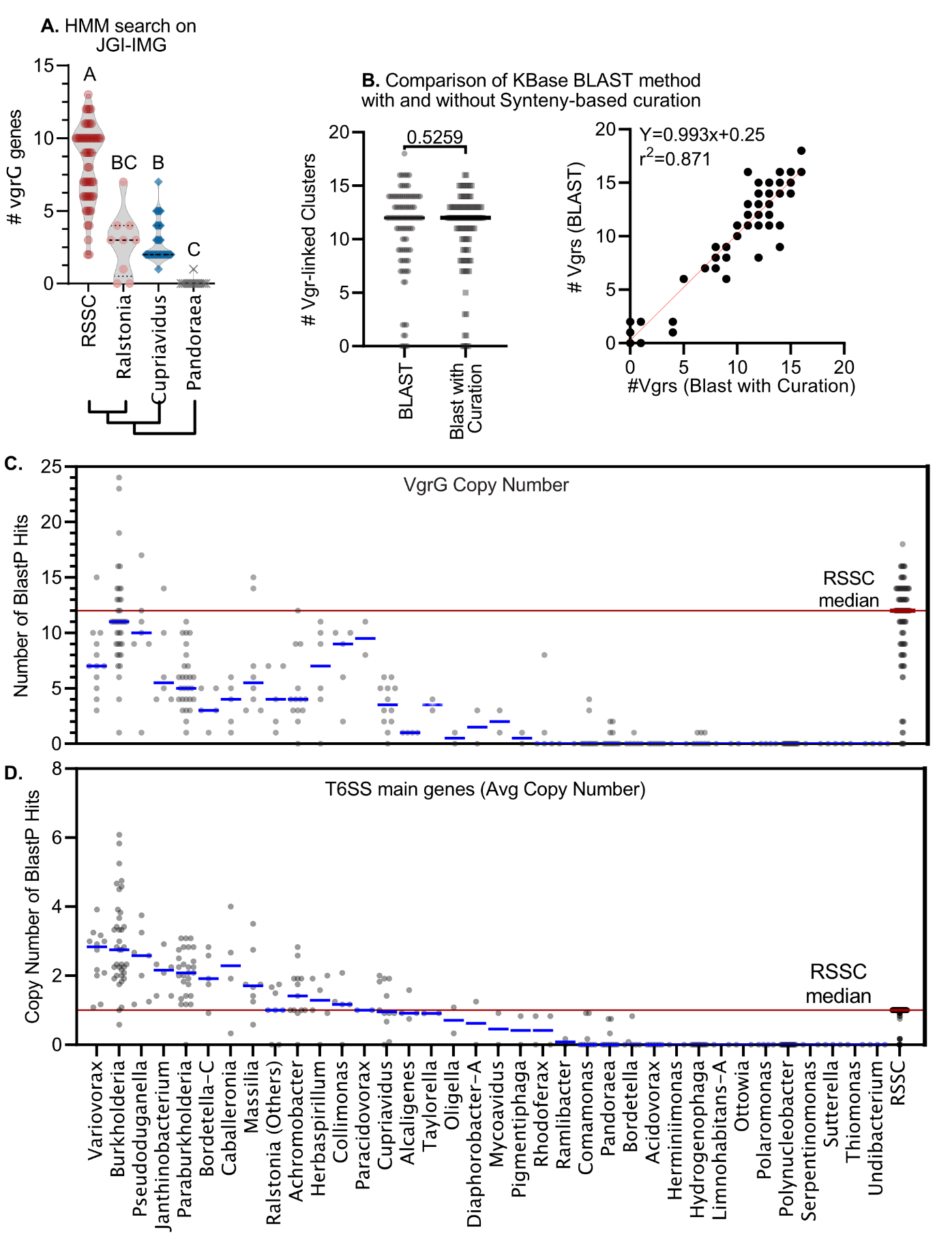


**Figure S2. Additional Data showing RSSC genomes are enriched in *vgrG*-linked toxin/anti-toxin clusters.** We quantified VgrG abundance in RSSC genomes with three independent methods: (**A-B**) HMM-based searches with TIGR03361 on the JGI IMG platform, BLASTp-based searches with six distinct VgrG homologs using custom genome database**s** on KBase, and curation of the BLASTp search results for high quality RSSC genomes using Clinker synteny analysis for regions surrounding Blast results for VgrG, toxin, or antitoxin genes. (**B**) The results of the BLASTp-only approach were similar to results of the BLASTp-with-curation approach (Mann-Whitney test; p=0.5259). A linear regression between the two approaches is displayed on the XY plot (y=0.993x+0.25 with r^2^ = 0.871). (**C-D**) Comparison of T6SS gene content across Burkholderiaceae genomes corresponding to Figure 1A. For the T6SS main genes, each circle represents a single genome’s average number of BLASTp hits for the Core T6SS components: TssA, TssB, TssC, TssE, TssF, TssG, TssH, TssJ, TssK, TssL, TssM, and Hcp.


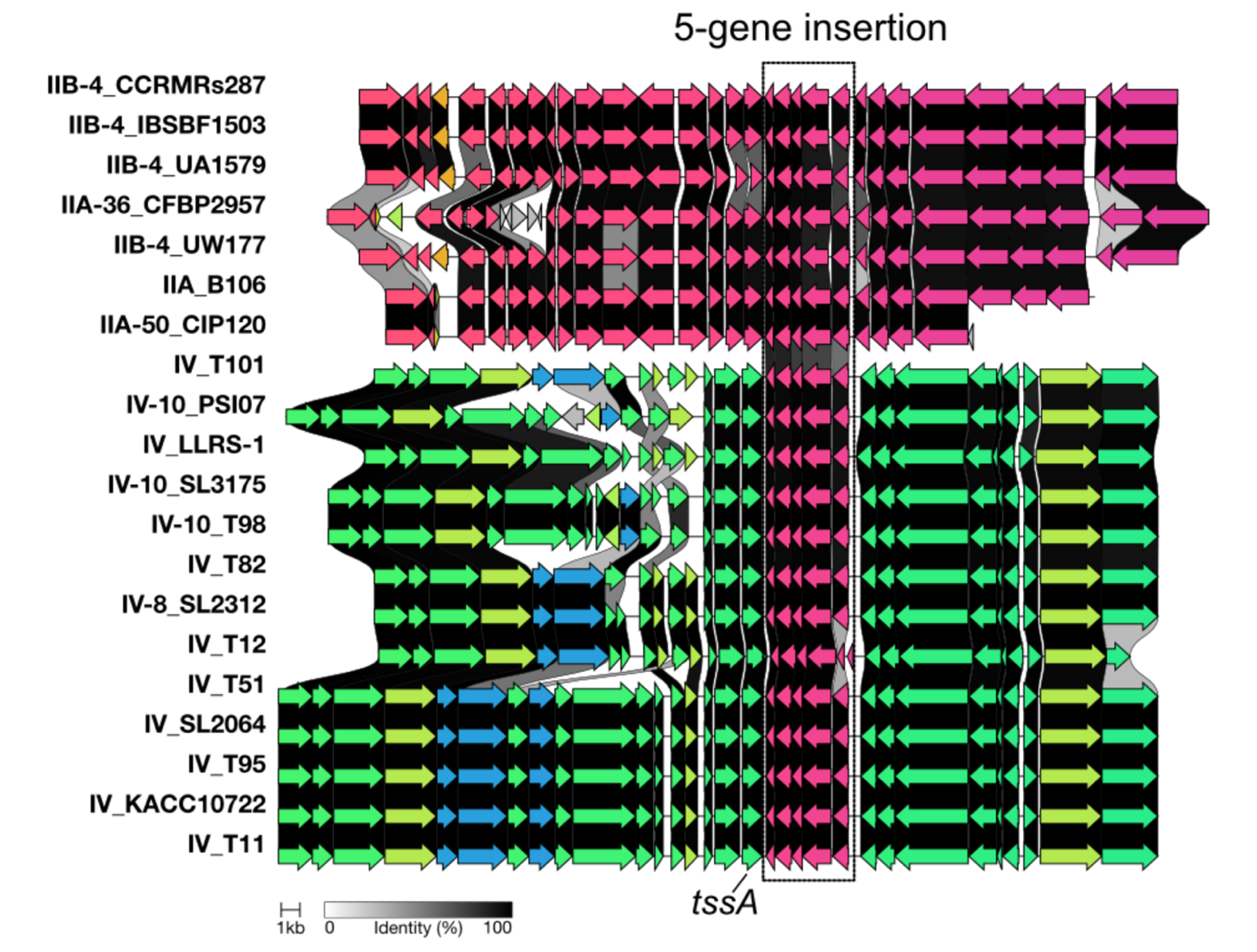
**Figure S3. The 5-gene insertion downstream of *tssA* in phylotype IV genomes is found in a distinct genetic locus in phylotype II genomes.** The five-gene insertion is highlighted in the dotted black rectangle. The five-gene insertion downstream of *tssA* includes genes encoding a DUF393 domain-containing protein, an HTTM domain-containing protein, a hypothetical protein, a serine hydrolase domain-containing protein, and a hypothetical protein. While phylotype IV genomes encode this five-gene cluster between *tssA* and *gh10* in the T6SS main locus, phylotype II genomes encode the same cluster elsewhere on the megaplasmid.


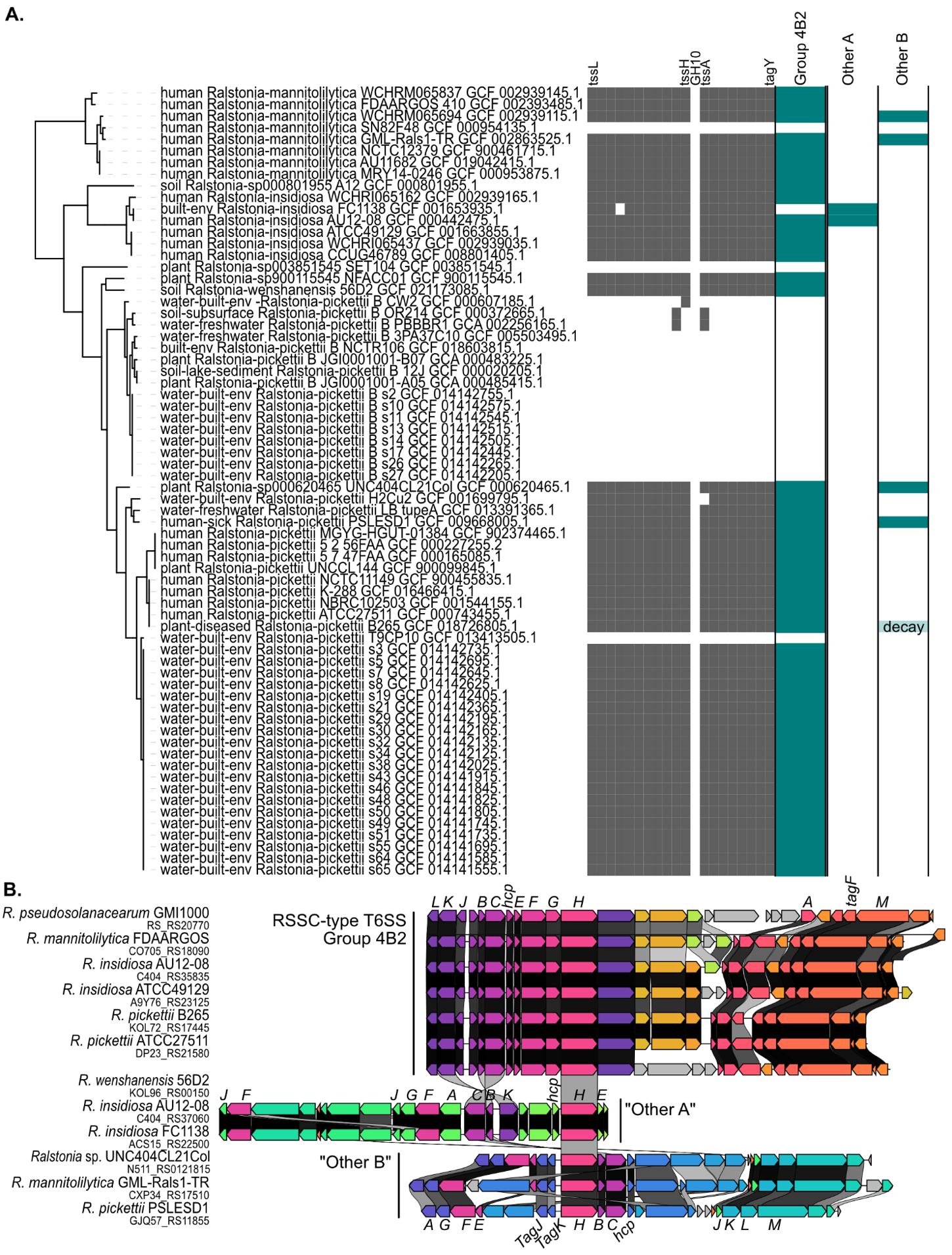
**Figure S4. The RSSC T6SS is broadly conserved across the *Ralstonia* genus.** (A) Left: phylogenetic tree of non-RSSC *Ralstonia* genomes, generated using the KBase SpeciesTree app. Middle: Gray squares show the presence of the RSSC T6SS main genes across the non-RSSC *Ralstonia* genomes. Right: Teal rectangles show the presence of the RSSC-type T6SS (a type i-4B2) or two rare types of T6SS clusters, informally named "Other A” and “Other B” here. (B) Synteny of representative T6SS main gene clusters from the three types.


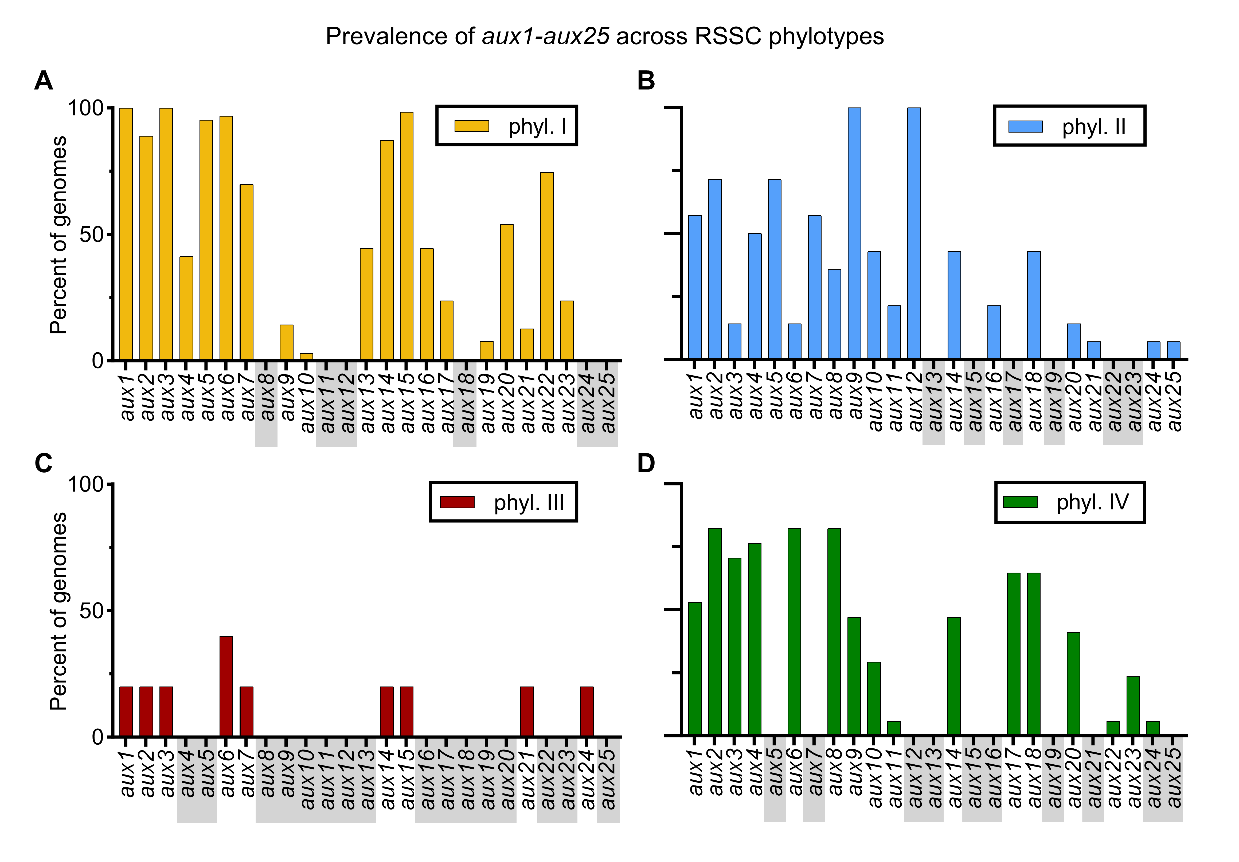


**Figure S5. RSSC lineages vary in their repertoires of auxiliary *vgrG*-linked toxin/antitoxin clusters (*aux* clusters).** *Aux* clusters were identified in complete or nearly complete genomes by BLASTp searches for genes encoding Vgr, toxins, or immunity proteins. The *aux* clusters were classified by synteny analysis with Clinker. (**A-D)** *aux* clusters vary in their prevalence in different RSSC phylotypes (n=775 *aux* clusters in phylotype I, n=135 *aux* clusters in phyl. II, n=10 in phyl. III, n=149 in phyl IV). Gray rectangles identify *aux* clusters that were not identified in the genomes of strains in each phylotype.


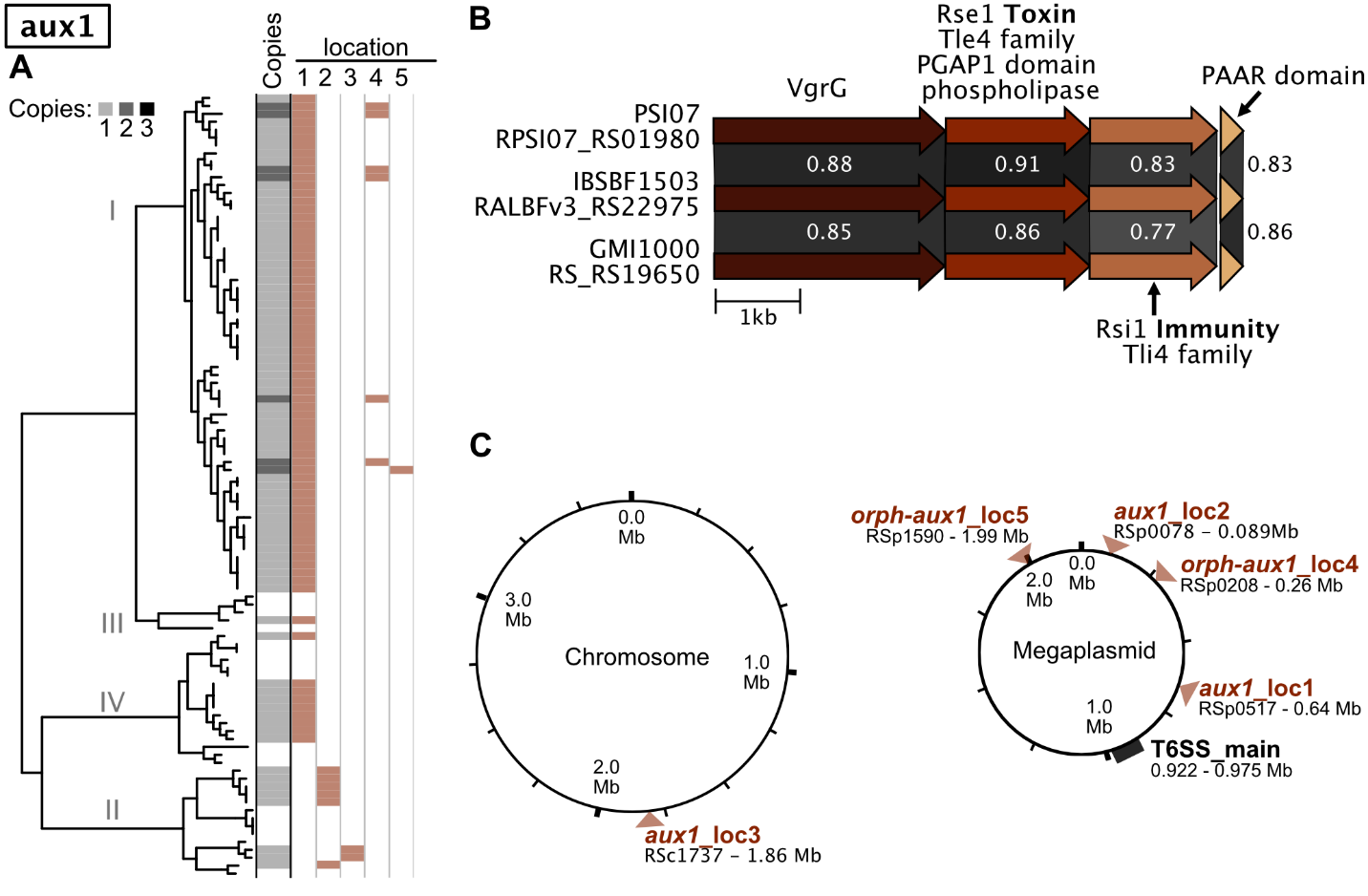


**Figure S6. Phylogenetic distribution, genetic organization/synteny, and chromosomal location of auxiliary *vgrG*-linked cluster 1 (*aux1*).** (**A**) Phylogenetic distribution and copy number of *aux1* across high-quality RSSC genomes. (**B**) Genetic organization/synteny of *aux1* from 3 genomes: phyl. I GMI1000, phyl. IIB-4 IBSBF1503, and IV-10 PSI07. *Aux1* encodes a VgrG, a Tle4-family phospholipase with a PGAP1 domain, a Tli4 immunity protein, and a PAAR domain protein. Grayscale links indicate the amino acid identity between homologs. (**C**) *aux1* clusters were identified at five locations across the chromosome and megaplasmid, and these locations are shown relative to the GMI1000 replicons. Panel A indicates which genomes encode *aux1* at each location. The figure was generated with a combination of KBase BLASTp, iToL, Clinker, and Affinity Designer.


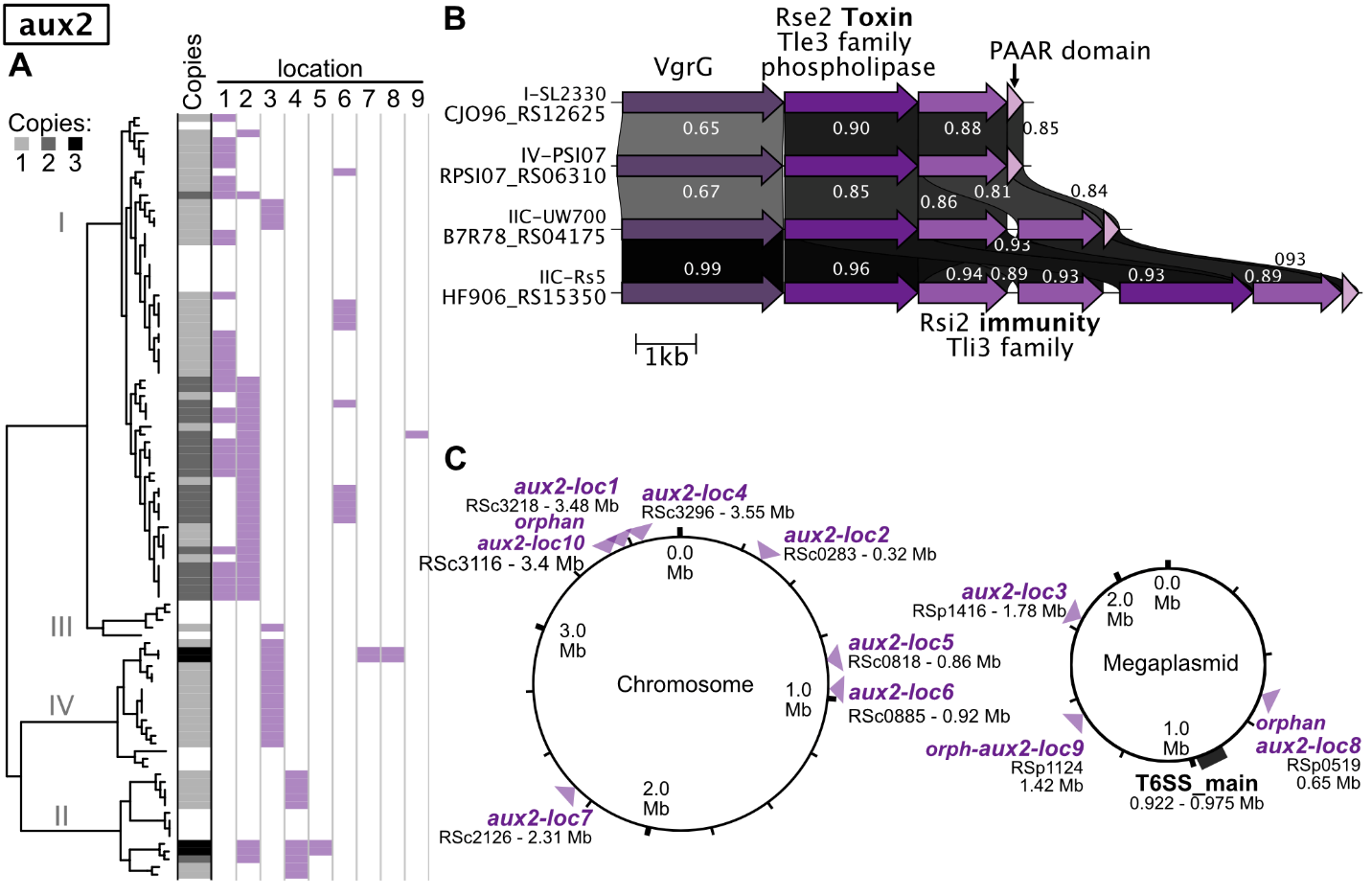


**Figure S7. Phylogenetic distribution, genetic organization/synteny, and chromosomal location of auxiliary *vgrG*-linked cluster 2 (*aux2*).** (**A**) Phylogenetic distribution and copy number of *aux2* across high-quality RSSC genomes. (**B**) Genetic organization/synteny of the cluster from 4 genomes: phyl. I SL2330, phyl. IIC-7 UW700, IIC-7 Rs5, and IV-10 PSI07. *Aux2* encodes a VgrG, a Tle3-family phospholipase, one-or-more Tli3 immunity protein(s), and a PAAR domain protein. Grayscale links indicate the global amino acid identity between homologs. (**C**) *aux2* clusters were identified at nine locations across the chromosome and megaplasmid, and these locations are shown relative to the GMI1000 replicons. Panel A indicates which genomes encode the cluster at each location. The figure was generated with a combination of KBase BLASTp, iToL, Clinker, and Affinity Designer.


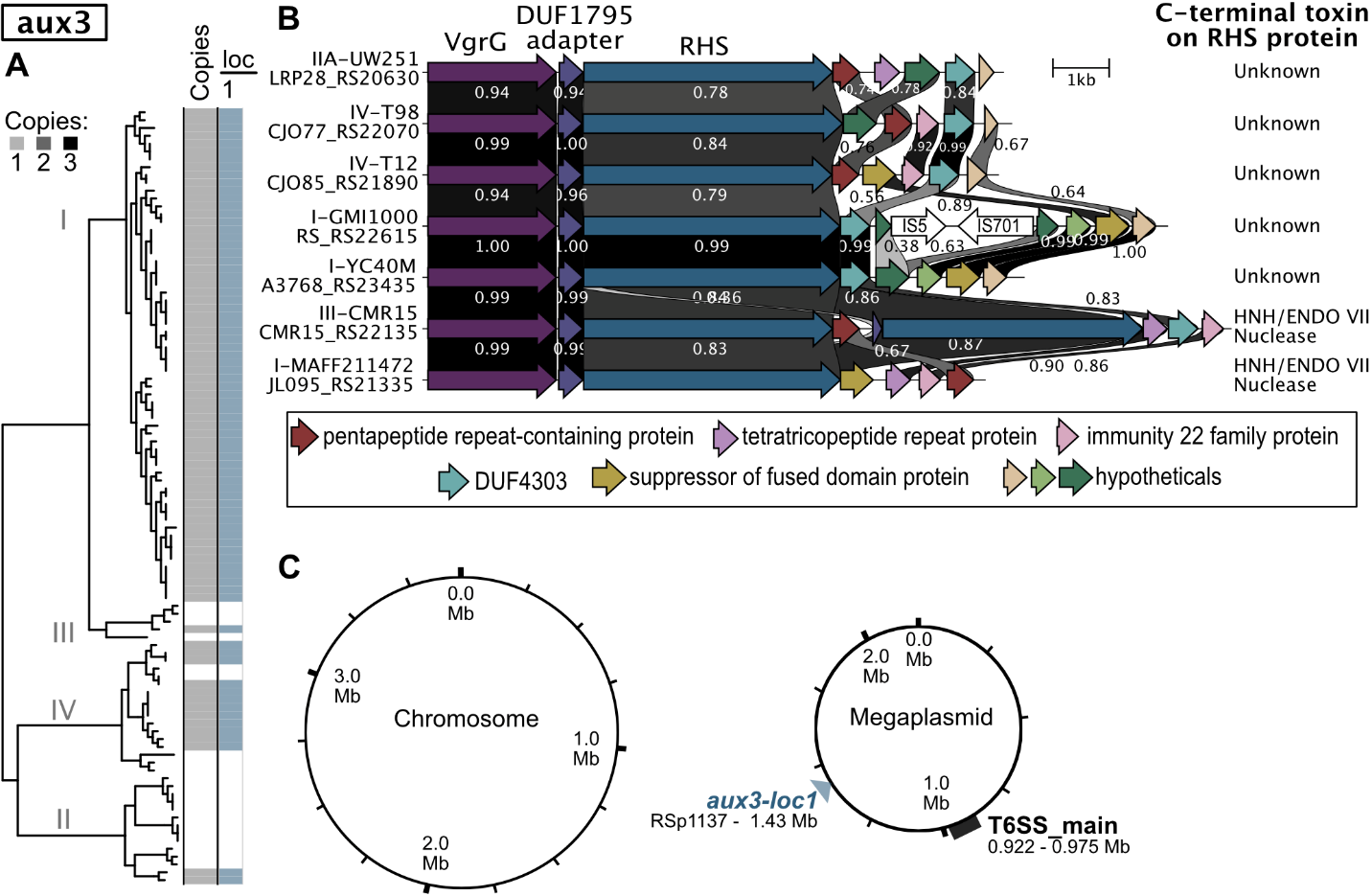


**Figure S8. Phylogenetic distribution, genetic organization/synteny, and chromosomal location of auxiliary *vgrG*-linked cluster 3 (*aux3*).** (**A**) Phylogenetic distribution and copy number of *aux3* across high-quality RSSC genomes. (**B**) Genetic organization/synteny of the cluster from 7 genomes: phyl. I GMI1000, phyl. I MAFF211472, phyl. I YC40M, phyl. IIA UW251, phyl. III CMR15, phyl. IV T98, and phyl. IV T12. *Aux3* encodes a VgrG, a DUF1795 adapter protein, an RHS with a putative C-terminal toxin, and four-or-more variable proteins: a pentapeptide repeat-containing protein, a tetratricopeptide repeat protein, and imm22 family immunity protein, a DUF4303, a suppressor of fused domain protein, and hypothetical(s). Some of the C-terminal toxins are putative HNH/ENDO VII nucleases while others do not encode known domains. The small genes downstream of the RHS toxin are likely an array of immunity proteins. Grayscale links indicate the global amino acid identity between homologs. (**C**) *aux3* clusters were identified at one location on the megaplasmid, and the location is shown relative to the GMI1000 replicons. The figure was generated with a combination of KBase BLASTp, iToL, Clinker, and Affinity Designer.


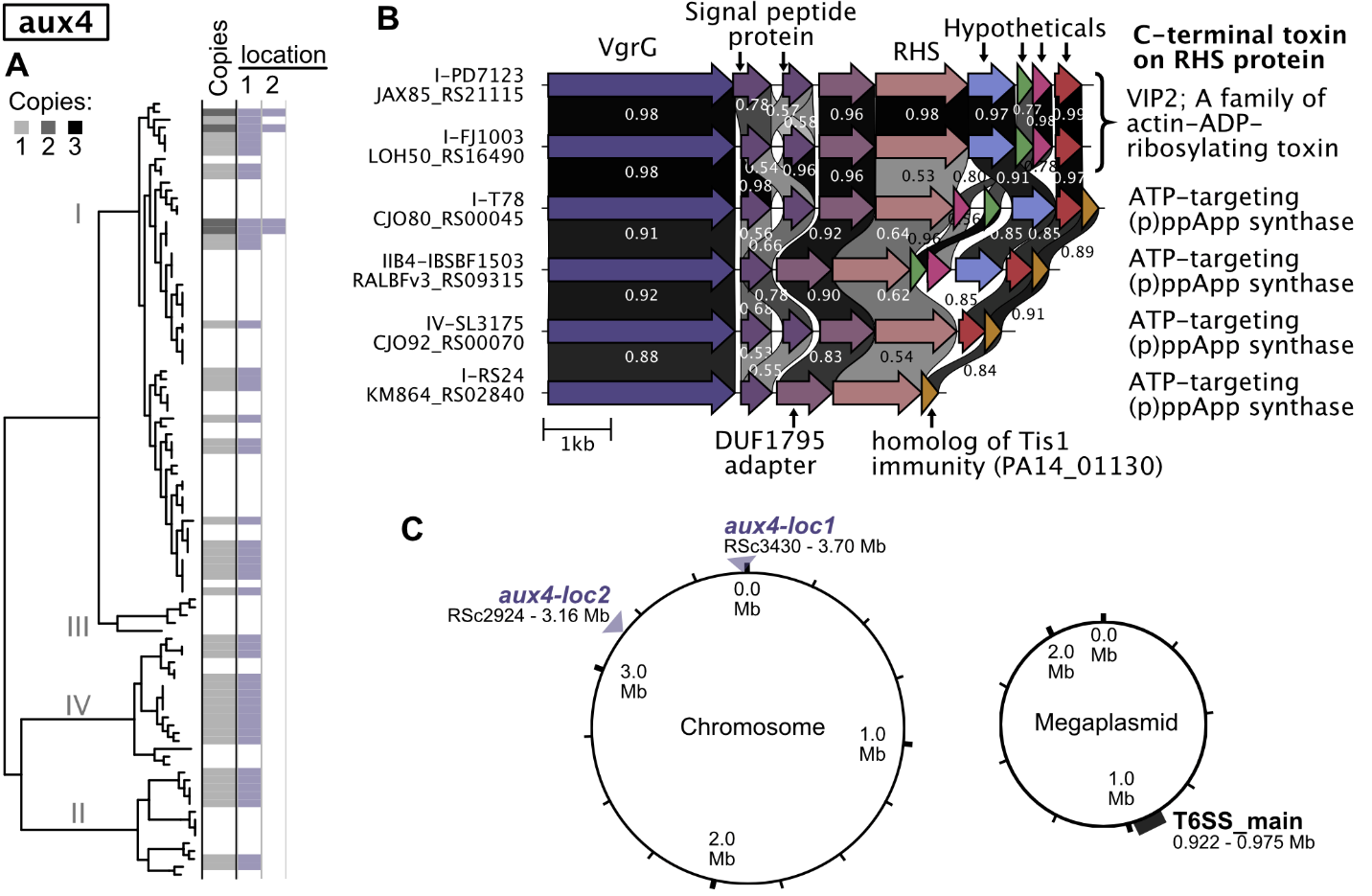


**Figure S9. Phylogenetic distribution, genetic organization/synteny, and chromosomal location of auxiliary *vgrG*-linked cluster 4 (*aux4*).** (**A**) Phylogenetic distribution and copy number of *aux4* across high-quality RSSC genomes. (**B**) Genetic organization/synteny of the cluster from 6 genomes: phyl. I PD7123, phyl. I FJ1003, phyl. I T78, phyl. I RS24, phyl. IIB-4 IBSBF1503, and IV SL3175. *Aux4* encodes a VgrG, one-or-more signal peptide proteins, a DUF1795 adapter protein, an RHS with a putative C-terminal toxin, and one-or-more variable proteins: a homolog of the *P. aeruginosa* Tis1 immunity protein or four distinct hypothetical(s). The small genes downstream of the RHS toxin likely encode an array of immunity proteins. Some of the C-terminal toxins are annotated as VIP2 actin-ADP-ribosylating toxins while others have similarity to the ATP-targeting (p)ppApp synthases Tas1 (Ahmad et al., 2019), including conserved active site motifs and the catalytic glutamate. Grayscale links indicate the global amino acid identity between homologs. (**C**) *aux4* clusters were identified at two locations across the chromosome and megaplasmid, and these locations are shown relative to the GMI1000 replicons. Panel A indicates which genomes encode the cluster at each location. The figure was generated with a combination of KBase BLASTp, iToL, Clinker, and Affinity Designer.


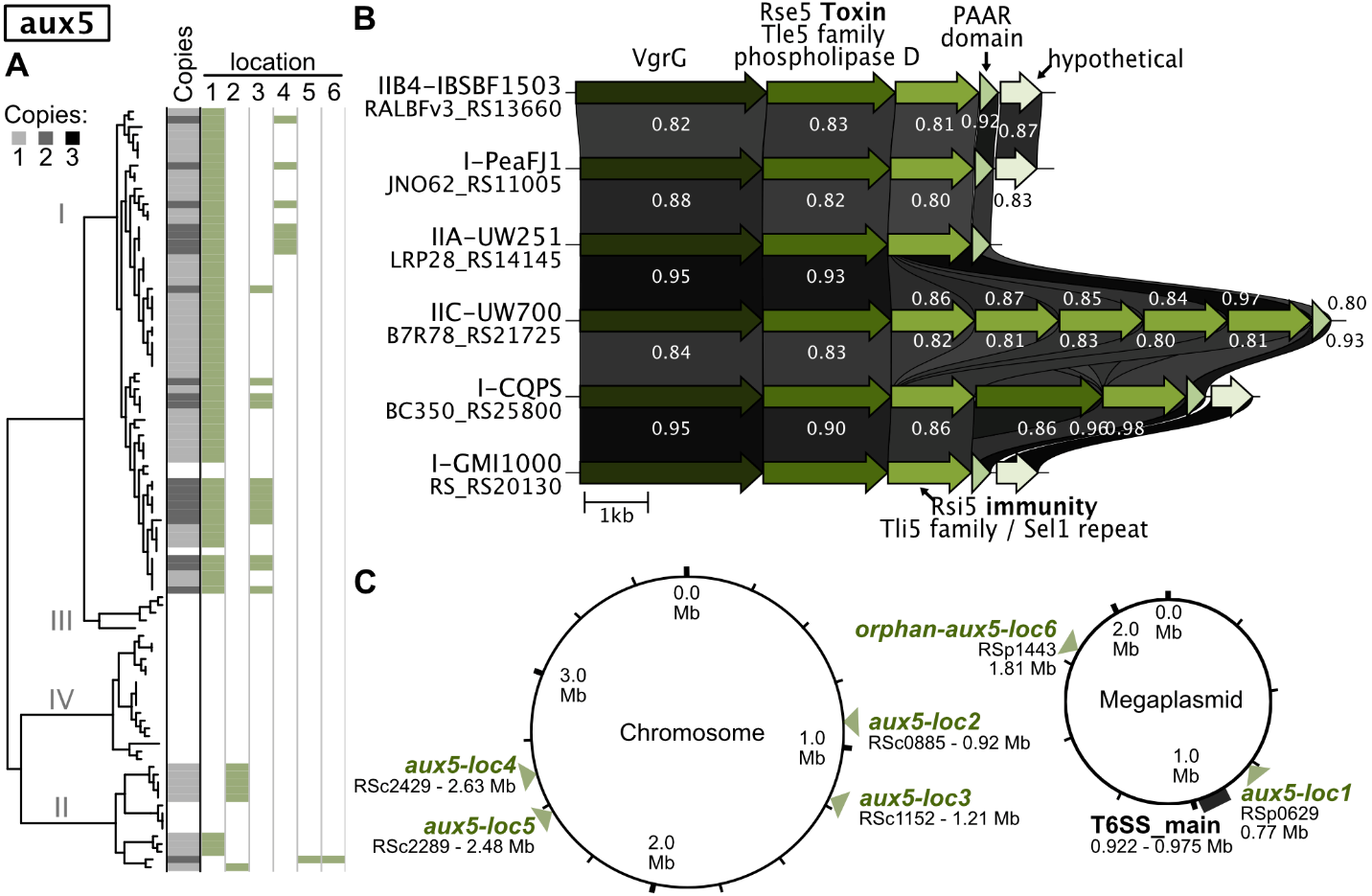


**Figure S10. Phylogenetic distribution, genetic organization/synteny, and chromosomal location of auxiliary *vgrG*-linked cluster 5 (*aux5*).** (**A**) Phylogenetic distribution and copy number of *aux5* across high-quality RSSC genomes. (**B**) Genetic organization/synteny of the cluster from 6 genomes: phyl. I PeaJF1, phyl. I CQPS, phyl. I GMI1000, phyl. IIA UW251, phyl. IIB-4 IBSBF1503, and IIC-7 UW700. *Aux5* encodes a VgrG, a Tle5-family phospholipase D, one-or-more Tli5 immunity protein(s) with Sel1 repeats, a PAAR domain protein, and a variably present hypothetical protein. Grayscale links indicate the global amino acid identity between homologs. (**C**) *aux5* clusters were identified at six locations across the chromosome and megaplasmid, and these locations are shown relative to the GMI1000 replicons. Panel A indicates which genomes encode the cluster at each location. The figure was generated with a combination of KBase BLASTp, iToL, Clinker, and Affinity Designer.


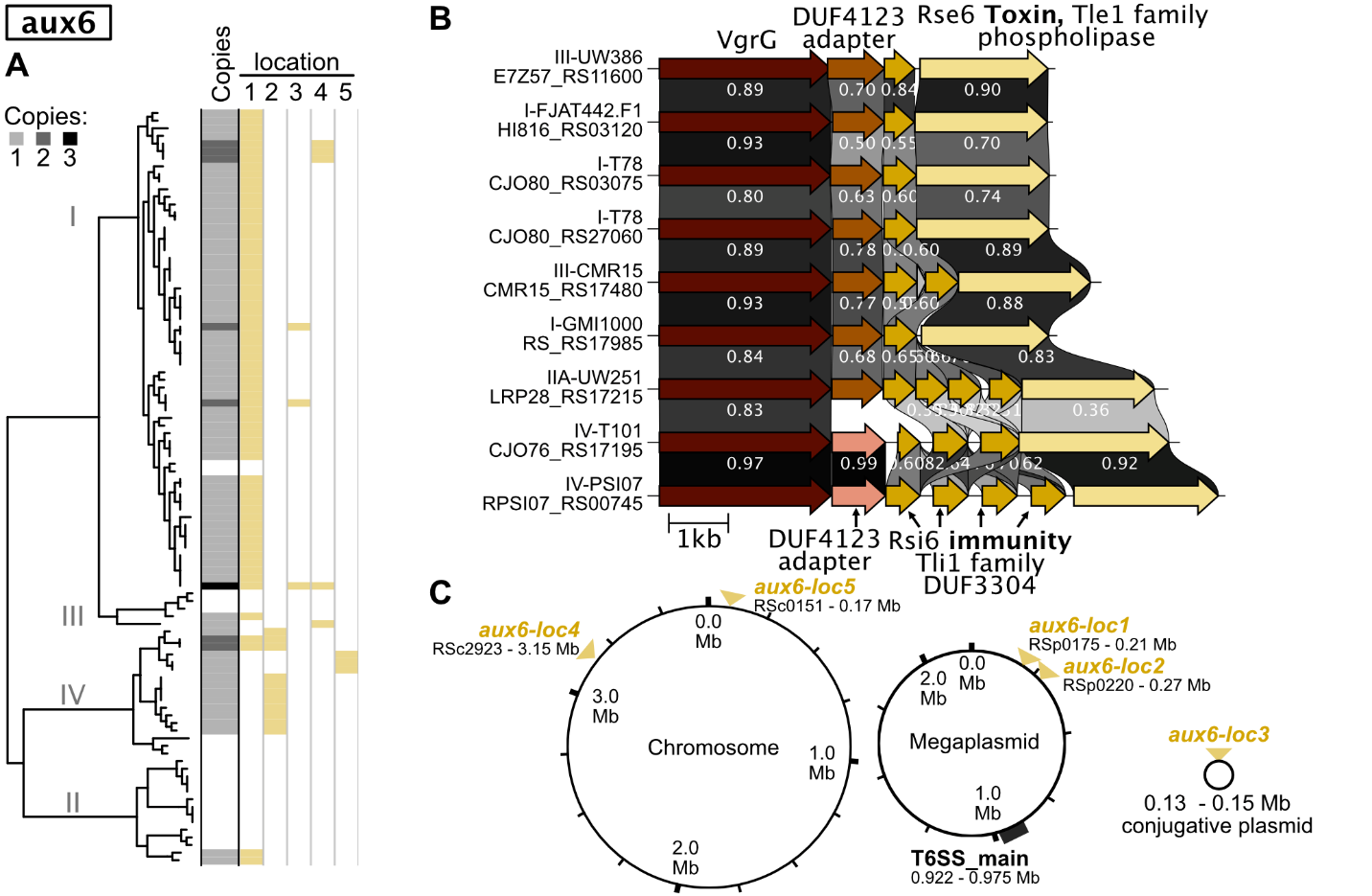


**Figure S11. Phylogenetic distribution, genetic organization/synteny, and chromosomal location of auxiliary *vgrG*-linked cluster 6 (*aux6*****).** (**A**) Phylogenetic distribution and copy number of *aux6* across high-quality RSSC genomes. (**B**) Genetic organization/synteny of the cluster from 9 genomes: phyl. I FJAT442.F1, two paralogous clusters from phyl. I T78, phyl. I GMI1000, phyl. IIA UW251, phyl. III CMR15, phyl. III UW386, phyl. IV T101, and phyl. IV PSI07. *Aux6* encodes a VgrG, a DUF4123 adaptor protein, one-or-more Tli1 family immunity proteins with a DUF3304 domain, and a Tle1 family phospholipase toxin. Grayscale links indicate the global amino acid identity between homologs. (**C**) *aux6* clusters were identified at five locations across the chromosome and megaplasmid, and these locations are shown relative to the GMI1000 replicons. Panel A indicates which genomes encode the cluster at each location. The figure was generated with a combination of KBase BLASTp, iToL, Clinker, and Affinity Designer.


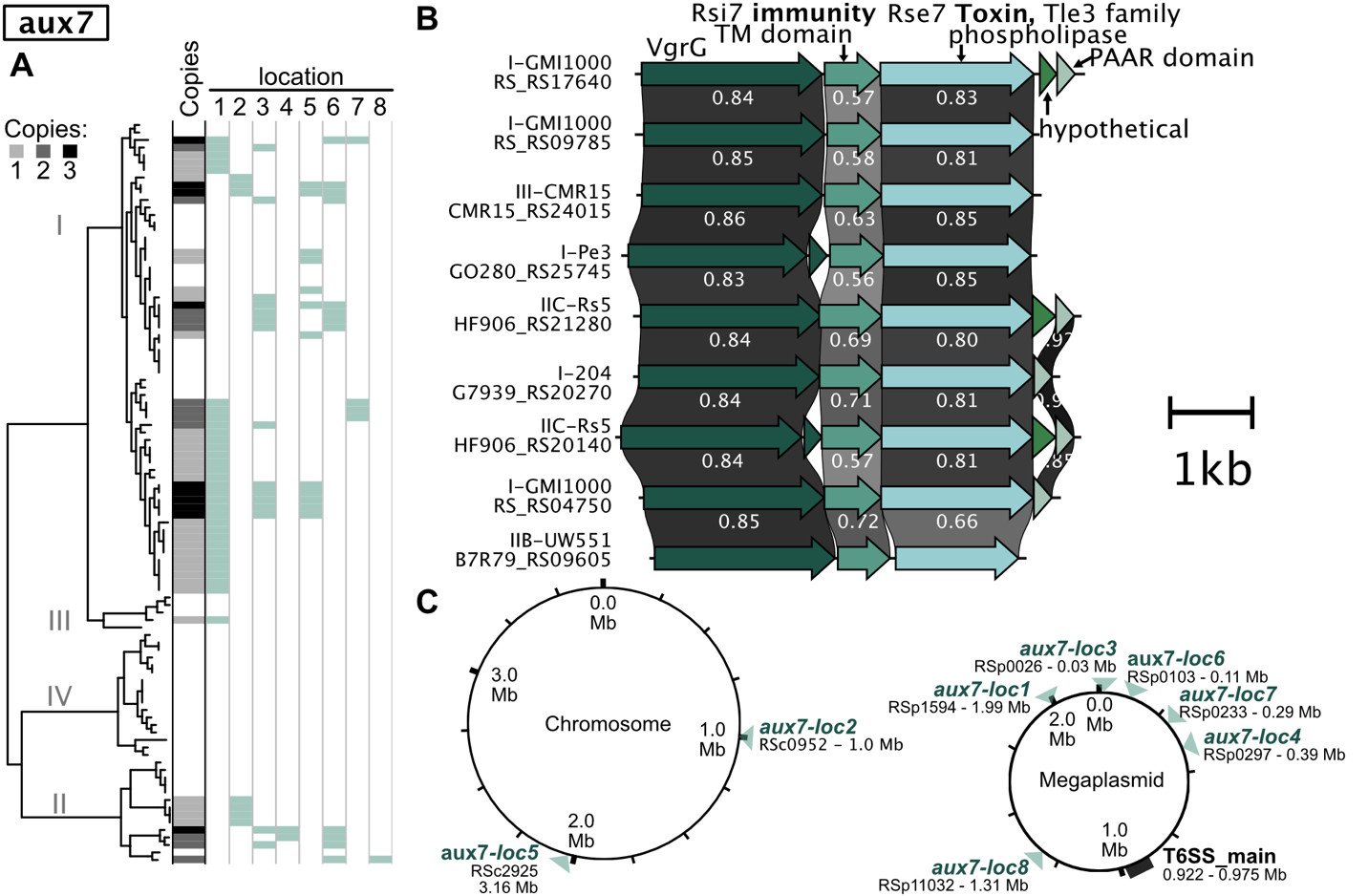


**Figure S12. Phylogenetic distribution, genetic organization/synteny, and chromosomal location of auxiliary *vgrG*-linked cluster 7 (*aux7*).** (**A**) Phylogenetic distribution and copy number of *aux7* across high-quality RSSC genomes. (**B**) Genetic organization/synteny of the cluster from 9 genomes: three distinct paralogous clusters from phyl. I GMI1000, phyl. I Pe3, phyl. I 204, phyl. IIB-1 UW551, two paralogous clusters from phyl. IIC-7 Rs5, and phyl. III CMR15. *Aux7* encodes a VgrG, an immunity protein with a transmembrane (TM) domain, a Tle3-family phospholipase toxin, a variably present PAAR domain protein, and a variably present hypothetical protein. Grayscale links indicate the global amino acid identity between homologs. (**C**) *aux7* clusters were identified at seven locations across the chromosome and megaplasmid, and these locations are shown relative to the GMI1000 replicons. Panel A indicates which genomes encode the cluster at each location. The figure was generated with a combination of KBase BLASTp, iToL, Clinker, and Affinity Designer.


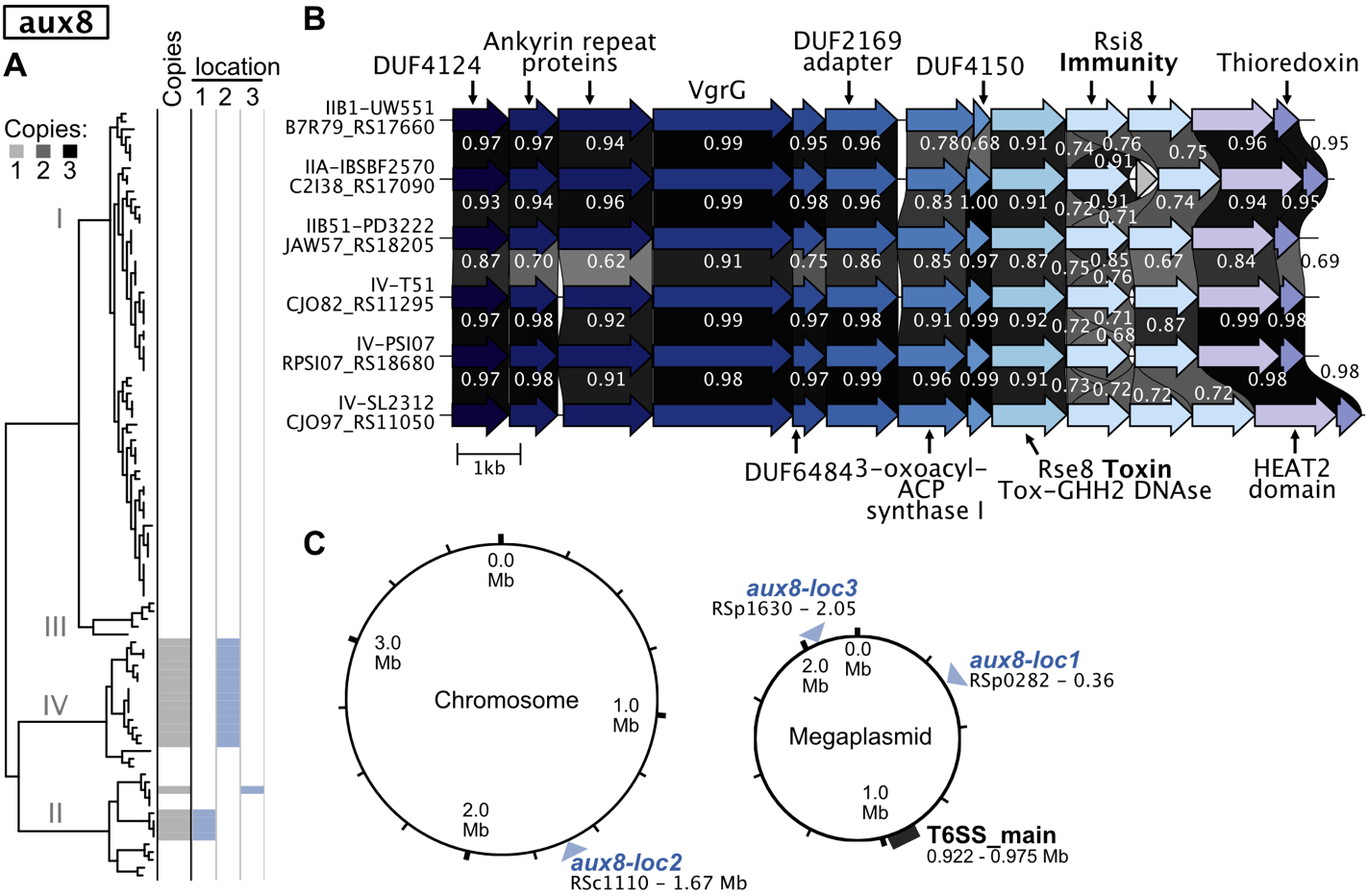


**Figure S13. Phylogenetic distribution, genetic organization/synteny, and chromosomal location of auxiliary *vgrG*-linked cluster 8 (*aux8*).** (**A**) Phylogenetic distribution and copy number of *aux8* across high-quality RSSC genomes. (**B**) Genetic organization/synteny of the cluster from 6 genomes: phyl. IIA IBSBF2570, phyl. IIB-1 UW551, phyl. IIB-51 PD3222, phyl. IV T51, phyl. IV PSI07, and phyl. IV SL2312. *Aux8* encodes a DUF4124 protein, two ankyrin repeat proteins, a VgrG, a DUF6484 protein, a DUF2169 adaptor, a 3-oxoacyl-ACP synthase I, a DUF4150 protein, a toxin with a Tox-GHH2 DNAse domain, two-or-more immunity proteins, a HEAT2 domain protein, and a thioredoxin. Grayscale links indicate the global amino acid identity between homologs. (**C**) *aux8* clusters were identified at three locations across the chromosome and megaplasmid, and these locations are shown relative to the GMI1000 replicons. Panel A indicates which genomes encode the cluster at each location. The figure was generated with a combination of KBase BLASTp, iToL, Clinker, and Affinity Designer.


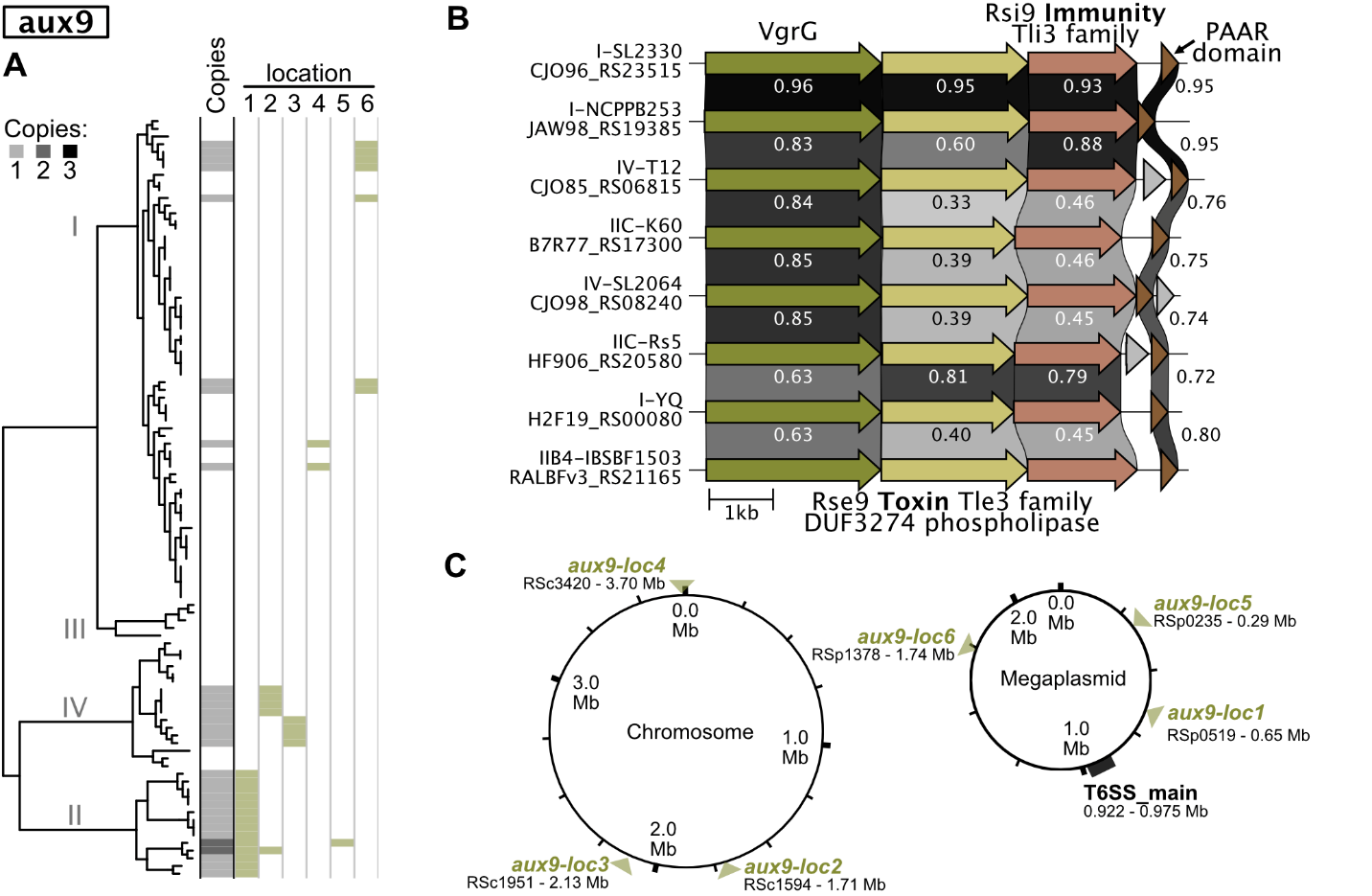


**Figure S14. Phylogenetic distribution, genetic organization/synteny, and chromosomal location of auxiliary *vgrG*-linked cluster 9 (*aux9*).** (**A**) Phylogenetic distribution and copy number of *aux9* across high-quality RSSC genomes. (**B**) Genetic organization/synteny of the cluster from eight genomes: phyl. I SL2330, phyl. I NCPPB253, phyl. I YQ, phyl IIB-4 IBSBF1503, phyl. IIC-7 K60, phyl. IIC-7 Rs5, and phyl. IV SL2064. *Aux9* encodes a VgrG, a Tle3-family phospholipase with a DUF3274 domain, a Tli3 immunity protein(s), a PAAR domain protein, and variably present hypothetical proteins. Grayscale links indicate the global amino acid identity between homologs. (**C**) *aux9* clusters were identified at six locations across the chromosome and megaplasmid, and these locations are shown relative to the GMI1000 replicons. Panel A indicates which genomes encode the cluster at each location. The figure was generated with a combination of KBase BLASTp, iToL, Clinker, and Affinity Designer.


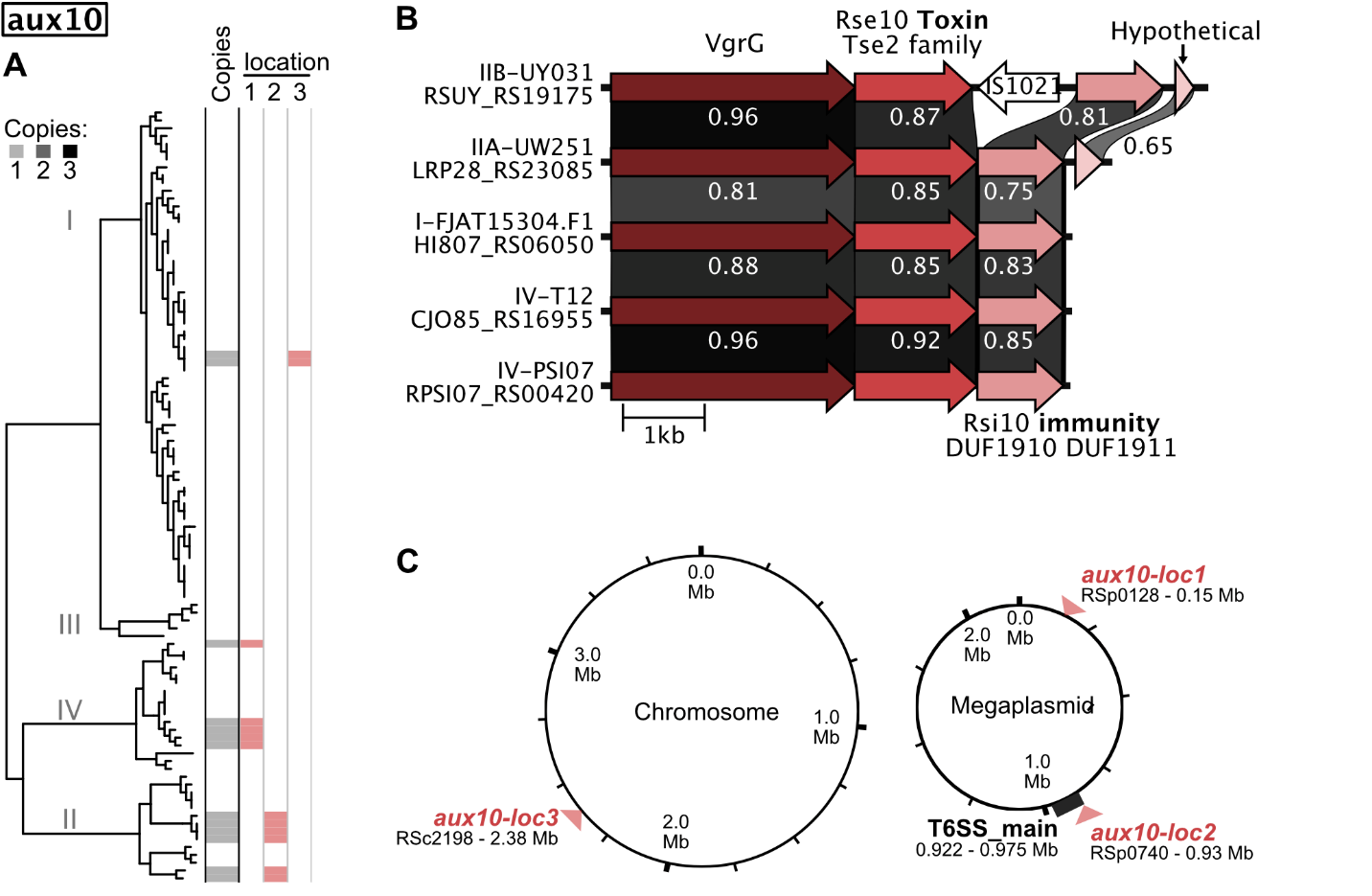


**Figure S15. Phylogenetic distribution, genetic organization/synteny, and chromosomal location of auxiliary *vgrG*-linked cluster 10 (*aux10*).** (**A**) Phylogenetic distribution and copy number of *aux10* across high-quality RSSC genomes. (**B**) Genetic organization/synteny of the cluster from 5 genomes: phyl. I FJAT15304.F1, phyl. IIA UW251, phyl. IIB-1 UY031, phyl. IV T12, and phyl. IV-10 PSI07. *Aux10* encodes a VgrG, a Tse2-family toxin with an unknown target, an immunity protein with DUF1910 and DUF1911 domains, and a variably present hypothetical protein. Grayscale links indicate the global amino acid identity between homologs. (**C**) *aux10* clusters were identified at three locations across the chromosome and megaplasmid, and these locations are shown relative to the GMI1000 replicons. Panel A indicates which genomes encode the cluster at each location. The figure was generated with a combination of KBase BLASTp, iToL, Clinker, and Affinity Designer.


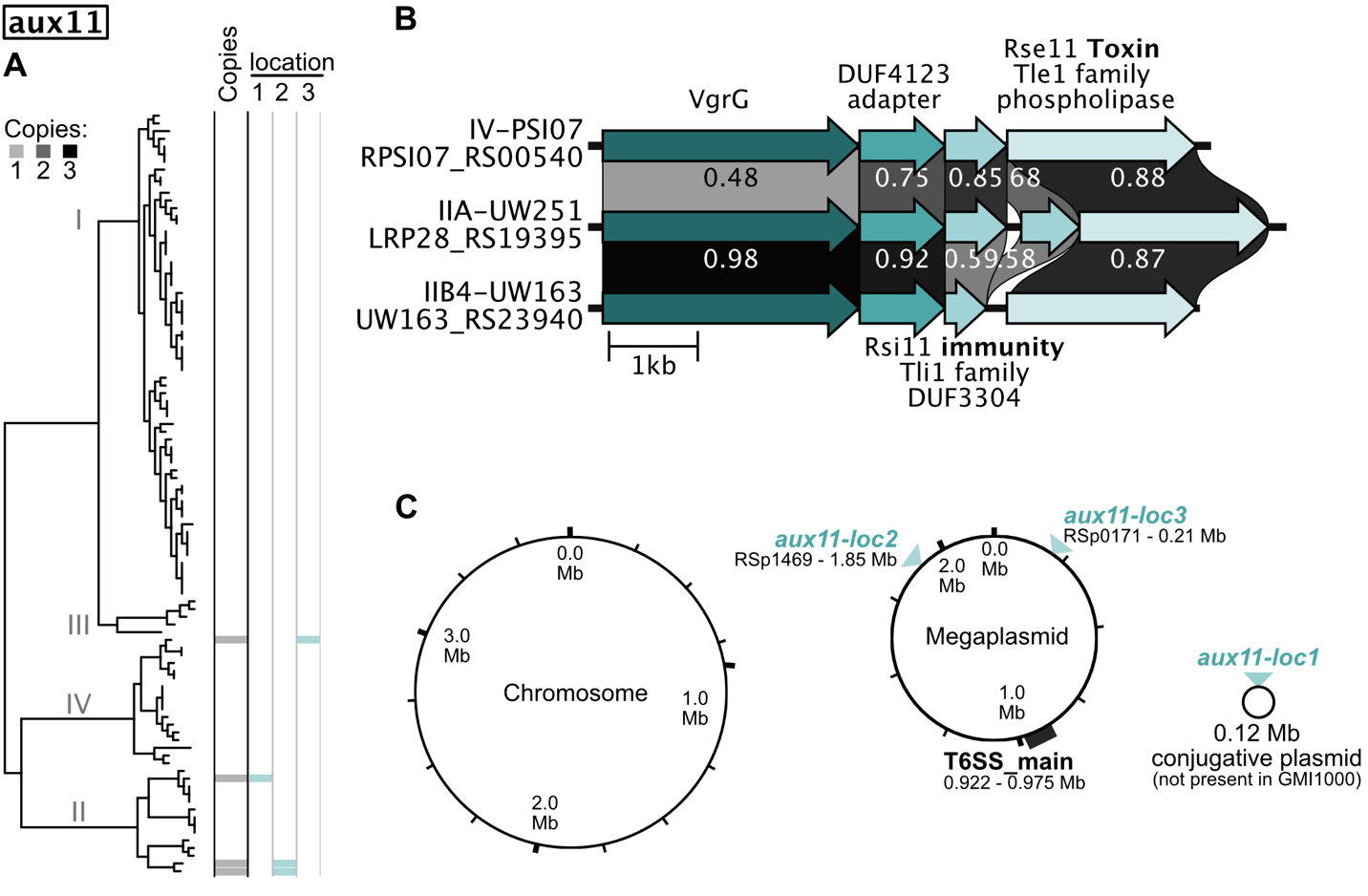


**Figure S16. Phylogenetic distribution, genetic organization/synteny, and chromosomal location of auxiliary *vgrG*-linked cluster 11 (*aux11*).** (**A**) Phylogenetic distribution and copy number of *aux11* across high-quality RSSC genomes. (**B**) Genetic organization/synteny of the cluster from 3 genomes: phyl. IIA UW251, phyl. IIB-4 UW163, and IV PSI07. *Aux11* encodes a VgrG, a DUF4123 adapter, one-or-more Tli1 family immunity proteins with a DUF3304 domain, and a Tle1 family phospholipase toxin. Grayscale links indicate the global amino acid identity between homologs. (**C**) *aux11* clusters were identified at three locations across the chromosome, megaplasmid, and conjugative accessory plasmids, and these locations are shown relative to the GMI1000 replicons. Panel A indicates which genomes encode the cluster at each location. The figure was generated with a combination of KBase BLASTp, iToL, Clinker, and Affinity Designer.


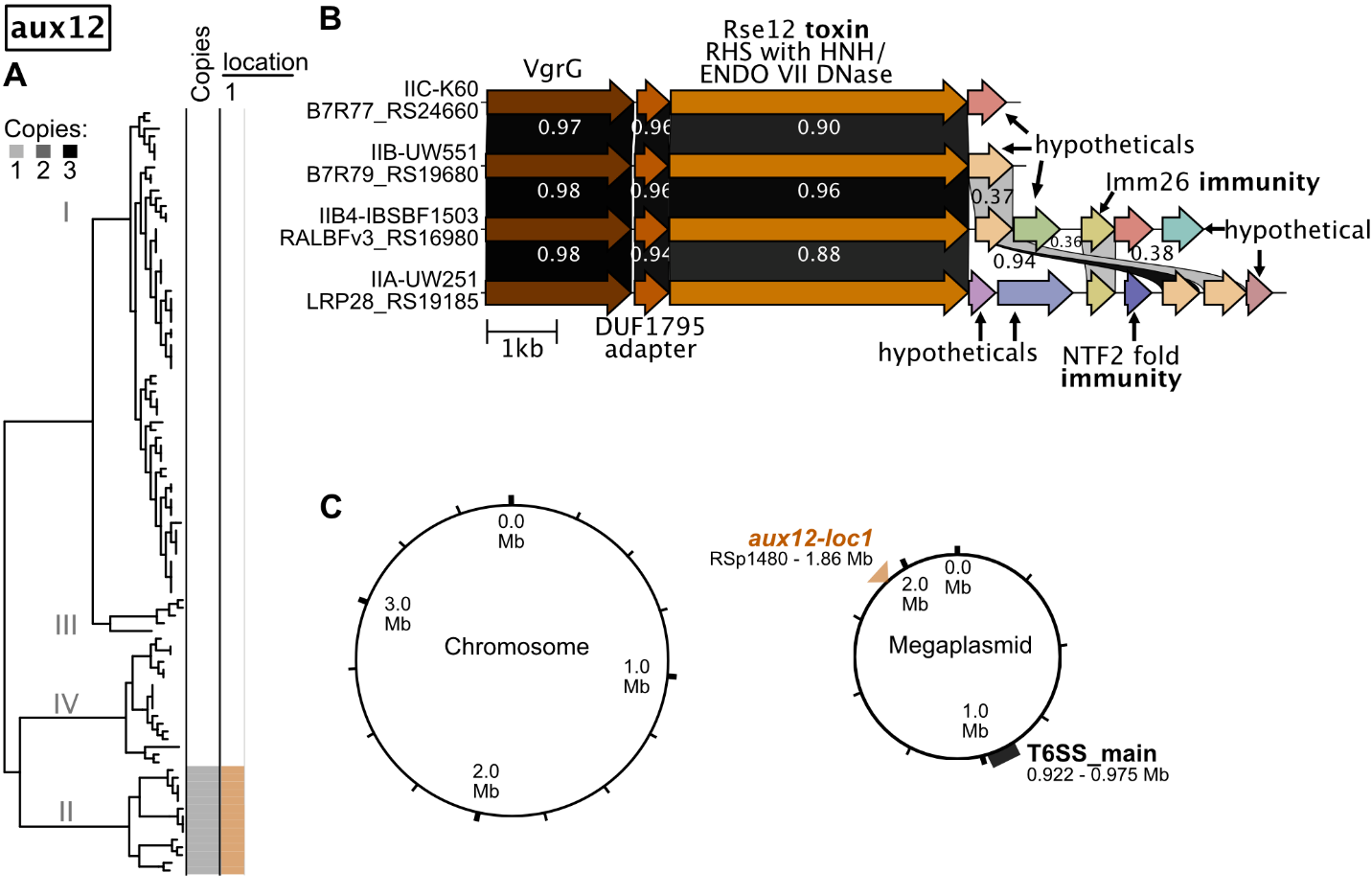


**Figure S17. Phylogenetic distribution, genetic organization/synteny, and chromosomal location of auxiliary *vgrG*-linked cluster 12 (*aux12*).** (**A**) Phylogenetic distribution and copy number of *aux12* across high-quality RSSC genomes. (**B**) Genetic organization/synteny of the cluster from 4 genomes: phyl. IIA UW251, phyl. IIB-1 UW551, phyl. IIB-4 IBSBF1503, and phyl. IIC-7 K60. *Aux12* encodes a VgrG, a DUF1795 adapter, an RHS protein with a C-terminal HNH/ENDO VII DNase toxin, and an array of one-or-more variably present proteins encoding hypotheticals, Imm26 family immunity proteins, and an NFT2-fold immunity protein. The small genes downstream of the RHS toxin likely encode an array of immunity proteins. Grayscale links indicate the global amino acid identity between homologs. (**C**) *aux12* clusters were identified at one location of the megaplasmid. The location is shown relative to the GMI1000 megaplasmid. The figure was generated with a combination of KBase BLASTp, iToL, Clinker, and Affinity Designer.


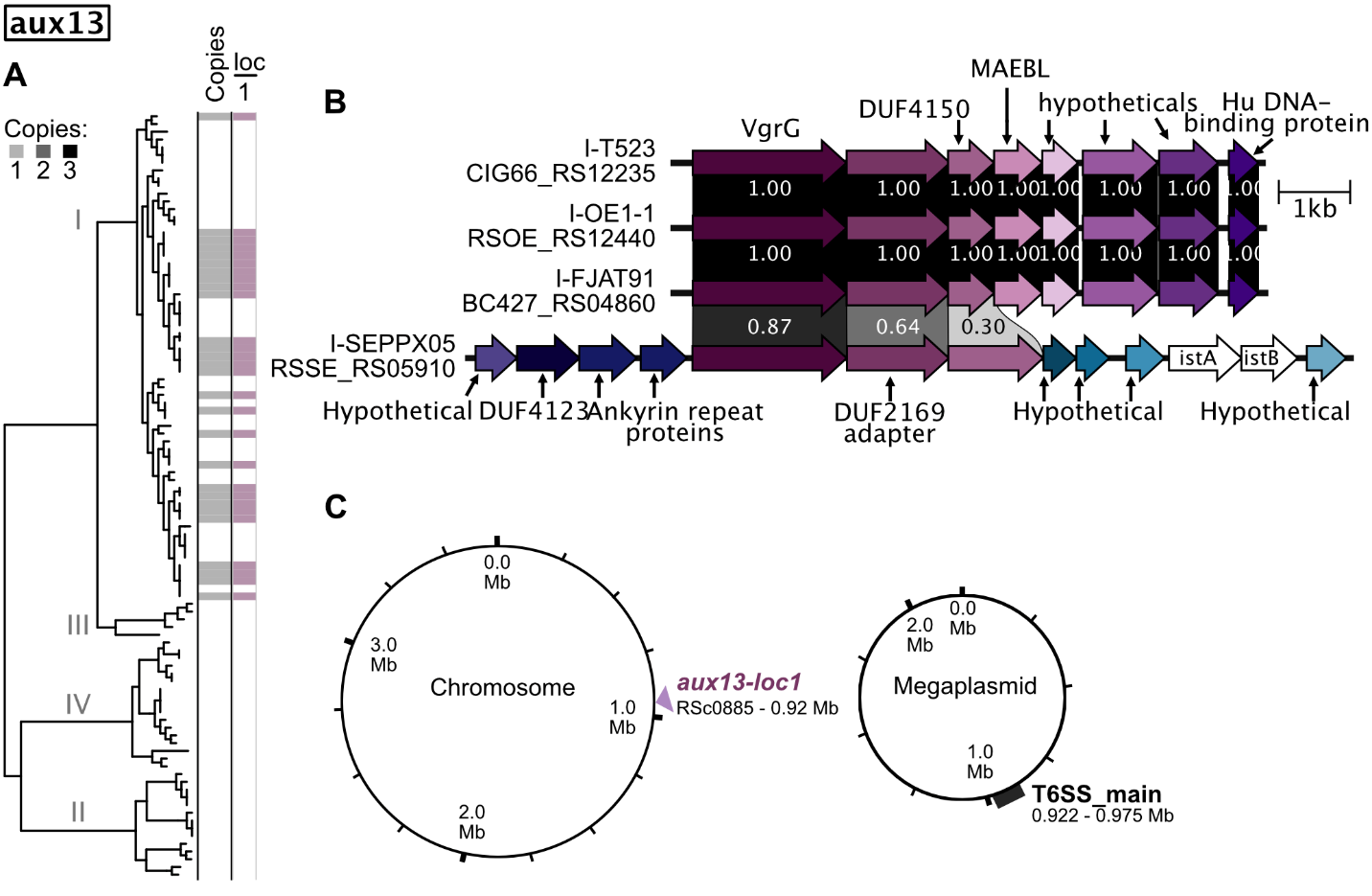


**Figure S18. Phylogenetic distribution, genetic organization/synteny, and chromosomal location of auxiliary *vgrG*-linked cluster 13 (*aux13*).** (**A**) Phylogenetic distribution and copy number of *aux13* across high-quality RSSC genomes. (**B**) Genetic organization/synteny of the cluster from 4 genomes: phyl. I T523, phyl. I OE1-1, phyl. I-FJAT91, and phyl. I SEPPX05. *Aux13* encodes a VgrG, a DUF2169 family adapter, a DUF4150 protein, four hypothetical proteins, and a Hu DNA-binding protein. Grayscale links indicate the global amino acid identity between homologs. (**C**) The *aux13* clusters were identified at one location on the chromosome, and these locations are shown relative to the GMI1000 replicons. Panel A indicates which genomes encode the cluster at each location. The figure was generated with a combination of KBase, BLASTp, iToL, Clinker, and Affinity Designer.


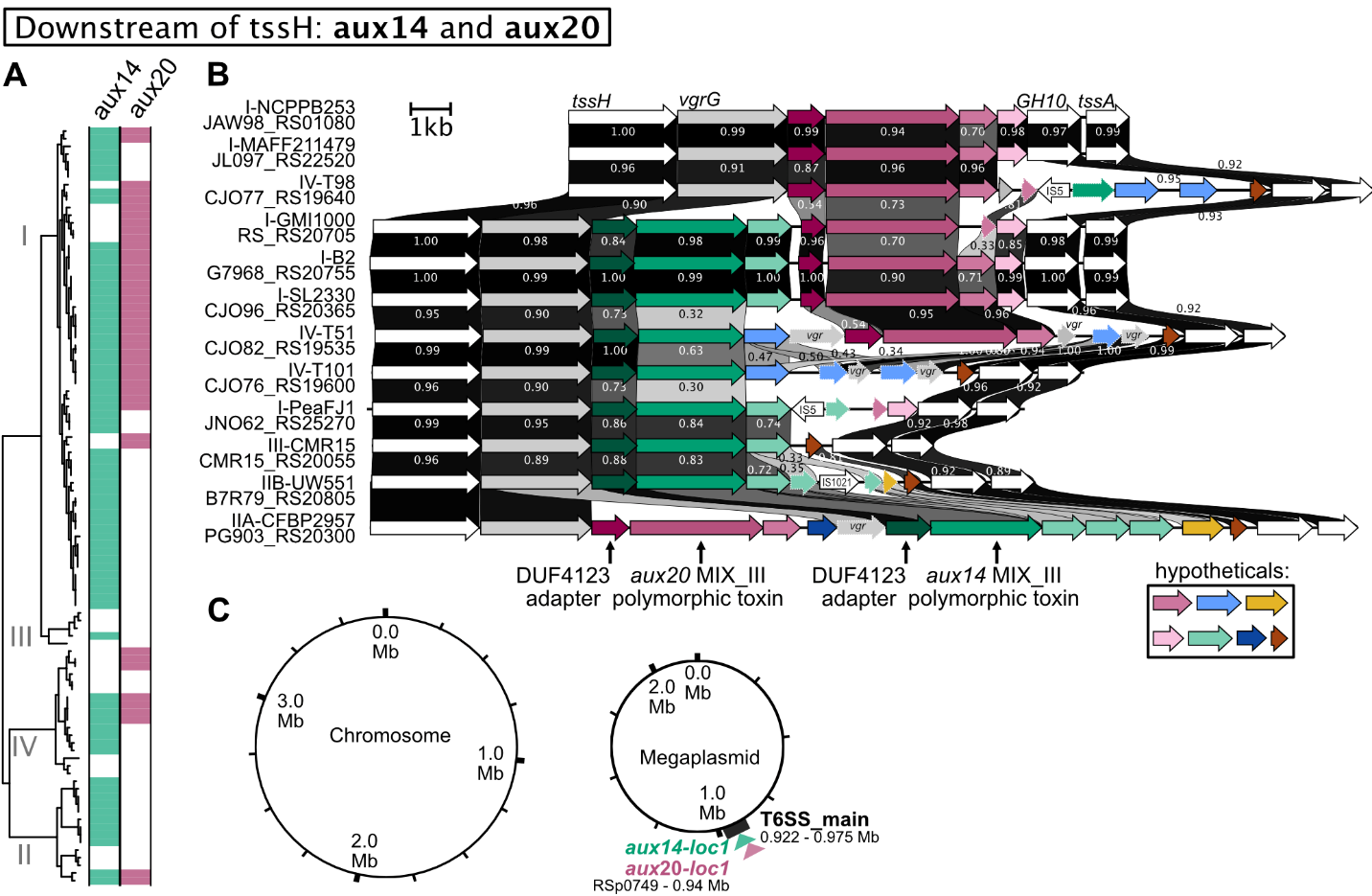


**Figure S19. Phylogenetic distribution, genetic organization/synteny, and chromosomal location of auxiliary *vgrG*-linked clusters located between *tssH* and GH10/*tssA* (*aux14* and *aux20*).** (**A**) Phylogenetic distribution of *aux14* and *aux20* across high-quality RSSC genomes. (**B**) Genetic organization/synteny of four clusters that only contain *aux14*, three clusters that only contain *aux20*, four that contain *aux14* upstream of *aux20*, and one that contains *aux20* upstream of *aux14*. The clusters are from phyl. I NCPPB253, phyl. I MAFF211479, phyl. I GMI1000, phyl. I B2, phyl. I SL2330, phyl. I peaFJ1, phyl. IIA CDBP2957, phyl. IIB1 UW551, phyl. III CMR15, phyl. IV T98, phyl. IV T51, and phyl. IV T101. *Aux14* encodes a VgrG, a DUF4123 adaptor, a MIX_III polymorphic toxin with unknown C-terminal toxin, and a hypothetical protein (presumed immunity). *Aux20* encodes a VgrG, a DUF4123 adaptor, a MIX_III polymorphic toxin with unknown C-terminal toxin, and a hypothetical protein (presumed immunity). (**C**) These clusters are only encoded at the T6SS main locus on the megaplasmid, and this location is shown relative to the GMI1000 replicons. The figure was generated with a combination of KBase BLASTp, iToL, Clinker, and Affinity Designer.


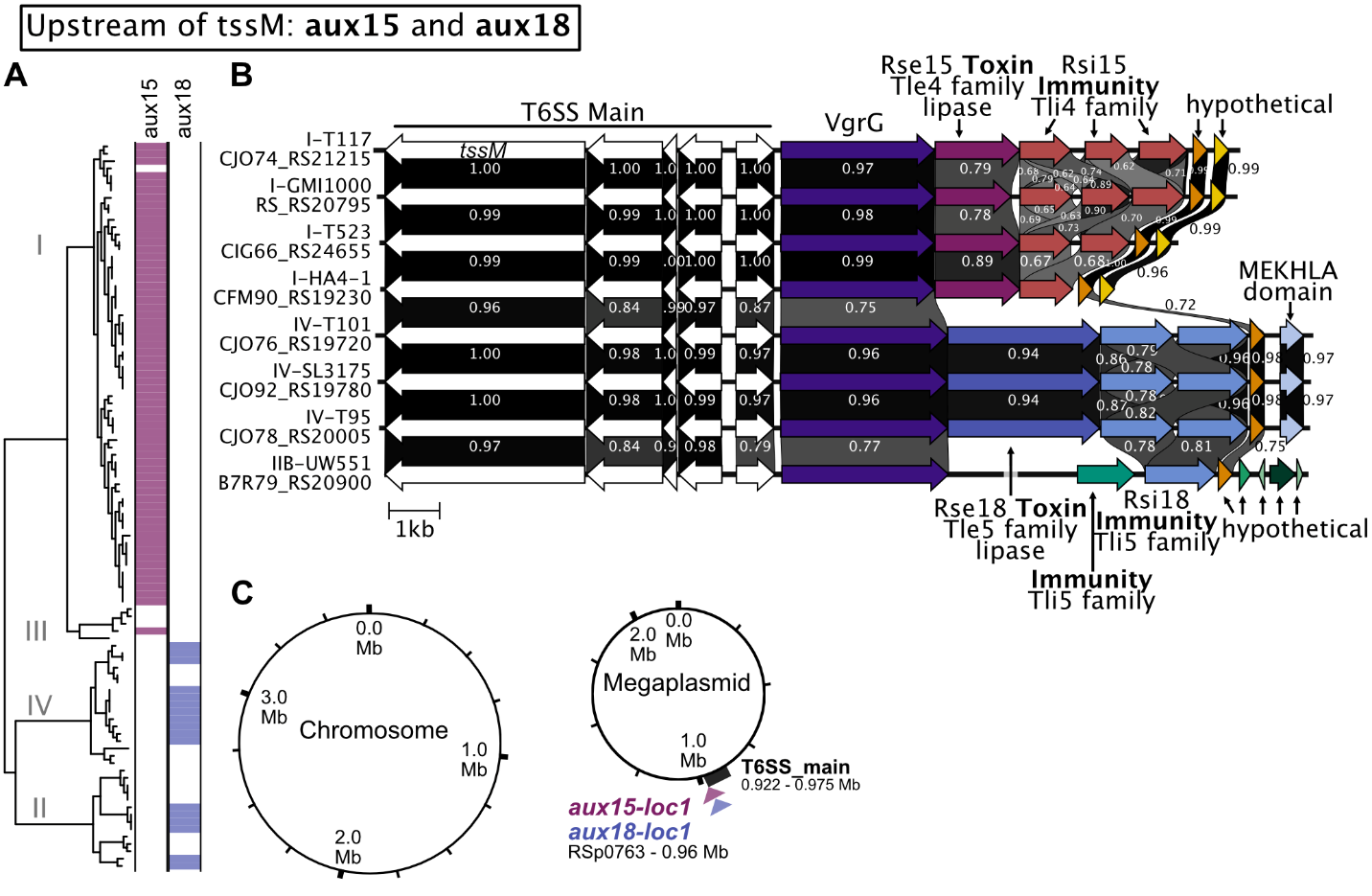


**Figure S20. Phylogenetic distribution, genetic organization/synteny, and chromosomal location of auxiliary *vgrG*-linked clusters located upstream of *tssM* (*aux15* and *aux18*).** (**A**) Phylogenetic distribution of *aux15* and *aux18* across high-quality RSSC genomes. (**B**) Genetic organization/synteny of four *aux15* clusters, three *aux18* clusters, and clusters lacking toxins. The *aux15* clusters are from phyl. I T117, phyl. I GMI1000, phyl. I T523, and phyl. I HA4-1. The *aux18* clusters are from phyl. IV T101, phyl. IV SL3175, and phyl. IV T95. The cluster without a toxin is from phyl. IIB-1 UW551. *Aux15* encodes a VgrG, a Tle4 family phospholipase, one-or-more Tli4 family immunity protein(s), and two hypothetical proteins. *Aux18* encodes a VgrG, a Tle5 phospholipase toxin, two Tli5 immunity proteins, a hypothetical protein that is also encoded in *aux15*, and a MEKHLA domain protein. The IIB-1 cluster encodes two distinct Tli5 immunity proteins, the hypothetical protein from *aux15/aux18* and an array of additional hypothetical proteins. (**C**) These clusters are only encoded at the T6SS main locus on the megaplasmid, and this location is shown relative to the GMI1000 replicons. The figure was generated with a combination of KBase, BLASTp, iToL, Clinker, and Affinity Designer.


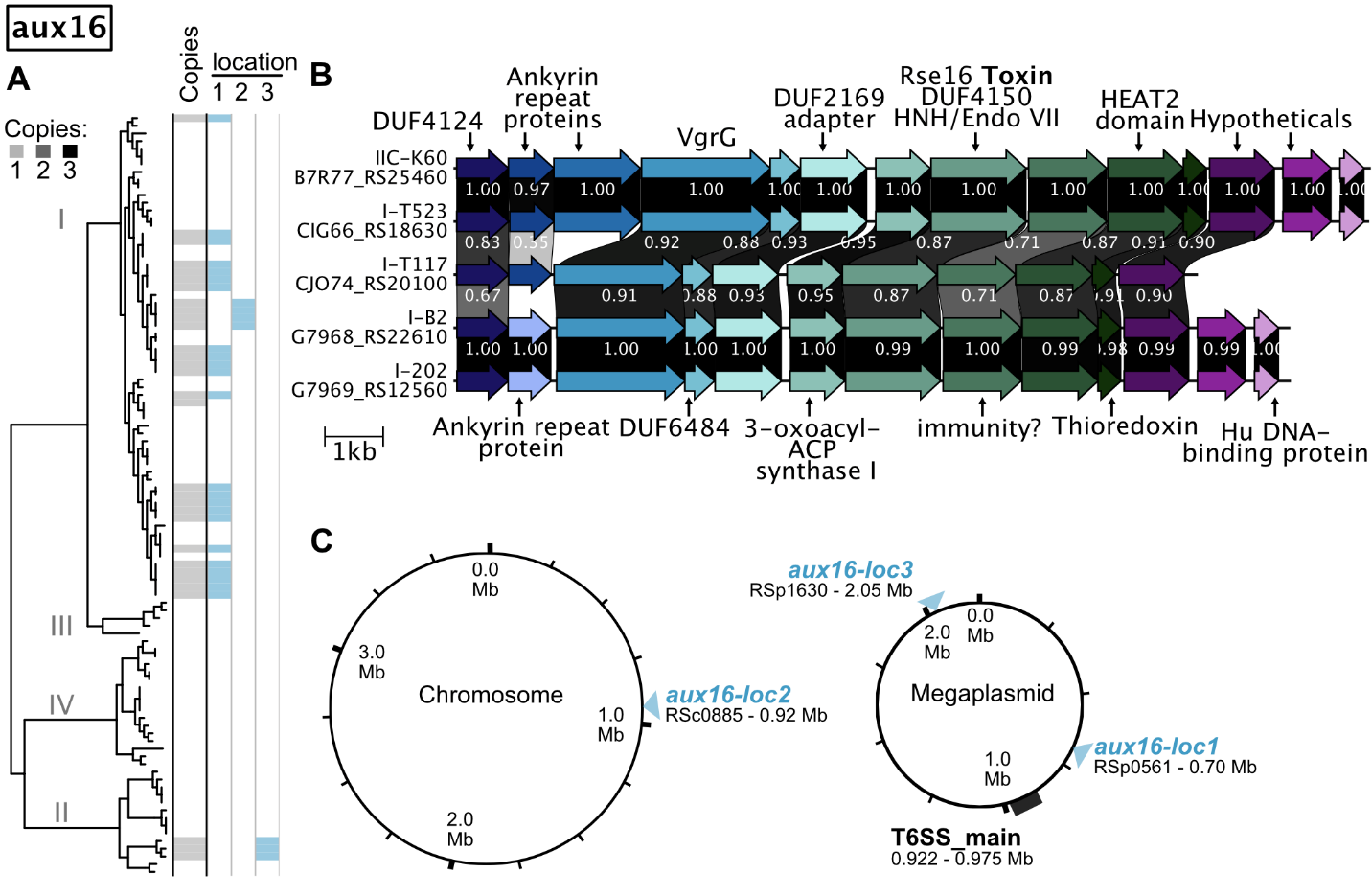


**Figure S21. Phylogenetic distribution, genetic organization/synteny, and chromosomal location of auxiliary *vgrG*-linked cluster 16 (*aux16*).** (**A**) Phylogenetic distribution and copy number of *aux16* across high-quality RSSC genomes. (**B**) Genetic organization/synteny of the cluster from 5 genomes: phyl. I T523, phyl. I T117, phyl. I B2, phyl. I 202 and phyl. IIC-7 K60. *Aux16* encodes a DUF4123 protein, one-to-two ankyrin repeat proteins, a VgrG, a DUF6484 protein, a DUF2169 adaptor, a 3-oxoacyl-ACP synthase I, a DUF4150 protein with a Tox-GHH2 DNAse domain, an immunity protein, a HEAT2 domain protein, thioredoxin, one-or-more hypothetical, and a variably present Hu DNA binding protein. Grayscale links indicate the global amino acid identity between homologs. (**C**) *aux16* clusters were identified at three locations across the chromosome and megaplasmid, and these locations are shown relative to the GMI1000 replicons. Panel A indicates which genomes encode the cluster at each location. The figure was generated with a combination of KBase, BLASTp, iToL, Clinker, and Affinity Designer.


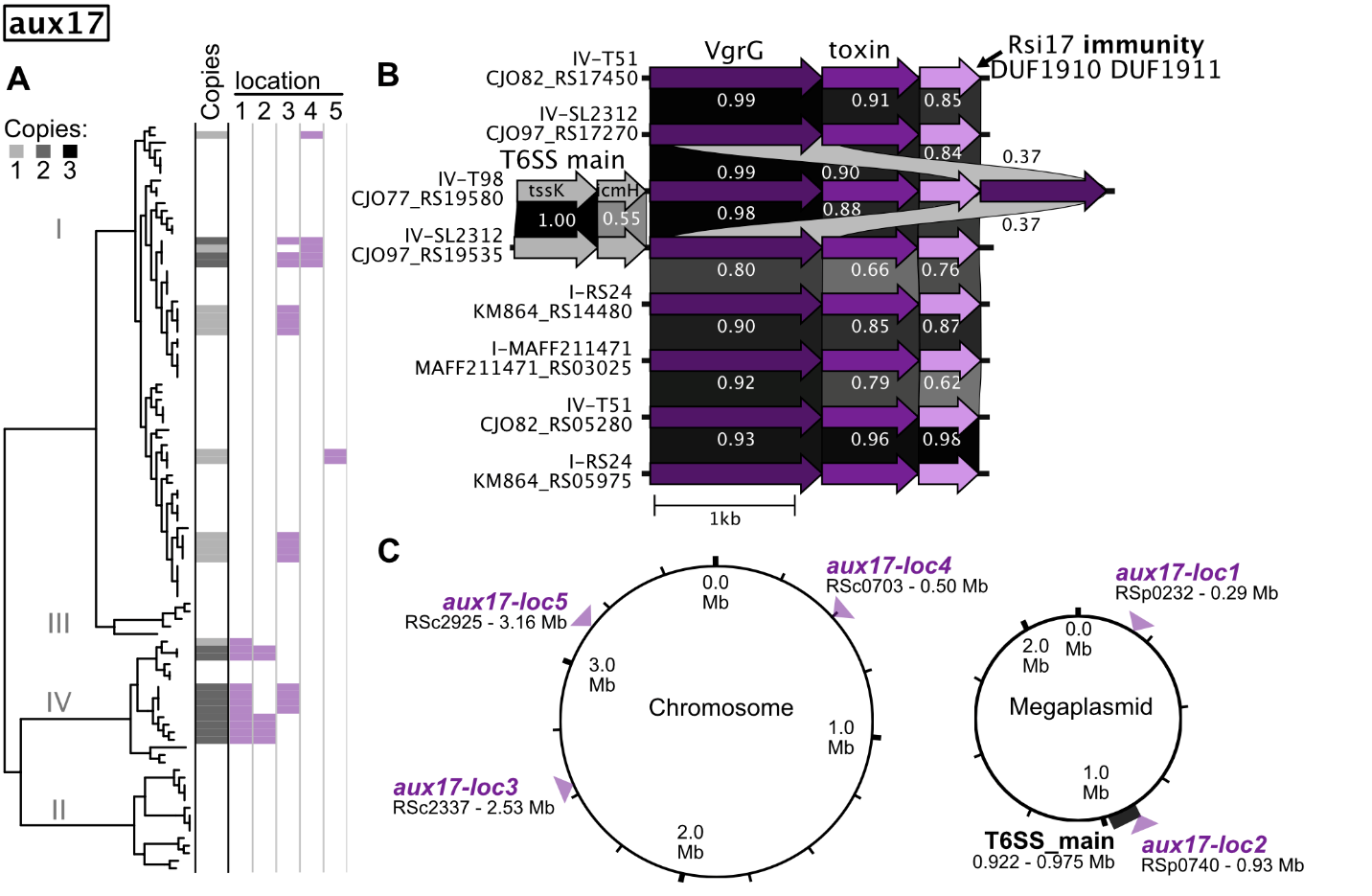


**Figure S22. Phylogenetic distribution, genetic organization/synteny, and chromosomal location of auxiliary *vgrG*-linked cluster 17 (*aux17*).** (**A**) Phylogenetic distribution and copy number of *aux17* across high-quality RSSC genomes. (**B**) Genetic organization/synteny of 8 clusters: phyl. I MAFF211471, two paralogous clusters from phyl. I RS24, two paralogous clusters from phyl. IV T51, two paralogous clusters from phyl. IV SL2312, and phyl. IV T98. *Aux17* encodes a VgrG, a putative toxin that lacks any identified domains, and an immunity protein with DUF1910 and DUF1911 domains. Grayscale links indicate the global amino acid identity between homologs. (**C**) *aux17* clusters were identified at five locations across the chromosome and megaplasmid, and these locations are shown relative to the GMI1000 replicons. Panel A indicates which genomes encode the cluster at each location. The figure was generated with a combination of KBase, BLASTp, iToL, Clinker, and Affinity Designer.


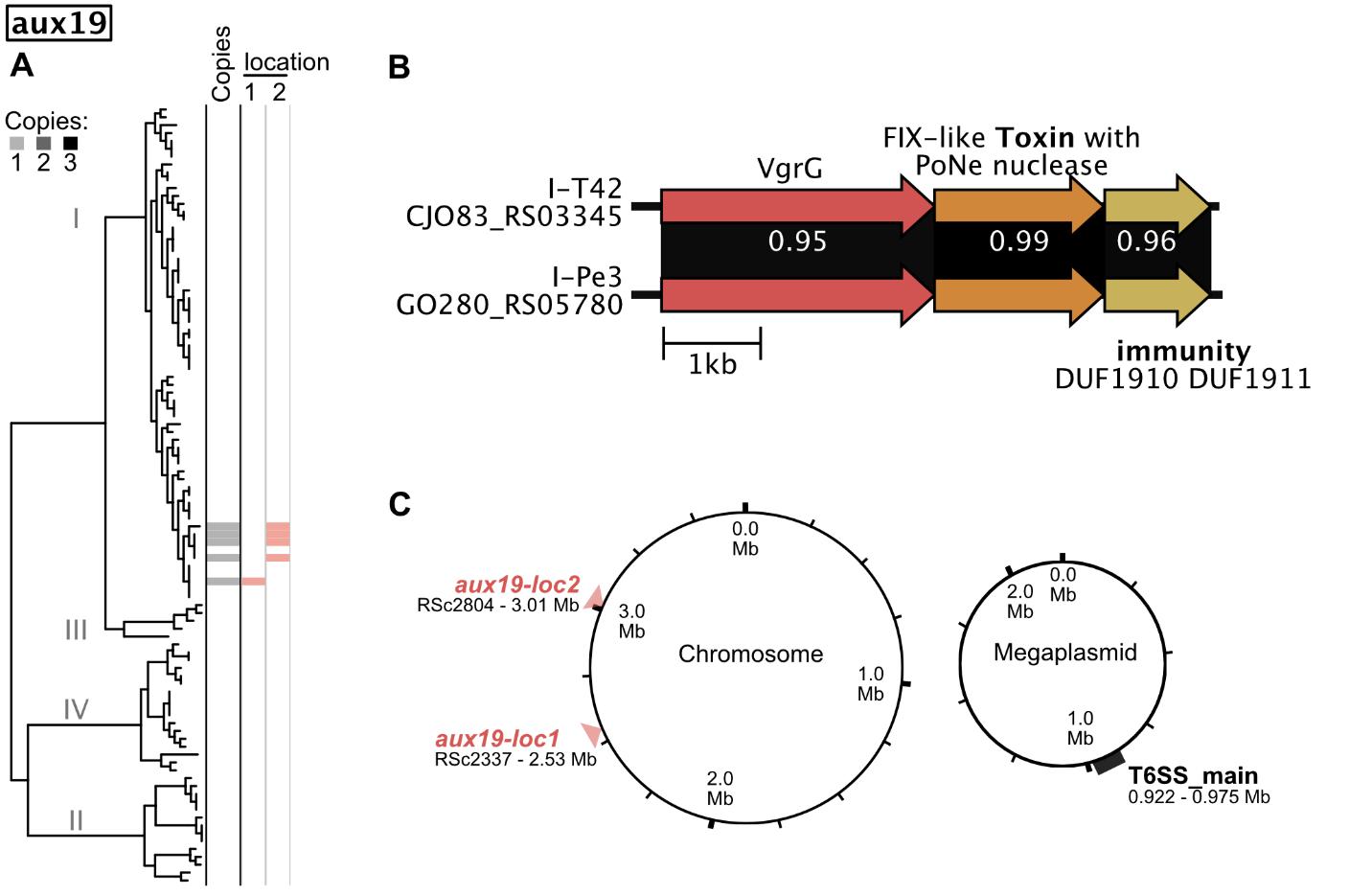


**Figure S23. Phylogenetic distribution, genetic organization/synteny, and chromosomal location of auxiliary *vgrG*-linked cluster 19 (*aux19*).** (**A**) Phylogenetic distribution and copy number of *aux19* across high-quality RSSC genomes. (**B**) Genetic organization/synteny of the cluster from two genomes: phyl I T42, and phyl. I Pe3. *Aux19* encodes a VgrG, a FIX-like polymorphic toxin with a C-terminal PoNe nuclease domain, and an immunity protein with DUF1910 and DUF1911 domains. Grayscale links indicate the global amino acid identity between homologs. (**C**) *aux19* clusters were identified at two locations on the chromosome, and these locations are shown relative to the GMI1000 replicon. Panel A indicates which genomes encode the cluster at each location. The figure was generated with a combination of KBase, BLASTp, iToL, Clinker, and Affinity Designer.


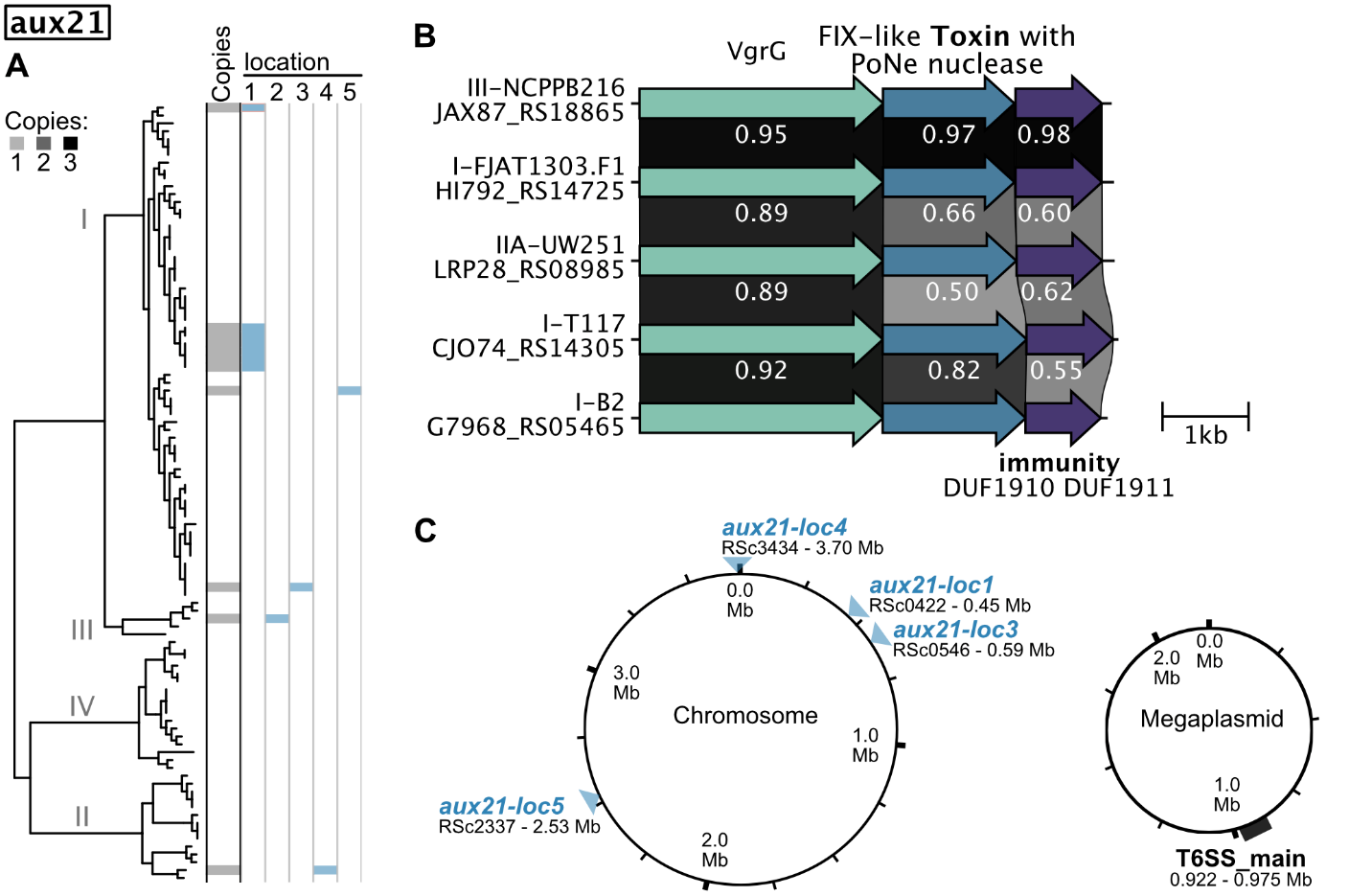


**Figure S24. Phylogenetic distribution, genetic organization/synteny, and chromosomal location of auxiliary *vgrG*-linked cluster 21 (*aux21*).** (**A**) Phylogenetic distribution and copy number of *aux21* across high-quality RSSC genomes. (**B**) Genetic organization/synteny of the cluster from five genomes: phyl. I T117, phyl. I B2, phyl. I FJAT1303.F1, phyl. IIA UW251, and phyl. III NCPPB216. *Aux21* encodes a VgrG, a FIX-like polymorphic toxin with a SEN1 helicase domain and a C-terminal PoNe nuclease domain, and a DUF1910/DUF1911 immunity protein. Grayscale links indicate the global amino acid identity between homologs. (**C**) *aux21* clusters were identified at five locations across the chromosome, and these locations are shown relative to the GMI1000 replicon. Panel A indicates which genomes encode the cluster at each location. The figure was generated with a combination of KBase, BLASTp, iToL, Clinker, and Affinity Designer.


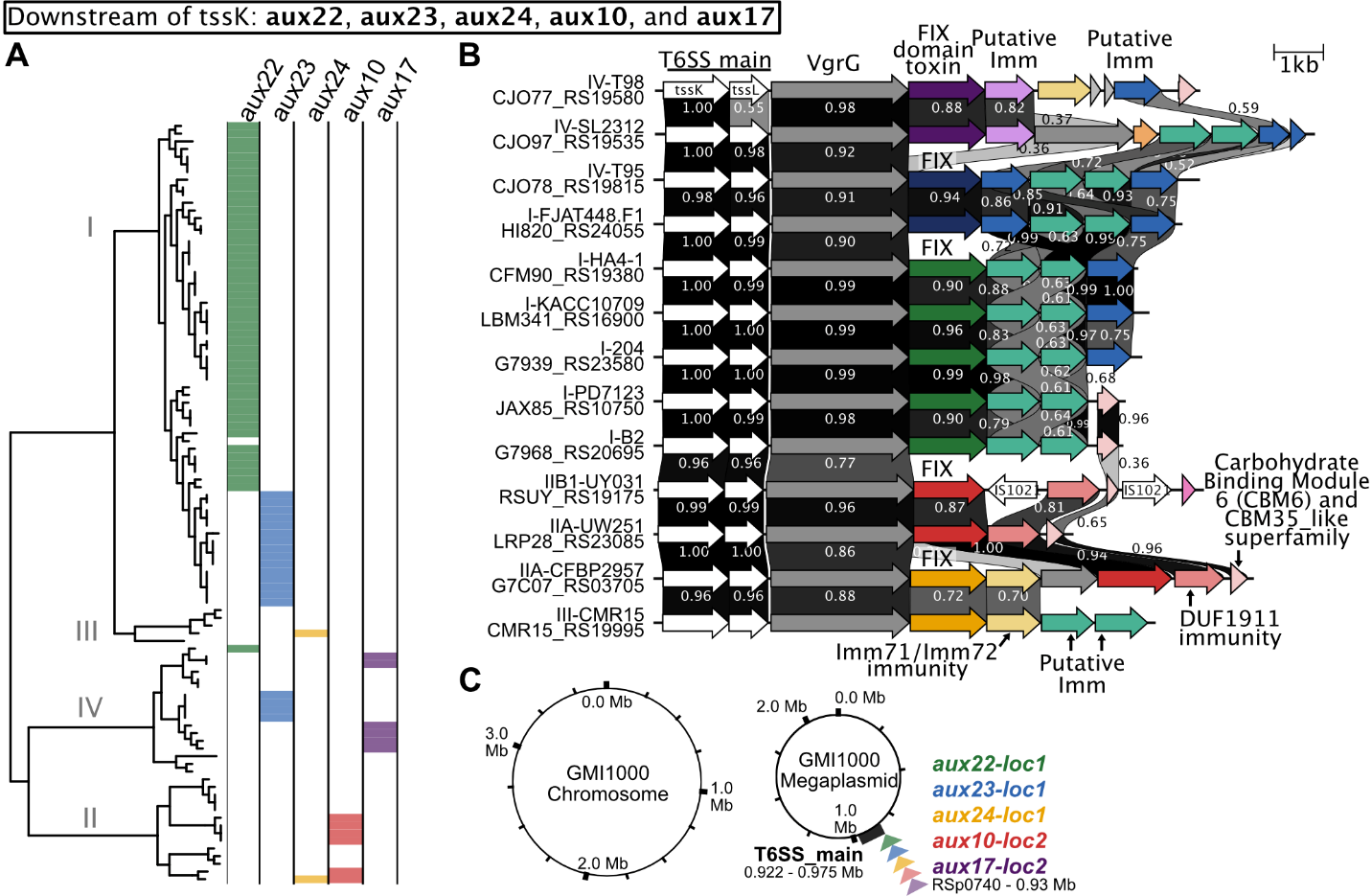


**Figure S25. Phylogenetic distribution, genetic organization/synteny, and chromosomal location of auxiliary *vgrG*-linked clusters that are downstream of *tssJKL* (*aux22, aux 23, aux24, aux10,* and *aux17*).** (**A**) Phylogenetic distribution of each cluster across high-quality RSSC genomes. (**B**) Genetic organization/synteny of 13 clusters. All clusters encode a VgrG, one of five distinct FIX domain toxins, and one or more putative immunity proteins. *Aux10* is represented by phyl. IIB-1 UY031 and phyl. IIA UW251. *Aux17* is represented by phyl. IV T98 and phyl. IV SL2312. *Aux22* is represented by phyl. I strains HA4-1, 204, PD7123, and B2. *Aux23* is represented by phyl. IV T95 and phyl. I FJAT448.F1. *Aux24* is represented by phyl. IIA CFBP2957 and phyl. III CMR15. Grayscale links indicate the global amino acid identity between homologs. (**C**) Three of these clusters (*aux22*, *aux23*, and *aux24*) are encoded exclusively at the T6SS main locus on the megaplasmid. *Aux10* and *aux17* can be found at the T6SS main locus or at the other location indicated on figures S13 and S27 that focus on each of these clusters. The figure was generated with a combination of KBase, BLASTp, iToL, Clinker, and Affinity Designer.


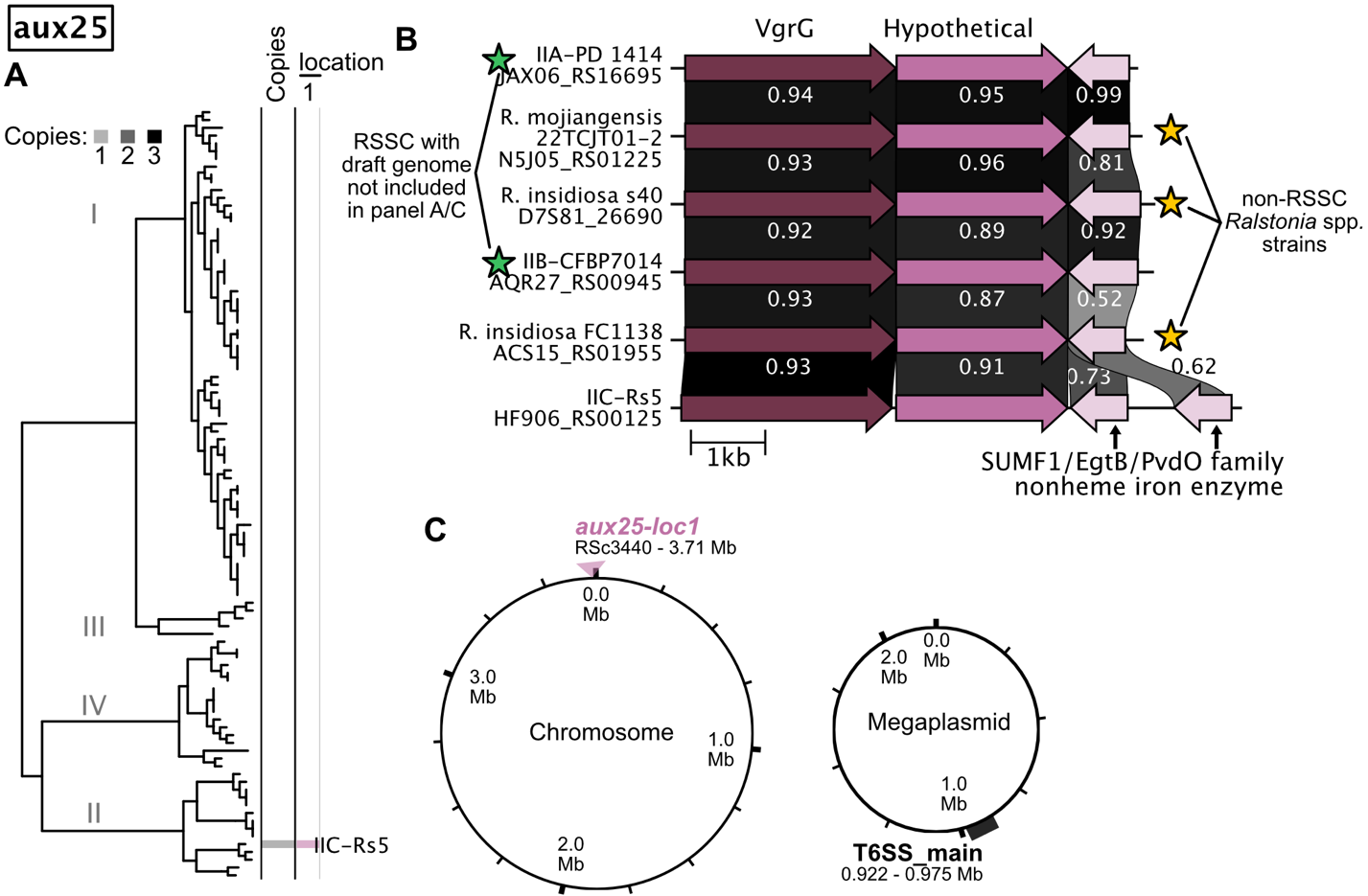


**Figure S26. Phylogenetic distribution, genetic organization/synteny, and chromosomal location of the atypical, auxiliary *vgrG*-linked gene cluster *aux25*.** (**A**) Phylogenetic distribution of *aux25* across high-quality RSSC genomes. Not shown: *aux25* is also encoded in the draft genomes of IIA strain PD1414 and IIB-51 strain CFBP7014. (**B**) Genetic organization/synteny of *aux25* from RSSC strains (IIC Rs5, IIA PD1414, and IIB-51 CFBP7014) and strains in other *Ralstonia* species (FC1138, 22TCJT01-2, and s40). All clusters encode a VgrG, a hypothetical protein, and a SUMF1/EgtB/PvdO family nonheme iron enzyme. The SUMF1 gene is encoded on the opposing strand of the rest of the cluster, which is atypical. Grayscale links indicate the global amino acid identity between homologs. (**C**) The relative location of *aux25 in Rs5* is shown relative to the GMI1000 chromosome. The figure was generated with a combination of KBase, BLASTp, iToL, Clinker, and Affinity Designer.


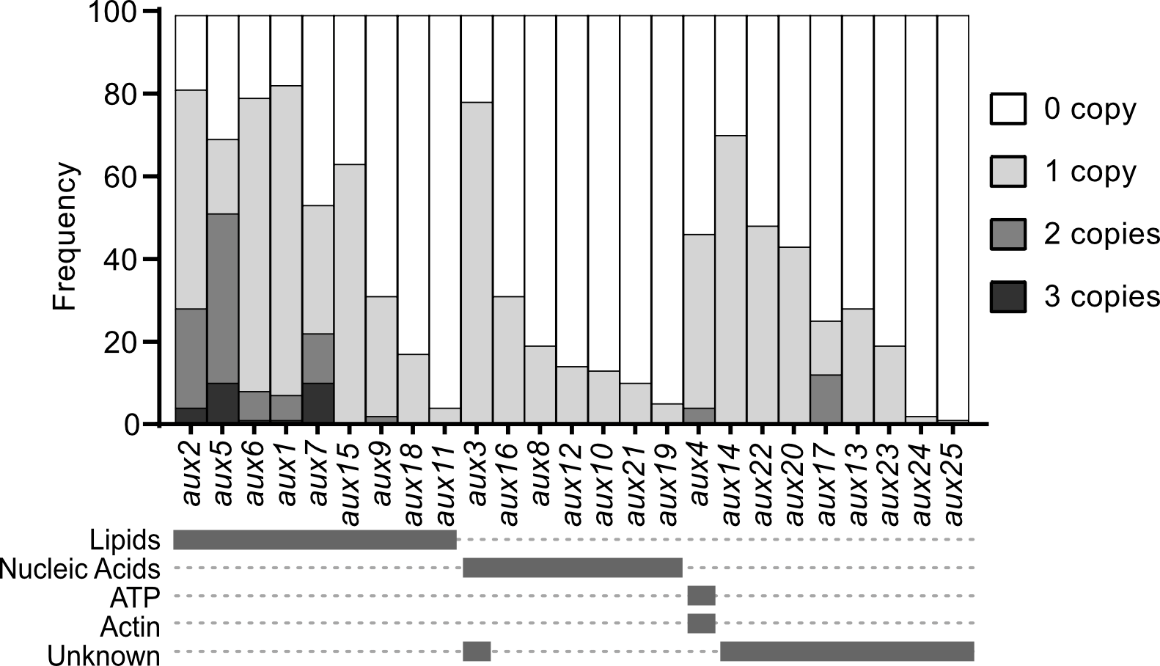


**Figure S27. RSSC genomes encode 0-3 copies of *aux* clusters.** *Aux* clusters are organized by the substrate that the toxin targets.

­­­


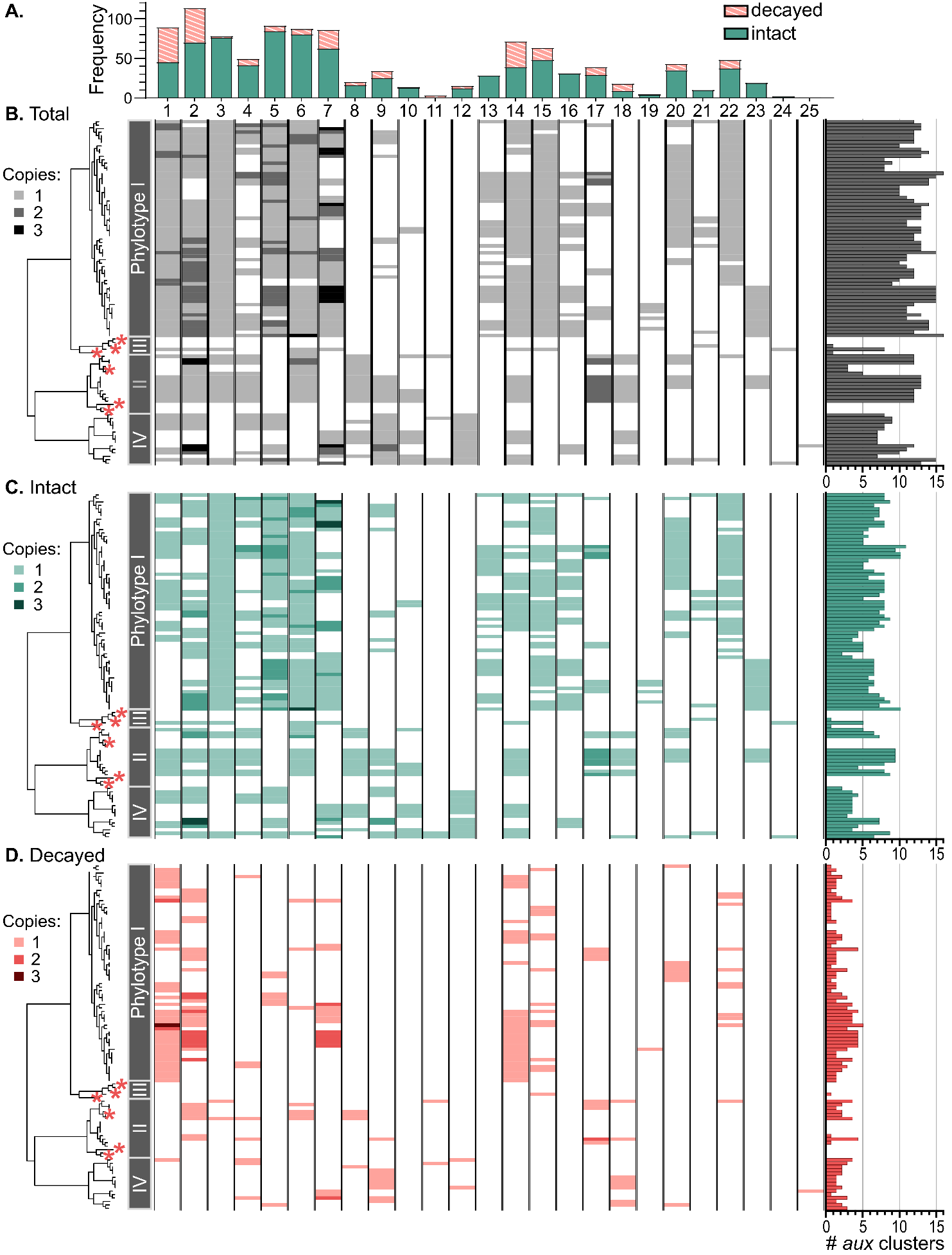
**Figure S28. RSSC strains vary in their repertoires of *vgrG*-linked auxiliary toxin/immunity clusters.** The presence of each cluster (*aux1-aux25*) was determined across complete or nearly complete RSSC genomes by a combination of BLASTp and Clinker analyses and visualized with iToL. (**A**) shows the frequency of each cluster and the proportion that are intact (genes lack any obvious loss-of-function mutations) or decayed (one-or-more genes have loss-of-function mutations like pseudogenization/frameshifts, deletions that truncate or remove a gene, or transposon insertions into a gene). (**B**) shows the total prevalence of *aux* clusters in each genome, (**C**) shows the prevalence of intact clusters, and (**D**) shows the prevalence of decayed clusters. The phylogenetic tree was constructed with the KBase “Species Tree” app. The bar plot on the right shows the number of *aux* clusters in each genome. Genomes that lack a T6SS are indicated by red asterisks to the right of the branch.


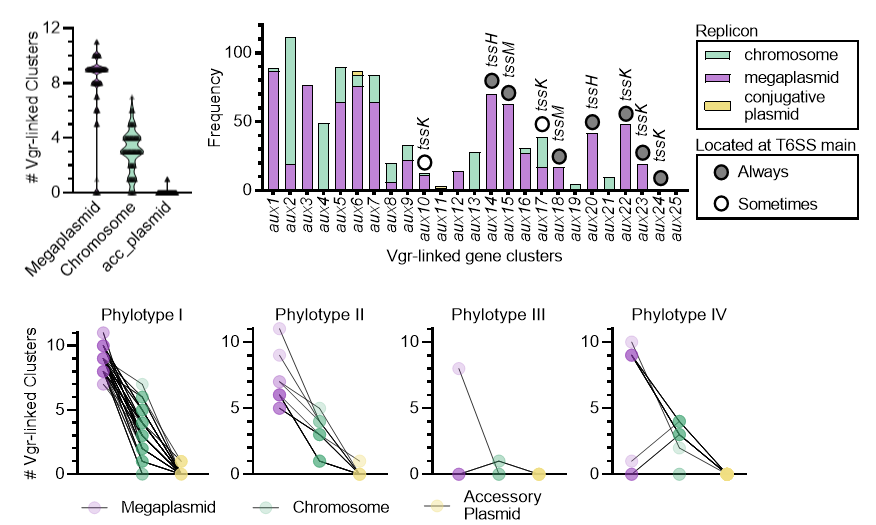


## Figure S29. *Aux* clusters are enriched on the megaplasmid for all T6SS*^+^* RSSC strains. Each genome is represented by a line that connects semi-transparent circles. Circle opacity corresponds to the number of genomes represented by the circle. *Aux* clusters are more enriched on the megaplasmid than on the chromosome and accessory plasmid in most T6SS^+^ genomes.


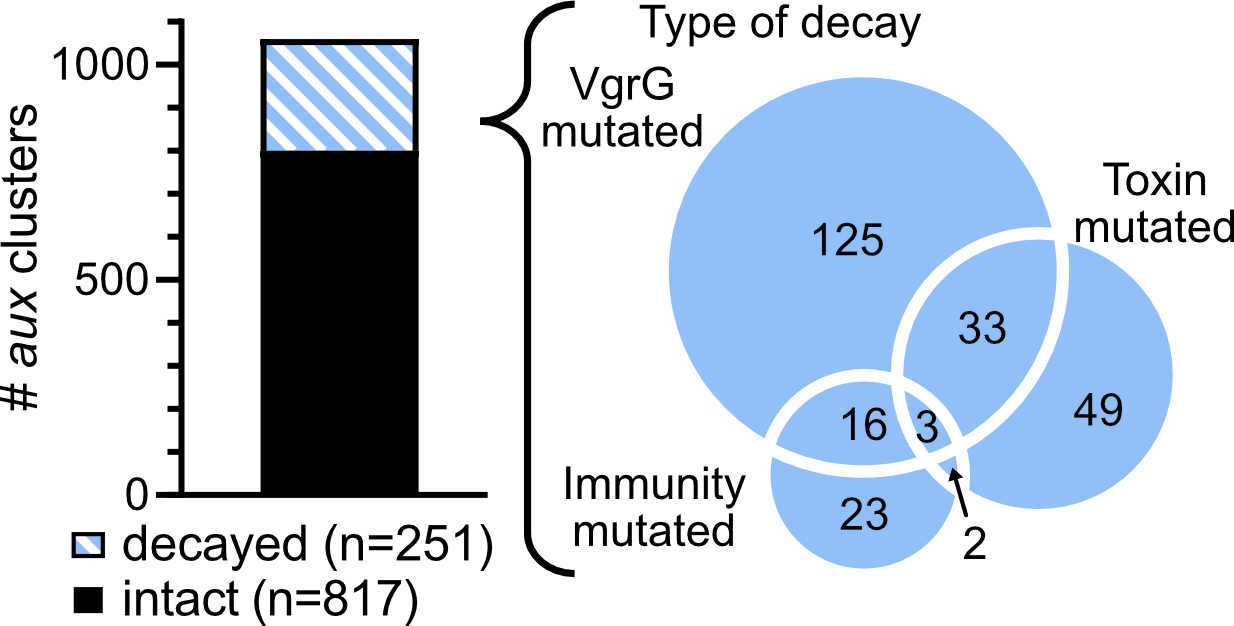


**Figure S30. Patterns of loss-of-function mutations observed in *aux* clusters.** Evidence of gene decay/loss was investigated for each of the 1066 *aux* clusters. Frameshift and other pseudogenization mutations were identified based on NCBI RefSeq annotations. Genetic fragmentation, disruption by transposon insertion, and gene loss were identified through Clinker analysis of synteny. BioVenn (doi.org/10.1186/1471-2164-9-488) was used to create a proportional Venn diagram to reflect whether mutations occurred in the *vgr,* toxin, or immunity gene.


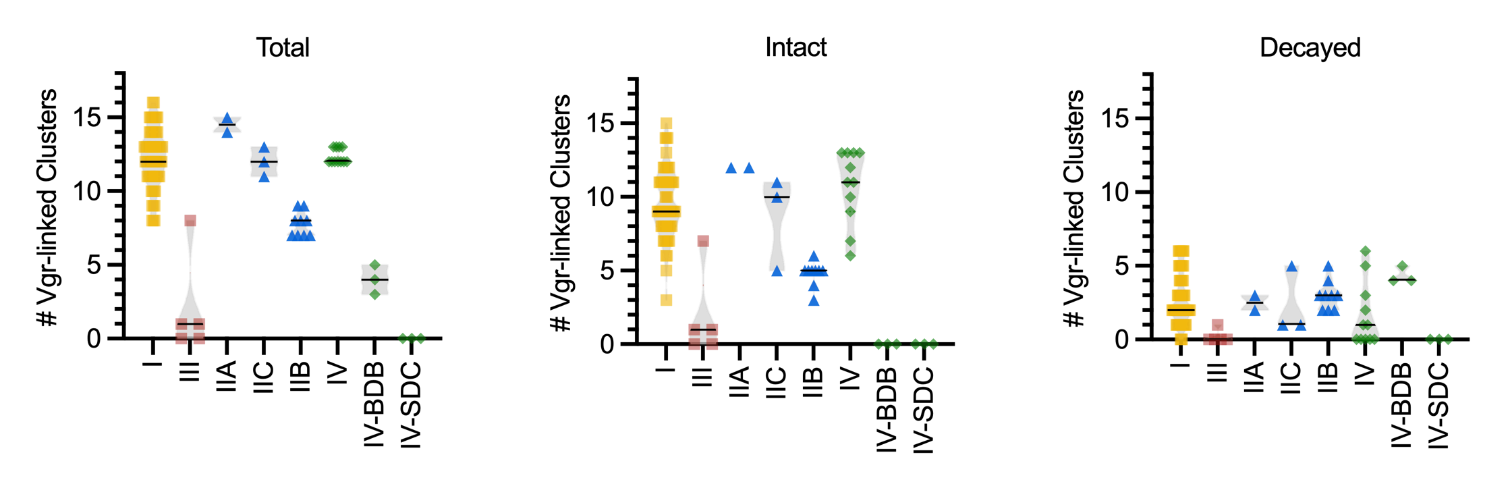


**Figure S31. Comparison of total, intact, and decayed *aux* clusters amongst RSSC clades.** *Aux* clusters were identified in complete or nearly complete genomes by BLAST searches for genes encoding VgrG, toxins, or immunity proteins. The *aux* clusters were classified by synteny analysis with Clinker. RSSC clades are colored by species: yellow, *R. pseudosolanacearum* phylotype I and III; blue, *R. solanacearum* phylotype IIA, IIB, and IIC; green, *R. syzygii* phylotype IV strains with the soil-borne strains (“IV”) separated from the Blood Disease of Banana strains (“IV-BDB”) and Sumatra Disease of Clove strains (“IV-SDC”).


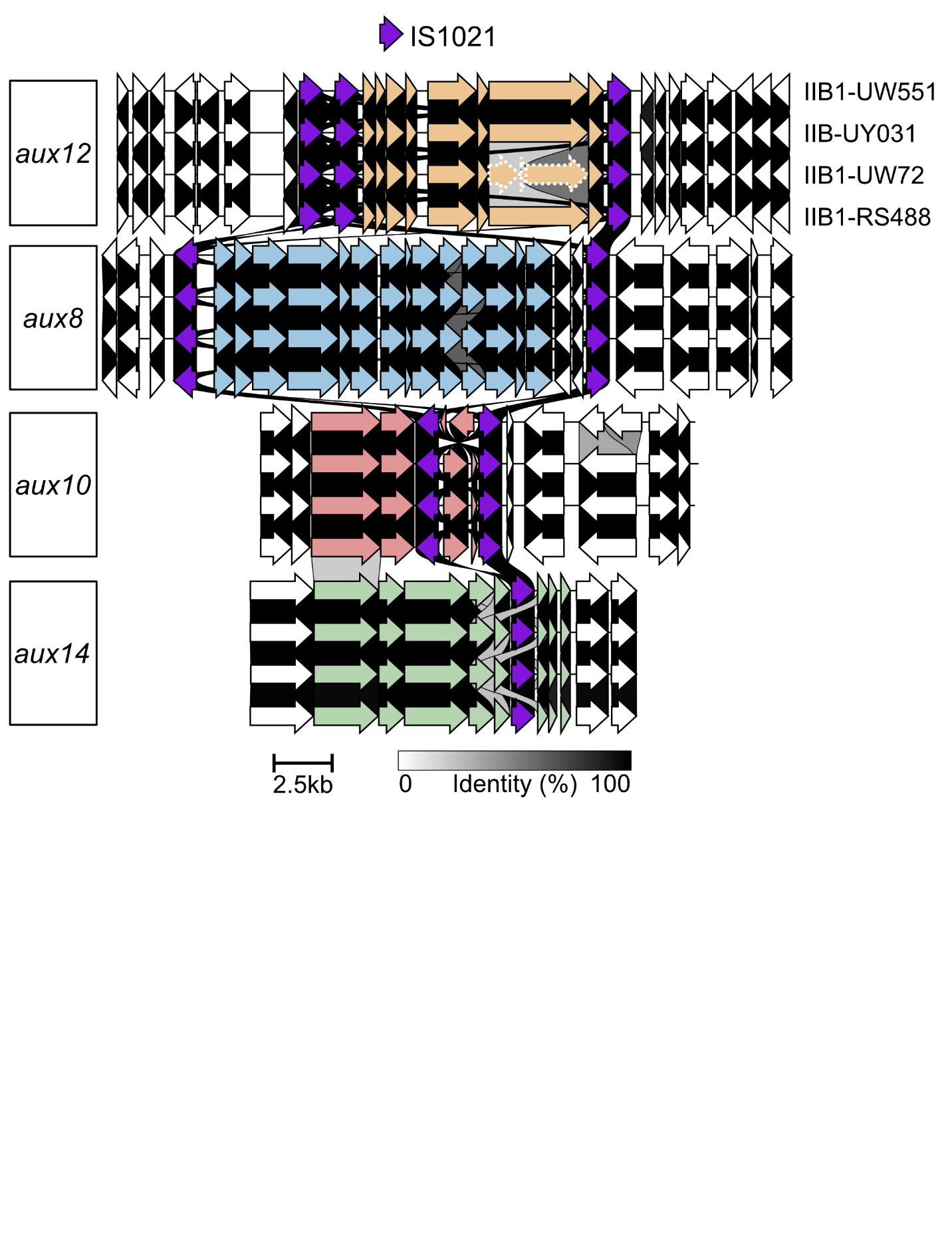


**Figure S32. *Aux* clusters in the pandemic brown rot lineage (IIB-1) are associated with IS1021 elements.** Synteny was visualized with Clinker. IS1021 elements are colored purple. The presence of IS1021 elements on both ends of *aux8* and *aux12* suggests that these clusters are encoded on composite transposons.


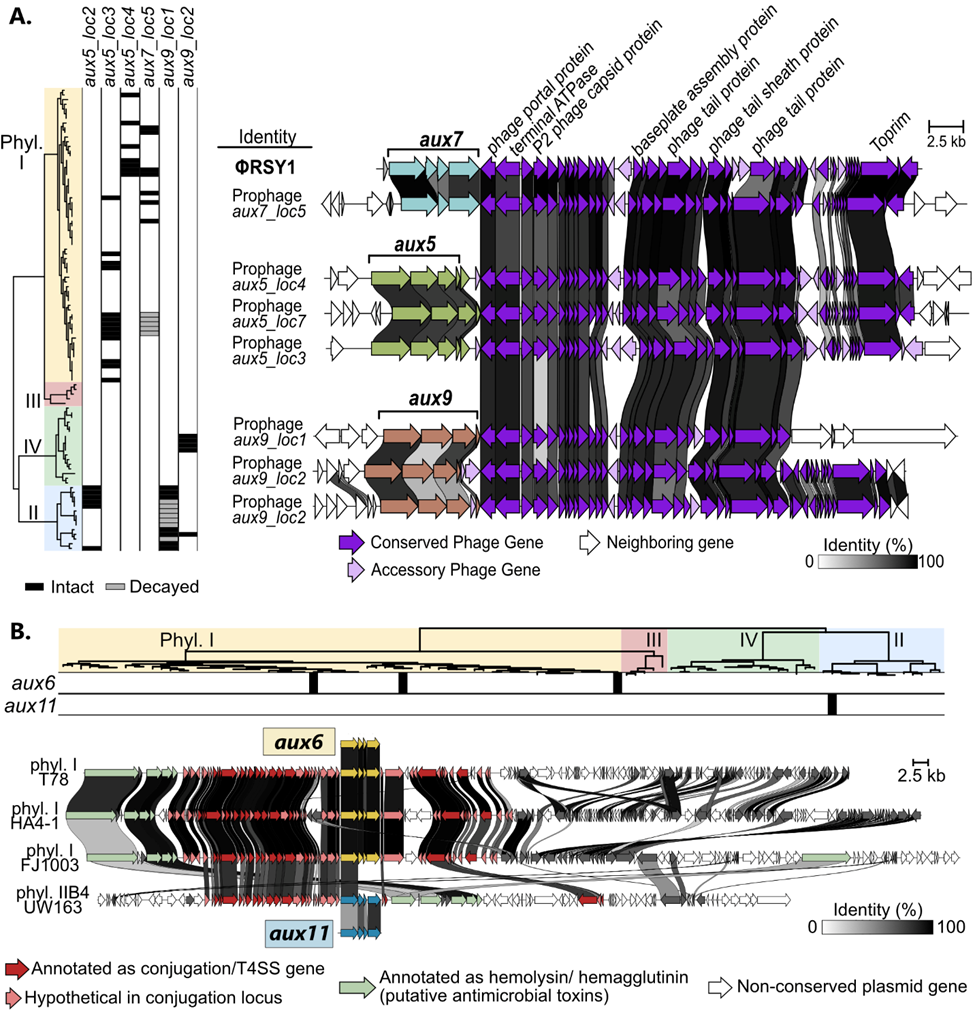


## Figure S33. Mobile genetic elements contribute to horizontal gene transfer of *vgrG*-linked toxin clusters. (A) *aux5*, *aux7*, and *aux9* are sometimes encoded within RSY1-like bacteriophages. These *aux*-encoding phages insert into 7 different locations and the phylogenetic distribution of each phage-location pair is shown on the left. If the *aux* cluster had clear mutations, the location was marked as decayed (gray). Otherwise, the location is marked as intact (black). Synteny of the phages was visualized with Clinker (right) (B) *aux6* and *aux11* are sometimes located on rare, conjugative plasmids. Phylogenetic distribution of *aux-*encoding conjugative plasmids is shown on top. Synteny of the full-length plasmids was visualized with Clinker and genes with putative conjugative functions are highlighted in red.


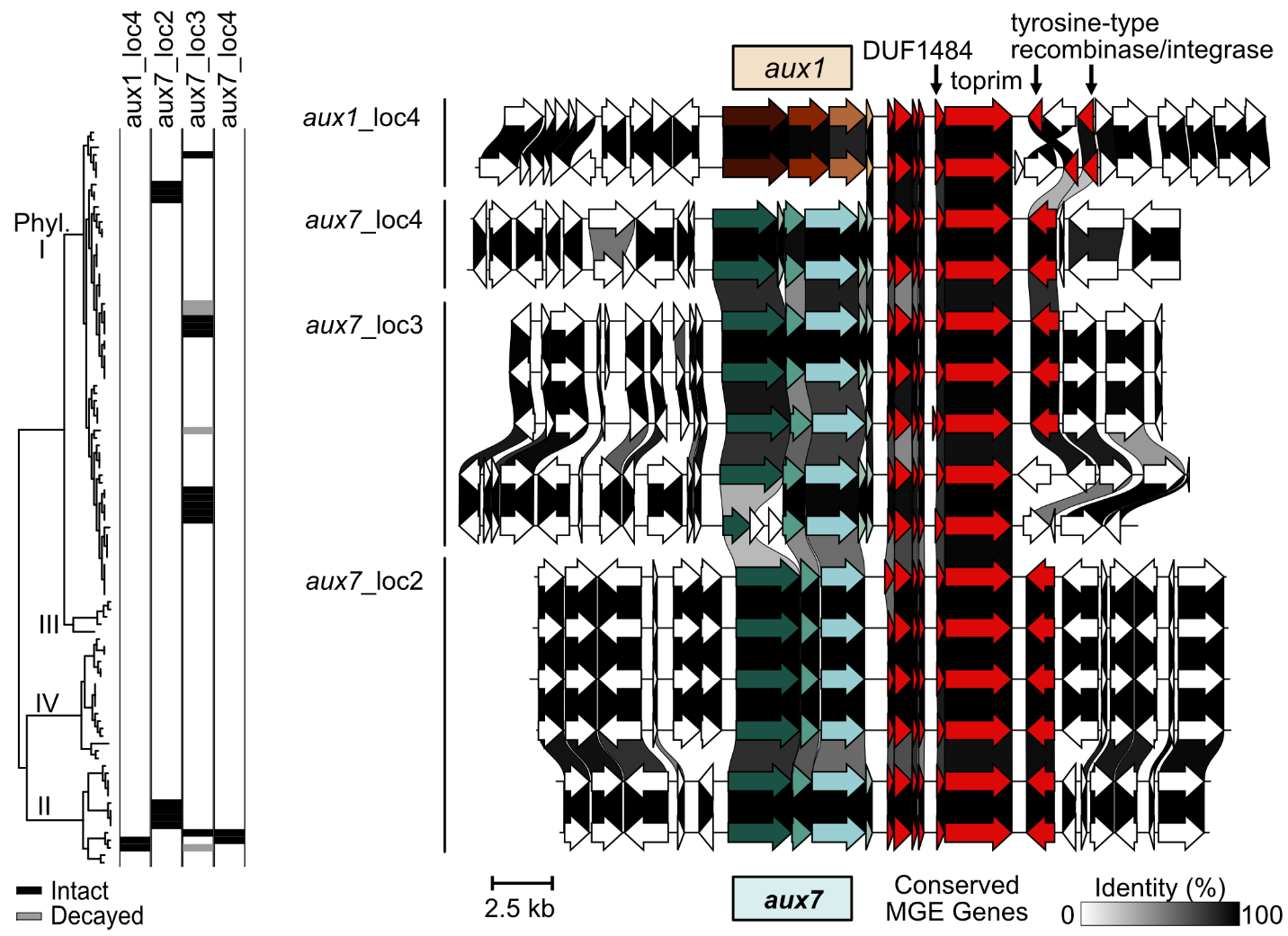


**Figure S34. *Aux1* and *aux7* are sometimes located in a toprim/DUF1484 mobile genetic element (MGE).** Synteny was visualized with Clinker. Conserved genes annotated with MGE functions are colored red. White genes show the genetic neighborhood where the MGE is located.
